# Changes in the liver transcriptome of farmed Atlantic salmon (*Salmo salar*) fed experimental diets based on terrestrial alternatives to fish meal and fish oil

**DOI:** 10.1186/s12864-018-5188-6

**Published:** 2018-11-03

**Authors:** Albert Caballero-Solares, Xi Xue, Christopher C. Parrish, Maryam Beheshti Foroutani, Richard G. Taylor, Matthew L. Rise

**Affiliations:** 1 Department of Ocean Sciences, Memorial University of Newfoundland, 1 Marine Lab Road, St. John’s, NL A1C 5S7 Canada; 2 Cargill Innovation Center, 4335 Dirdal, Norway

**Keywords:** Atlantic salmon, Marine ingredients, Terrestrial ingredients, Aquaculture, Nutrigenomics, Liver transcriptome, Microarray, Lipid composition, Cholesterol, Predictive biomarkers

## Abstract

**Background:**

Dependence on marine natural resources threatens the sustainability of Atlantic salmon aquaculture. In the present study, Atlantic salmon fed for 14 weeks with an experimental diet based on animal by-products and vegetable oil (ABP) exhibited reduced growth performance compared with others fed a fish meal/fish oil based experimental diet (MAR) and a plant protein/vegetable oil-based experimental diet (VEG). To characterize the molecular changes underlying the differences in growth performance, we conducted a 44 K microarray study of the liver transcriptome of the three dietary groups.

**Results:**

The microarray experiment identified 122 differentially expressed features (Rank Products, PFP < 10%). Based on their associated Gene Ontology terms, 46 probes were classified as metabolic and growth-relevant genes, 25 as immune-related, and 12 as related to oxidation-reduction processes. The microarray results were validated by qPCR analysis of 29 microarray-identified transcripts. Diets significantly modulated the transcription of genes involved in carbohydrate metabolism (*gck* and *pfkfb4*), cell growth and proliferation (*sgk2* and *htra1*), apoptosis (*gadd45b*), lipid metabolism (*fabp3*, *idi1*, *sqs*), and immunity (*igd*, *mx*, *ifit5*, and *mhcI*). Hierarchical clustering and linear correlation analyses were performed to find gene expression patterns among the qPCR-analyzed transcripts, and connections between them and muscle and liver lipid composition. Overall, our results indicate that changes in the liver transcriptome and tissue lipid composition were driven by cholesterol synthesis up-regulation by ABP and VEG diets, and the lower carbohydrate intake in the ABP group. Two of the microarray-identified genes (*sgk2* and *htra1*) might be key to explaining glucose metabolism regulation and the dietary-modulation of the immune system in fish. To evaluate the potential of these genes as predictive biomarkers, we subjected the qPCR data to a stepwise discriminant analysis. Three sets of no more than four genes were found to be able to predict, with high accuracy (67–94%), salmon growth and fatty acid composition.

**Conclusions:**

This study provides new findings on the impact of terrestrial animal and plant products on the nutrition and health of farmed Atlantic salmon, and a new method based on gene biomarkers for potentially predicting desired phenotypes, which could help formulate superior feeds for the Atlantic salmon aquaculture industry.

**Electronic supplementary material:**

The online version of this article (10.1186/s12864-018-5188-6) contains supplementary material, which is available to authorized users.

## Background

World aquaculture production continues to rely on the supply of fish meal (FM) and fish oil (FO) from overexploited fish stocks to feed farmed fish [1, 2]. Research on replacement of FM and FO with alternative ingredients has made notable progress since economic and ecological sustainability of aquafeeds became a growing concern for industry and consumers [2]. In recent years, traditional methods for evaluating feed performance like the monitoring of morphometric and biochemical parameters have been complemented by molecular techniques [3]. The nutrigenomic approach has facilitated the understanding of the physiological mechanisms behind impaired growth rates of fish fed diets low in marine ingredients [4]. However, the dietary modulation of some metabolic and immune pathways in fish is yet to be elucidated [4, 5], and high FM/FO replacement levels in feeds can be challenging for carnivorous fish growth performance [6], although significant advances have recently been made in this direction [7, 8].

Several different alternative ingredients to FM and FO have been tested on farmed fish [9]. Soy protein concentrate (SPC) has become an extensively used ingredient in commercial aquafeeds due to its high protein proportion and low contents of indigestible fibers and anti-nutrients, nutritional characteristics that contribute to better performance compared with plant meals [10–12]. Furthermore, Atlantic salmon (*Salmo salar*) have been found to grow well on diets with moderate replacement levels of FM by SPC [13]. Other plant ingredients with high protein content are wheat and corn glutens, which have proven to be nutritionally valuable when combined with oilseed protein sources [14, 15]. Rendered terrestrial animal products can also be used as an alternative protein source to FM [9, 16]. Meals from animal by-products often present adequate amino acid profiles compared with plant protein sources, higher levels of digestible phosphorus, and competitive prices and availability in the global market. However, use of these products is not well accepted by some consumers because of health concerns in the past (e.g., bovine spongiform encephalopathy) [9]. Nevertheless, the use of animal by-products in aquafeeds is widespread, and their quality has improved greatly since the 1980s with the refinement of the production methods [17]. Recent work on salmonids has shown that FM can be replaced at high levels by poultry by-products without affecting growth performance [18, 19]. However, in contrast to plant protein sources, there is a lack of studies on the effects of these feedstuffs on the fish transcriptome.

FO in aquafeeds can effectively be replaced at high levels by vegetable oils without significant detriment to fish growth performance [14, 20–24]. Despite the apparent positive results, lipid class and fatty acid profiles of vegetable oils may negatively impact the welfare of the fish and their quality as a commodity (nutritional value and flavor). Most vegetable oils are known to be devoid of omega-3 long-chain polyunsaturated fatty acids (ω3 LC-PUFAs) such as eicosapentaenoic acid (EPA) and docosahexaenoic acid (DHA), and to have high content of omega-6 fatty acids (ω6 FAs) [25], a combination known to be pro-inflammatory [26, 27]. Differences in diet ratios of ω6 and ω3 FAs, especially arachidonic acid (ARA)/EPA, have been found to modulate the inflammatory and immune responses of Atlantic salmon [28–30]. Indeed, among all farmed fish species, diet FA composition takes on particular importance for Atlantic salmon producers and consumers. Atlantic salmon is a highly valued product due to the elevated content of EPA (20:5ω3) and DHA (22:6ω3) in the flesh. Besides their anti-inflammatory properties, EPA and DHA are also essential for the proper functioning of important physiological processes in humans and fish [31–33]. Low FO levels in feeds can result in reduced levels of ω3 LC-PUFAs in muscle of Atlantic salmon, as salmonids have limited capacity for de novo synthesis of such metabolites [34–36]. This was shown recently by Sprague et al. [25] for Scottish farmed salmon: the EPA and DHA levels in salmon flesh in 2015 had decreased to almost half the values of 2006’s production. Several solutions have been proposed to break the interdependency of the use of more sustainable oil sources and the decline of the nutritional value of Atlantic salmon. The culture of transgenic fish [37, 38], and the use of transgenic plants [23] and new oil sources [35, 36, 39], have been studied. Regardless, for all of the potential paths to address the problem, a better understanding of lipid metabolism in Atlantic salmon (and fish in general) is needed.

In 2013, the main suppliers of aquafeeds for farmed Atlantic salmon (EWOS, Skretting, and BioMar) included 18% FM and 11% FO in their formulations [40]. According to FAO’s estimations [1], over the period 2016–2025 FM inclusion in salmon feeds will be around 15–10%. However, these estimations are subjected to multiple uncertainties such as adverse climatic conditions (i.e., climate change impacts), or changes in the economic and social environment [1]. Therefore, more ambitious replacement levels need to be explored. In the present study, we investigated the effects of almost completely replacing marine ingredients with terrestrial animal and plant alternatives on the liver transcriptome of Atlantic salmon. We correlated growth performance and lipid composition with the transcription of diet-responsive genes, which has allowed us to identify phenotype-predicting gene expression biomarkers. Taken together, our study not only provides new insights into the nutrition (and health) of Atlantic salmon under challenging dietary conditions, but also new tools for industry to assess the performance of future aquafeed candidates.

## Methods

### Experimental diets and animals

The present study was conducted using liver RNA samples from Atlantic salmon fed three experimental diets with different combinations of protein and oil sources for 14 weeks. The diets were designed by EWOS Innovation (now Cargill Innovation) and were as follows: a diet with a high proportion of FM and FO (MAR diet), a high animal by-product/high rapeseed oil diet (ABP diet), and a high plant protein/high rapeseed oil diet (VEG). Regardless of the source of the ingredients utilized, all feeds were formulated to be isonitrogenous and isoenergetic and to meet the nutritional requirements of salmonids [41]. The formulation of MAR, ABP, and VEG diets was published in previous investigations [28, 42]. However, since diet formulation is pertinent to the present study, it is provided herein (Table 1).

**Table 1:** Formulation (%) of the experimental diets fed to Atlantic salmon during the feeding trial and selected in the present and a previous study [28, 42]

Ingredient (%)^a^	MAR	ABP	VEG
Fish meal	34.5	5.0	5.0
Poultry by-product meals^b^	14.6	33.3	10.3
Corn gluten	1.4	3.4	7.0
Soy protein concentrate	7.0	17.2	35.0
Wheat gluten	0.9	2.3	4.7
Fish oil	12.4	5.1	5.1
Rapeseed oil	6.2	14.4	17.3
Raw wheat	22.6	18.2	12.7
Premix^c^	0.5	1.1	3.0
Total	100.0	100.0	100.0

^a^All ingredients were sourced from EWOS stocks

^b^For confidentiality, the nature and proportions of the poultry by-product meals included in the diets are not provided

^c^Premix includes vitamins, trace elements and inorganic phosphorus. Composition in micronutrients of the premix is proprietary information to Cargill Innovation (formerly EWOS Innovation)

For the feeding trial, salmon smolts from Northern Harvest Sea Farms (Stephenville, NL, Canada) were transported to the Dr. Joe Brown Aquatic Research Building (JBARB, Ocean Sciences Centre, Memorial University of Newfoundland, Canada), and held in 3800-L tanks. For individual identification, the salmon were PIT (passive integrated transponder)-tagged. Once adapted to the holding conditions at JBARB (flow-through seawater system, 12-h light photoperiod, and water temperature of ~ 12 °C) and having reached the desired weight, the Atlantic salmon were randomly distributed in twenty-eight 620-L tanks, with 40 fish each. Fish were considered as adapted to holding conditions once accepting a feed ration of 1% wet body weight per day. After 2 weeks of acclimation to the 620-L tanks, salmon feeding switched from the commercial diet (Nutra Transfer NP, 3 mm, Skretting Canada, St. Andrews, NB, Canada) to the experimental diets. Salmon weight at the beginning of the trial was 179 ± 29 g (mean value ± standard deviation); no significant differences were found across tanks (one-way ANOVA). Four tanks were dedicated to each experimental diet, which were manually supplied to the fish to apparent satiation twice a day for 14 weeks. Apparent feed intake, and water temperature and oxygen levels, were recorded daily. Salmon weight was measured at the beginning and at the end of the feeding trial to monitor differences in growth performance.

### Tissue collection and sample selection

At the end of the trial, salmon were starved for 24 h and then five individuals per tank were euthanized with an overdose of MS-222 (400 mg/L; Syndel Laboratories, Vancouver, BC, Canada) and dissected for tissue collection. After collection, liver samples of 50–100 mg were immediately flash-frozen in liquid nitrogen and stored at − 80 °C until processing for RNA extraction. Liver and muscle samples for the determination of lipid classes and fatty acid composition were collected, processed, and analyzed as described in Hixson et al. [36].

Biological variability can strongly determine fish performance on a given diet, and thus complicate the finding of common dietary-related transcriptomic patterns among specimens. For this reason, we decided only to utilize liver samples from salmon showing growth performances close to the tank average. Therefore, we selected samples from those fish with weight gains within one standard deviation below and above the mean value of the tank. Tank average and not diet average was chosen for sample selection so that inter-tank variability could be included in our statistical contrasts.

### RNA extraction, DNase treatment, and column purification

Liver samples were homogenized in TRIzol Reagent (Invitrogen/Life Technologies, Burlington, Canada) and subjected to RNA extraction as described previously in Xue et al. [35] and Xu et al. [43]. Thirty micrograms of each total RNA sample were treated with 6.8 Kunitz units of DNaseI (RNase-Free DNase Set, QIAGEN) with the manufacturer’s buffer (1X final concentration) at room temperature for 10 min to degrade any residual genomic DNA. DNase-treated RNA samples were column-purified using the RNeasy Mini Kit (QIAGEN) following the manufacturer’s instructions. RNA integrity was verified by 1% agarose gel electrophoresis, and RNA purity was assessed by A260/280 and A260/230 NanoDrop UV spectrophotometry (NanoDrop, Wilmington, DE, USA) at all stages of the RNA preparation (i.e., after Trizol-extraction, after re-extraction, and after column-purification). Column-purified RNA samples had A260/280 ratios between 2.1 and 2.3 and A260/230 ratios between 1.9 and 2.4.

### Microarray hybridization and data acquisition

Each of the three dietary groups (MAR, ABP, and VEG) contributed liver RNA samples of eight individual fish (two from each quadruplicate tank) to the microarray experiment (i.e., 24 fish in total). Differences among dietary groups in the liver transcriptome of fish were assessed by contrasting individual RNA samples against a common reference pool of equal quantities of RNA from all 24 fish. Twenty-four arrays were used for the experiment: one array for each individual/common reference RNA hybridization. The microarray experiment was performed as described in Xue et al. [35]. Briefly, anti-sense amplified RNA (aRNA) was in vitro transcribed from 1 μg of each experimental RNA or reference RNA pool using Ambion’s Amino Allyl MessageAmp™ II aRNA Amplification kit (Life Technologies), following the manufacturer’s instructions. The quality and quantity of aRNA were assessed using NanoDrop spectrophotometry and agarose gel electrophoresis. Twenty micrograms of aRNA were subsequently precipitated overnight following standard molecular biology procedures and re-suspended in coupling buffer. Individual aRNA samples were labeled with Cy5, whereas reference aRNA samples were labeled with Cy3 (GE HealthCare, Mississauga, ON), following the manufacturer’s instructions. The labeling efficiency was measured using the “microarray” function of the NanoDrop spectrophotometer. For each array, an equal quantity (825 ng) of an individual Cy5-labeled and a reference Cy3-labeled aRNA sample were fragmented and co-hybridized to a consortium for Genomic Research on All Salmonids Project (cGRASP)-designed Agilent 44 K salmonid oligonucleotide microarray (GEO accession number: GPL11299) [44], following manufacturer instructions (Agilent, Mississauga, ON). The arrays were hybridized at 65 °C for 17 h with 10 rpm rotation in an Agilent hybridization oven. The array slides were washed immediately after hybridization as per the manufacturer’s instructions.

The microarray slides were scanned at 5 μm resolution with 90% laser power using a ScanArray Gx Plus scanner and ScanExpress v4.0 software (Perkin Elmer, Waltham, Massachusetts, USA), and the Cy3 and Cy5 channel photomultiplier tube (PMT) settings were adjusted to balance the fluorescence signal. The raw data contained in the TIFF images resulting from the scanning were extracted using Imagene 9.0 (BioDiscovery, El Segundo, California, USA). The removal of low-quality/flagged spots on the microarray, as well as the background signal correction, the log_2_-transformation and Loess-normalization of the data, were performed using R and the Bioconductor package mArray [45]. Features absent in more than 30% of the arrays were discarded, and the missing values were imputed using the EM_array method and the LSimpute package [35, 46, 47]. The final dataset used for statistical analyses consisted of 11,149 probes for all arrays (GEO accession number: GSE106604; https://www.ncbi.nlm.nih.gov/geo/query/acc.cgi?acc=GSE106604).

### Microarray data analysis

The differentially expressed genes among diets were determined using Rank Products (RP), a non-parametric statistical method that is less sensitive to high biological variability than Significance Analysis of Microarrays (SAM) [47–50]. RP analysis was conducted at a percentage of false-positives (PFP) threshold of 10%, using the Bioconductor package, RankProd [51]. The resulting gene lists were annotated using the contiguous sequences (contigs) employed to design the 60mer oligonucleotide probes of the array [44]. Annotation was carried out manually with BLASTx/BLASTn searches against the NCBI non-redundant (nr) amino acid and nucleotide sequence databases using an *E*-value threshold of 10^− 5^. The best BLASTx hits corresponding to putative *Danio rerio* or *Homo sapiens* orthologues were used to obtain gene ontology (GO) terms from the UniProt Knowledgebase (http://www.uniprot.org/).

### qPCR analysis

We selected 29 genes of interest (GOIs) from the list of microarray-identified genes for real-time quantitative polymerase chain reaction (qPCR) analysis. Gene selection was based on the different metabolic pathways or biological processes represented among the diet-responsive identified genes. Three more GOIs not identified by the microarray analysis (i.e., *acyl-coenzyme A oxidase 1*, *carnitine palmitoyltransferase 1*, and *ATP-citrate synthase*) were added to the qPCR experiment. For the qPCR analysis, we also included seven additional RNA samples from the three dietary treatments (two from MAR group, two from ABP, and three from VEG; 31 samples in total), all of them satisfying the criterion for sample selection (see above in ‘Tissue collection and sample selection’ section).

For each of the selected GOIs, we BLASTn-searched for paralogues using published Atlantic salmon cDNA sequences in the non-redundant nucleotide (nt) sequence and the expressed sequence tags (EST) databases of NCBI. BLASTn-searches were performed between October and November, 2015, and thus limited to the sequences available during that period. Putative paralogue sequences were compiled and aligned using Vector NTI (Vector NTI Advance 11, Life Technologies) to determine identity between paralogues and find suitable regions where to design paralogue-specific qPCR primers (Additional file 1). Paralogue-specific primers were intended to amplify areas with at least 2 bp difference between sequences to ensure specificity. Primers not complying with this rule (i.e., 1 bp difference) were paired with primers targeting more heterogeneous areas of the cDNA sequence. All primers were designed using Primer 3 v.0.4.0 software [available at (http://bioinfo.ut.ee/primer3-0.4.0/)]. Each primer pair was quality tested using the 7500 Fast Real Time PCR system (Applied Biosystems/Life Technologies). Quality testing ensured that a single product was amplified (dissociation curve analysis) and that there was no primer-dimer present in the no-template control. Amplicons were electrophoretically separated on 2% agarose gels and compared with a 1 kb Plus DNA Ladder (Invitrogen/Life Technologies) to verify that the correct size fragment was being amplified. Amplification efficiencies [52] were determined using 5-point 1:3 dilution series starting with cDNA representing 10 ng of input total RNA. For primer quality testing, a reference RNA pool was prepared with an equal contribution of all specimens included in the qPCR study.

Transcript (mRNA) levels of the GOIs were normalized against two endogenous control genes. To select these endogenous controls, qPCR primer pairs were designed for six candidate normalizers and quality tested as described above. Two of the candidate normalizers genes were selected as they were among the most stable transcripts represented in our 11,149 microarray probe dataset: *transmembrane protein 85*, and *ATPase H+ transporting V1 subunit E1*. The other candidate normalizers were chosen based on Atlantic salmon literature [53] and previous studies of our group [35] (*60S ribosomal protein L32*, *β-actin*, and two paralogues of *elongation factor 1-alpha*). The fluorescence threshold cycle (C_T_) values of the 31 samples were measured for each of these genes using cDNA representing 5 ng of input total RNA and then analyzed using *geNorm* (qBASE plus, Biogazelle NV, Belgium) [54]. Using this software, *60S ribosomal protein L32* (*rpl32*; *geNorm* M = 0.255) and *elongation factor 1-alpha 1* (*eef1a1*; *geNorm* M = 0.250) were determined to be the most stable.

First-strand cDNA templates for qPCR were synthesized in 20 μL reactions from 1 μg of DNaseI-treated, column-purified total RNA using random primers (250 ng; Invitrogen/Life Technologies) and M-MLV reverse transcriptase (200 U; Invitrogen/Life Technologies) with the manufacturer’s first strand buffer (1X final concentration), dNTPs (0.5 mM final concentration), and DTT (10 mM final concentration) at 37 °C for 50 min. Once primer quality testing was completed, the transcript levels of the selected GOIs and normalizer genes were qPCR-analyzed in technical triplicates using Power SYBR Green I dye chemistry in 384-well format on a ViiA 7 Real Time PCR system (Applied Biosystems/Life Technologies, Foster City, CA). In all qPCR analyses, a no-template control (in triplicate) was included in the plate. The qPCR amplifications were performed in 13 μL reactions using 1X Power SYBR Green PCR Master Mix (Applied Biosystems/Life Technologies), 50 nM of both the forward and reverse primers, and 4 μL of diluted cDNA (5 ng input total RNA). The qPCR program consisted of 1 cycle of 50 °C for 2 min, 1 cycle of 95 °C for 10 min, and 40 cycles of 95 °C for 15 s and 60 °C for 1 min, with fluorescence detection at the end of each 60 °C step.

The relative quantity (RQ) of each transcript was determined using the ViiA 7 Software Relative Quantification Study Application (Version 1.2.3) (Applied Biosystems/Life Technologies), with normalization to both *rpl32* and *eef1a1* transcript levels, and with amplification efficiencies incorporated. For each GOI, the sample with the lowest normalized expression (mRNA) level was set as the calibrator sample (i.e., assigned an RQ value = 1).

Fold-change values calculated from microarray log_2_ ratios, and log_2_-transformed qPCR RQs, were analyzed for correlation via linear regression (see below). The formula used to calculate gene expression fold-changes between a dietary group (A) and another (B) was 2^A-B^ [55, 56]. For down-regulated genes, fold-change values were inverted (− 1/fold-change). A significant correlation between both datasets was considered as proof of the validity of the microarray results.

### Statistical analyses

#### Comparing mean values among dietary groups

GOI RQs, growth performance (i.e., weight gain and AFI) and tissue composition (i.e., lipid class and FA profiles) data for one-way ANOVA were first checked for outliers using Grubb’s test, and for homoscedasticity using Levene’s test. The ‘Tank’ effect was nested to ‘Diet’ factor in the one-way ANOVA. If variances were found not to be statistically equal among dietary groups (failure to comply with the homoscedasticity assumption), Games-Howell tests were used for pair-wise comparisons. If the homoscedasticity assumption was satisfied, the mean values of the three dietary treatments were compared using Tukey’s post hoc test. The accepted level of significance was *p* < 0.05. All the statistical analyses above were conducted using IBM SPSS Statistics (version 24.0.0; IBM Corp, Armonk, NY).

#### Linear regression analyses and hierarchical clustering

Differences in dietary lipid composition could lead to contrasted tissue lipid class and FA profiles, as well as changes in the liver transcriptome of the salmon. Therefore, we analyzed the relationships between all three datasets (qPCR results, and tissue lipid class and FA composition) for each individual fish by linear regression analysis. In this regression analysis, we included only GOIs showing significant differences among diets, sterols based upon our transcriptomic data, and those ω3 and ω6 FAs accounting for at least 0.5% of the total FAs in the tissue. Fish weight gain and other relevant tissue composition parameters (e.g., EPA/ARA ratios) were also included. To facilitate the discussion of the possible correlation patterns, GOIs and tissue composition parameters were grouped by hierarchical clustering. The methods and results of these tissue composition analyses are published elsewhere [42].

Differences in feed intake can have a significant effect on fish gene expression [57–59]. Therefore, we analyzed the possible correlation between the qPCR results and AFI values of each tank (as AFI cannot be calculated for each individual fish) by linear regression.

For linear regression analyses and hierarchical clustering, all datasets were log_2_-transformed to meet the normality assumption (checked by Kolmogorov-Smirnov test). The linear regression analyses were performed with IBM SPSS Statistics, whereas the complete linkage hierarchical clustering was carried out with PRIMER (Version 6.1.15, Ivybridge, UK) using Pearson correlation resemblance matrices. The significance of the regression was determined by an F-test (*p* < 0.05).

#### Stepwise discriminant analysis

In addition to the statistical analyses described above, we analyzed our qPCR results in search of potential phenotype-predictive biomarkers. A stepwise discriminant analysis was deemed to be the best method. Discriminant analysis linearly combines variables into predictive functions to classify experimental subjects between different levels of a categorical variable. Discriminant scores are calculated using the predictive functions and used to classify individuals based on cut-off values determined by maximum likelihood [60]. As categorical variables, we considered ‘Diet’ (MAR, ABP, and VEG), ‘Growth’ (weight gain; low, and high), and the sum of DHA and EPA levels in the muscle (‘EPA + DHA’; low, and high). The number of individual samples available did not allow the division of ‘Growth’ and ‘EPA + DHA’ values in more than two levels. In both cases, values above the mean across individuals were denoted as ‘high’, whereas those values below the mean were ‘low’. The number of predictive functions that a discriminant analysis can generate equals the number of levels of the categorical variable minus 1. We chose the stepwise method for the selection of the predictor variables (i.e., diet-responsive GOIs). Thus, GOIs were entered provided the predictive ability of the function (measured as Wilks’ lambda statistic) was significantly improved (minimum partial F-to-enter = 3.84 [61]), and removed if significantly reduced (maximum partial F-to-remove = 2.81). The accuracy of the predictive functions was assessed using a leave-one-out classification method. This method classifies each individual by the functions derived for all subjects except the one being classified [62]. In this way, we obtain a less biased estimate of the true predictive ability of the functions. Only log_2_-transformed RQ values of diet-responsive transcripts were included in the analysis. We used IBM SPSS Statistics to conduct the stepwise discriminant analyses.

## Results

### Diet effect on fish growth performance

The feeding trial concluded with salmon fed MAR diet showing significantly higher final weight and weight gain than ABP diet (Table 2). In contrast, based on final weight and weight gain, VEG diet performed similarly to MAR diet. However, the hepatosomatic index (HSI) was significantly higher in fish fed MAR diet than in fish fed VEG diet. Salmon fed ABP diet showed a trend towards lower apparent feed intake (AFI), but the differences among group mean values were not statistically significant (*p* = 0.069). Results in Table 2 were previously published [28, 42]. However, they are included herein as also pertinent to this study, as were used in the selection of the three dietary groups for the transcriptomic analysis.

**Table 2:** Growth parameters of the Atlantic salmon fed the three experimental diets

Diet	MAR	ABP	VEG
Initial Weight (g)	176.8 ± 29.4	179.2 ± 28.8	177.2 ± 29.8
Final Weight (g)	342.5 ± 89.5^a^	316.3 ± 63.6^b^	332.9 ± 79.2^ab^
Weight Gain^1^ (g)	165.6 ± 77.5^a^	137.1 ± 47.5^b^	155.7 ± 64.6^ab^
HSI^2^ (%)	1.49 ± 0.40^a^	1.29 ± 0.38^ab^	1.19 ± 0.25^b^
AFI^3^ (g fish^− 1^)	178.7 ± 25.2	146.6 ± 5.5	167.4 ± 14.4

Values are expressed as mean ± standard deviation calculated from 134 to 138 individual fish of each dietary group for weight parameters, 29–32 fish for HSI, and 4 tanks of each dietary group for AFI. Different superscripts in the same row indicate significant differences among diets (one-way ANOVA, Tukey’s post-hoc test, *p* < 0.05)

^1^Weight gain = Final weight – Initial weight

^2^Hepatosomatic index = 100 × (liver mass/body mass)

^3^Apparent feed intake = feed consumption/number of fish per tank

### Microarray profiling of fish liver transcriptome

One hundred and twenty-two differentially expressed probes were detected in the microarray analysis of the liver transcriptome of fish fed the three experimental diets (Additional file 2). Based on their associated Gene Ontology (GO) terms and the information available in the literature, 83 BLAST-identified and functionally characterized probes were classified as genes involved in metabolic pathways (e.g., carbohydrate metabolism, lipid metabolism) and biological processes (e.g., autophagy, antibacterial response) (Tables 3, 4, 5 and 6). The other 39 differentially expressed probes did not fall into any of these categories or encoded putative unknown or uncharacterized proteins and, therefore, were classified as miscellaneous and unknown (see Additional file 2).

**Table 3:** List of microarray features representing genes involved in nutrient metabolism that were differentially expressed in the liver of Atlantic salmon fed diets with different proportions of marine and terrestrial ingredients

Probe ID^a^	Best named BLASTx/BLASTn hit [species]^b^	Functional annotation^c^	ABP vs MAR^d^	VEG vs MAR^d^	VEG vs ABP^d^
Carbohydrate metabolism					
C035R008	Glucokinase ( *gck* ) [ *Salmo marmoratus* ]	P: glycolytic process	**−2.4**	**−1.7**	1.4
C185R084	Predicted: 6-phosphofructo-2-kinase/fructose-2,6-bisphosphatase isoform X2 ( *pfkfb4* ) ^e^ [ *S. salar* ]	P: fructose metabolic process	**−1.9**	− 1.7	1.1
C113R060	Predicted: 6-phosphofructo-2-kinase/fructose-2,6-bisphosphatase isoform X2 ( *pfkfb4* ) ^e^ [ *S. salar* ]	P: fructose metabolic process	−1.7	**− 1.9**	−1.1
C043R025	Fructose-bisphosphate aldolase B [*S. salar*]	P: glycolytic process	− 1.4	**−1.9**	− 1.3
C169R002	Predicted: 6-phosphogluconate dehydrogenase, decarboxylating ( *6pgd* ) [ *S. salar* ]	P: pentose-phosphate shunt, oxidative branch	−1.3	**−2.0**	− 1.6
C039R032	Predicted: 6-phosphogluconate dehydrogenase, decarboxylating ( *6pgd* ) [ *S. salar* ]	P: pentose-phosphate shunt, oxidative branch	−1.0	**−1.8**	− 1.7
C128R035	Predicted: glucose-6-phosphate 1-dehydrogenase-like (LOC106571947), transcript variant X3 [*S. salar*]*	P: pentose-phosphate shunt, oxidative branch	−1.1	**−1.5**	− 1.3
Lipid metabolism					
C134R089	Predicted: diacylglycerol O-acyltransferase 2-like (LOC106567948) ( *dgat2b* ) [ *S. salar* ]*	P: triglyceride biosynthetic process	**−1.5**	**−2.7**	**− 1.8**
C103R066	Predicted: diacylglycerol O-acyltransferase 2 ( *dgat2b* ) [ *S. salar* ]	P: triglyceride biosynthetic process	−1.3	**−2.0**	− 1.6
C050R151	Predicted: acetyl-CoA carboxylase isoform X7 ( *acac* ) [ *S. salar* ]	P: fatty acid biosynthetic process	−1.3	**−2.4**	**− 1.8**
C086R144	Fatty acid-binding protein, heart ( *fabp3* ) [ *S. salar* ]	P: long-chain fatty acid transport	1.2	**−1.6**	**−1.9**
C262R025	Predicted: isopentenyl-diphosphate Delta-isomerase 1 isoform X1 ( *idi1* ) [ *S. salar* ]	P: cholesterol biosynthetic process	**1.7**	**1.9**	1.1
C130R156	Predicted: insulin-induced gene 1 protein-like [*S. salar*]	P: cholesterol biosynthetic process	**2.4**	1.6	−1.5
C069R129	Fatty acyl-CoA reductase 1 [*S. salar*]	P: glycerophospholipid biosynthetic process	**1.9**	1.4	−1.4
C078R087	Farnesyl pyrophosphate synthetase [*S. salar*]	P: cholesterol metabolic process	**1.8**	1.8	1.0
C229R065	Predicted: squalene synthase isoform X2 ( *sqs* ) [ *S. salar* ]	P: cholesterol biosynthetic process	**1.9**	1.7	−1.1
C025R006	Predicted: hydroxymethylglutaryl-CoA synthase, cytoplasmic-like (LOC106571543), transcript variant X4 [*S. salar*]*	P: cholesterol biosynthetic process	**1.8**	1.4	−1.3
C067R143	Predicted: fatty acid hydroxylase domain-containing protein 2 [*S. salar*]	P: fatty acid biosynthetic process	**−2.2**	− 1.4	1.6
C089R080	Predicted: apolipoprotein B-100 precursor [*S. salar*]	P: lipid transport	**−1.6**	− 1.3	1.2
C190R063	Predicted: lipid phosphate phosphohydrolase 1 [*S. salar*]	P: lipid metabolic process	1.7	**2.2**	1.3
C188R006	Predicted: long-chain-fatty-acid--CoA ligase ACSBG2 [*S. salar*]	P: long-chain fatty acid metabolic process	−1.7	**−2.5**	− 1.4
C104R105	Predicted: adiponectin precursor [*S. salar*]	P: fatty acid beta-oxidation; P: glucose homeostasis	−1.5	**−2.0**	− 1.3
C085R162	CCAAT/enhancer-binding protein alpha [*S. salar*]	P: lipid homeostasis; P:liver development	− 1.2	**− 1.8**	− 1.5
C004R046	Predicted: fatty acid synthase [*S. sala*r]	P: fatty acid biosynthetic process	−0.8	−1.3	**−1.6**
Nucleotide metabolism					
C098R022	Adenylosuccinate synthetase isozyme 1 C ( *adssl1a* ) [ *S. salar* ]	P: ‘de novo’ AMP biosynthetic process	**− 1.9**	**−2.6**	− 1.4
C107R157	Predicted: adenylosuccinate synthetase isozyme 1 C-like (*adssl1b*)^f^ [*S. salar*]	P: ‘de novo’ AMP biosynthetic process	**−1.7**	**−2.5**	− 1.5
C016R136	Predicted: CTP synthase 1-like [*S. salar*]	P: ‘de novo’ CTP biosynthetic process	− 1.3	**− 2.3**	− 1.7
C086R065	Predicted: kalirin-like isoform X6 [*S. salar*]	F: guanyl-nucleotide exchange factor activity	−1.2	1.7	**2.0**

^a^Refers to the identifier of the probe on the 44 K array

^b^The contiguous sequences (contigs) from which the microarray probes were designed were used for gene identification by BLASTx against the NCBI nr database. The name of the protein [species] of the BLASTx hit with the lowest E-value (cutoff value of 10^− 5^) is presented. If no reliable BLASTx hits were found, the best BLASTn hit was chosen instead (E-value cutoff of 10^− 10^). BLASTn-identified genes are denoted with an asterisk. The names of those genes for which transcript levels were analyzed by real-time quantitative polymerase chain reaction (qPCR) are underlined. For the qPCR-analyzed genes, the gene symbol is indicated in brackets, differentiating paralogues if possible

^c^Gene ontology (GO) terms selected to functionally annotate the cDNA’s best BLASTx/BLASTn hit, obtained from putative *Danio rerio* and *Homo sapiens* orthologues (i.e., best BLASTx hit from these model species). GO terms were obtained from UniProt Knowledgebase. GO categories: biological process (P), molecular function (F), and cellular component (C)

^d^Fold-change (FC) values between two dietary treatments, calculated from microarray log_2_ ratios for a given probe (formula: 2^A-B^ [55, 56]). Significant differences (PFP < 10%) between treatments are indicated by bolding the FC values

^e^qPCR primers were designed based on a cDNA sequence for *Salmo salar* 6-phosphofructo-2-kinase/fructose-2,6-biphosphatase 4-like (BT044025), which shares 100% identity with the 60mer microarray probes. See Additional file 2 for additional information regarding BLASTx/BLASTn statistics and identity among the nucleotide sequences used for designing the microarray and qPCR probes

^f^*Adssl1a* and *adssl1b* paralogues showed 93% identity over 597 aligned nucleotides (see Additional file 3). Paralogue *adssl1b* was not qPCR-analyzed because none of the designed primer pairs passed quality testing due to low transcript levels (i.e., 10 ng of input total RNA yielding Ct values above 31)

**Table 4:** List of microarray features representing genes involved in cellular processes that were differentially expressed in the liver of Atlantic salmon fed diets with different proportions of marine and terrestrial ingredients

Probe ID^a^	Best named BLASTx/BLASTn hit [species]^b^	Functional annotation^c^	ABP vs MAR^d^	VEG vs MAR^d^	VEG vs ABP^d^
Protein synthesis and degradation					
C152R057	Ribosomal protein L18a [*S. salar*]	P: translation	**2.2**	1.6	−1.3
C027R084	Predicted: trypsin-2 [*S. salar*]	P: proteolysis	**2.0**	1.8	−1.1
C097R072	Trypsin precursor [*S. salar*]	P: proteolysis	**1.9**	1.2	−1.6
C172R121	60S ribosomal protein L7 [*S. salar*]	P: cytoplasmic translation; P: regulation of cell cycle	**−1.8**	− 1.4	1.3
C005R140	60S acidic ribosomal protein P2 [*S. salar*]	P: translational elongation	1.9	**2.4**	1.2
Cell growth and proliferation					
C168R030	Serine/threonine-protein kinase Sgk2 ( *sgk2a* ) [ *S. salar* ]	P: regulation of cell growth; P: regulation of cell proliferation	**−1.9**	−1.1	**1.8**
C050R117	Serine/threonine-protein kinase Sgk2 ( *sgk2b* ) [ *S. salar* ]	P: regulation of cell growth; P: regulation of cell proliferation	**−1.8**	−1.2	1.4
C231R170	Predicted: serine protease HTRA1B isoform X1 ( *htra1b* ) [ *S. salar* ]	P: regulation of cell growth	**−1.8**	**−2.2**	−1.2
C265R134	Predicted: serine protease HTRA1B isoform X2 ( *htra1b* ) [ *S. salar* ]	P: regulation of cell growth	**−2.0**	**−2.1**	−1.1
C170R142	Predicted: serine protease HTRA1B isoform X1 ( *htra1b* ) [ *S. salar* ]	P: regulation of cell growth	−1.6	**−1.8**	− 1.1
C035R053	Predicted: BicC family RNA binding protein 1, transcript variant X7 [*S. salar*]*	P: multicellular organism development	−1.4	**− 1.6**	−1.1
C064R045	Predicted: PERQ amino acid-rich with GYF domain-containing protein 1-like [*S. salar*]	P: insulin-like growth factor receptor signaling pathway	**1.8**	1.1	−1.7
C120R149	Predicted: protein regulator of cytokinesis 1-like isoform X1 [*S. salar*]	P: positive regulation of cell proliferation	**2.0**	1.5	−1.3
C087R056	Pre-mRNA-splicing factor syf2 [*S. salar*]	P: positive regulation of cell proliferation	**2.8**	**3.6**	1.3
C240R016	Predicted: cbp/p300-interacting transactivator 2-like [*S. salar*]	P: liver development; P:cell proliferation	**−1.8**	−1.4	1.2
Autophagy and apoptosis					
C241R134	Predicted: serine/threonine-protein kinase ULK4 [*Esox lucius*]	P: regulation of p38MAPK cascade	1.2	**−1.8**	**−2.1**
C100R113	Growth arrest and DNA-damage-inducible protein GADD45 beta ( *gadd45ba* ) [ *Oncorhynchus mykiss* ]	P: positive regulation of apoptotic process	**1.9**	1.3	−1.5
C263R103	Predicted: G0/G1 switch protein 2-like [*S. salar*]	P: positive regulation of extrinsic apoptotic signaling pathway	**−1.8**	−1.5	1.2

^a,b,c,d^All column constructions are as described in Table 3

**Table 5:** List of microarray features representing immune-related genes that were differentially expressed in the liver of Atlantic salmon fed diets with different proportions of marine and terrestrial ingredients

Probe ID^a^	Best named BLASTx hit [species]^b^	Functional annotation^c^	ABP vs MAR^d^	VEG vs MAR^d^	VEG vs ABP^d^
Antibacterial					
C159R112	Leukocyte cell-derived chemotaxin 2 precursor ( *lect2* ) [ *S. salar* ]	P: response to bacterium	**−3.0**	**−1.8**	**1.7**
C134R121	Leukocyte cell-derived chemotaxin 2 precursor ( *lect2a* ) [ *S. salar* ]	P: response to bacterium	**−2.3**	**−1.9**	1.2
C164R142	Leukocyte cell-derived chemotaxin 2 precursor ( *lect2a* ) [ *S. salar* ]	P: response to bacterium	**−1.9**	− 1.3	1.5
C198R010	Hepcidin-1 precursor [*S. salar*]	P: defense response to bacterium	**−1.9**	−1.5	1.3
C201R127	Predicted: liver-expressed antimicrobial peptide 2-like [*S. salar*]	P: defense response to bacterium	−1.4	**−2.1**	−1.5
C150R092	Immunoglobulin delta heavy chain constant region ( *igd* ) [ *S. salar* ]	P: defense response to bacterium	**1.6**	−1.2	**−1.9**
C061R085	Ig mu chain C region membrane-bound form ( *igmb* ) [ *S. salar* ]	P: antibacterial humoral response	1.2	−1.3	**−1.6**
C205R051	Ig mu chain C region membrane-bound form ( *igma* ) [ *S. salar* ]	P: antibacterial humoral response	1.2	−1.3	**−1.5**
C075R137	Ig mu chain C region membrane-bound form ( *igm* ) [ *S. salar* ]	P: antibacterial humoral response	1.2	−1.3	**−1.5**
Antiviral					
C236R043	Interferon-induced GTP-binding protein Mx ( *mxb* ) [ *S. salar* ]	P: immune system process, P: response to virus	1.4	**2.1**	1.5
C055R128	Interferon-induced protein with tetratricopeptide repeats 5 ( *ifit5* ) [ *S. salar* ]	P: immune system process, P: response to virus	−1.5	1.3	**1.9**
Other immune-related					
C071R161	Predicted: alpha-1-acid glycoprotein 1-like [*Poecilia latipinna*]	P: regulation of immune system process	**−2.2**	−1.4	1.6
C097R069	MHC class I heavy chain ( *mhcI* ) [ *Salmo trutta* ]	P: antigen processing and presentation	1.1	**1.8**	1.7
C027R162	MHC class I heavy chain ( *mhcI* ) [ *Salmo trutta* ]	P: antigen processing and presentation	−1.3	**−1.6**	−1.3
C211R164	MHC class I ( *mhcI* ) [ *S. salar* ]	P: antigen processing and presentation	−1.1	1.7	**1.9**
C243R111	MHC class I heavy chain ( *mhcI* ) [ *Salmo trutta* ]	P: antigen processing and presentation	−1.3	1.5	**1.9**
C219R002	Predicted: glucosidase 2 subunit beta-like [*S. salar*]	P: innate immune response	−1.2	**− 1.8**	−1.5
C249R068	Immunoglobulin light chain precursor [*S. salar*]	P: innate immune response	−1.0	−1.8	**− 1.7**
C238R071	Predicted: C-C motif chemokine 13-like [*S. salar*]*	P: chemotaxis; P: Inflammatory response	1.0	1.8	**1.8**
C012R162	Predicted: dedicator of cytokinesis protein 11 isoform X2 [*S. salar*]	P: B cell homeostasis	**1.9**	1.8	−1.1
C121R056	Predicted: TSC22 domain family protein 3-like isoform X2 [*S. salar*]	P: negative regulation of activation-induced cell death of T cells	**1.8**	1.2	−1.4
C255R144	Ubiquitin [*S. salar*]	P: innate immune response	−1.4	1.4	**1.8**
C219R041	Predicted: peroxisomal membrane protein 2 [*S. salar*]	P: response to type I interferon	−1.4	1.3	**1.8**
C050R077	Predicted: probable E3 ubiquitin-protein ligase HERC6 [*S. salar*]	P: protein ubiquitination	−1.1	1.8	**2.0**

^a,b,c,d^All column constructions are as described in Table 3

**Table 6:** List of microarray features representing genes involved in oxidation-reduction processes that were differentially expressed in the liver of Atlantic salmon fed diets with different proportions of marine and terrestrial ingredients

Probe ID^a^	Best named BLASTx hit [species]^b^	Functional annotation^c^	ABP vs MAR^d^	VEG vs MAR^d^	VEG vs ABP^d^
Oxidation-reduction process					
C163R079	Predicted: bifunctional methylenetetrahydrofolate dehydrogenase/cyclohydrolase, mitochondrial-like [*S. salar*]	P:folic acid metabolic process; P:oxidation-reduction process	**−1.6**	1.2	**2.0**
C140R113	Predicted: bifunctional methylenetetrahydrofolate dehydrogenase/cyclohydrolase, mitochondrial-like [*S. salar*]	P:folic acid-containing compound biosynthetic process; P:oxidation-reduction process	**−1.6**	1.2	**1.9**
C060R108	Cytochrome c oxidase subunit II ( *mtco2* ) [ *Oncorhynchus masou masou* ]]	P:aerobic respiration	**1.7**	−1.0	**−1.7**
C188R069	Cytochrome oxidase subunit I ( *mtco1* ) [ *Oncorhynchus masou masou* ]	P:aerobic respiration	1.3	**−1.4**	**−1.7**
C110R041	Predicted: glycine dehydrogenase (decarboxylating), mitochondrial-like [*S. salar*]	P:glycine catabolic process; P:oxidation-reduction process	**2.0**	1.4	−1.4
C177R075	Predicted: 4-hydroxyphenylpyruvate dioxygenase-like protein [*S. salar*]	P:tyrosine catabolic process; P:oxidation-reduction process	**1.8**	1.5	−1.2
C033R147	Predicted: 1,25-dihydroxyvitamin D(3) 24-hydroxylase, mitochondrial-like [*S. salar*]	P:vitamin D catabolic process; P:oxidation-reduction process	**1.9**	1.8	−1.0
C202R025	Predicted: glutathione S-transferase theta-2B [*S. salar*]	P:glutathione metabolic process; P:oxidation-reduction process	**−1.9**	−1.2	1.6
C223R087	Predicted: cytochrome c oxidase subunit 7A-related protein, mitochondrial [*S. salar*]	P:oxidation-reduction process	−1.5	**−1.9**	− 1.3
C079R035	Predicted: saccharopine dehydrogenase-like oxidoreductase [*S. salar*]	P:platelet degranulation P:oxidation-reduction process	−1.1	**−1.6**	− 1.5
C161R028	Cytochrome P450 1A1 [*S. salar*]	P:steroid metabolic process; P:oxidation-reduction process	−1.6	**−1.9**	− 1.2
C013R125	Predicted: phospholipid hydroperoxide glutathione peroxidase, mitochondrial-like isoform X2 [*S. salar*]	P:response to oxidative stress; P:oxidation-reduction process	1.2	−1.6	**−1.8**

^a,b,c,d^All column constructions are as described in Table 3

Twenty-eight microarray probes putatively involved in nutrient metabolism were identified (Table 3). Among them, 7 probes were classified as involved in carbohydrate metabolism (e.g., *glucokinase*), 17 probes related to lipid metabolism (e.g., *fatty acid-binding protein, heart*; alias: *fatty acid-binding protein 3*), and 4 probes related to nucleotide metabolism (e.g., *adenylosuccinate synthetase*). Eighteen probes were classified as involved in cellular processes (Table 4): 5 related to protein synthesis and degradation (e.g., *trypsin*), 10 related to cell growth and proliferation (e.g., *serine/threonine-protein kinase Sgk2*), and 3 related to autophagy and apoptosis (e.g., *serine/threonine-protein kinase ULK4*). A list of 24 immune-related microarray probes was generated (Table 5), comprising 9 probes classified as antibacterial (e.g., *leukocyte cell-derived chemotaxin 2*), 2 as antiviral (e.g., *interferon-induced GTP-binding protein Mx*), and 13 as involved in other immune processes (e.g., *major histocompatibility complex class I*). Finally, the microarray experiment also identified 12 probes putatively related to oxidation-reduction processes, and therefore could serve as indicators of oxidative stress (Table 6).

Overall, microarray features identified as genes putatively related to carbohydrate metabolism were down-regulated in salmon fed the non-marine based diets (i.e., ABP and VEG diets) compared with those fed MAR diet (Table 3). Of the genes related to lipid metabolism, those involved in the metabolism of fatty acids and triacylglycerols (e.g., *diacylglycerol O-acyltransferase 2*, *acetyl-CoA carboxylase*) showed down-regulation by the non-marine diets, contrary to those participating in the synthesis of cholesterol (e.g., *squalene synthase*), which showed up-regulation by the same diets (Table 3). A feature encoding putative *heart-type fatty acid-binding protein* (*fabp3*), an intracellular lipid transport protein, and putative *apolipoprotein B-100*, an extracellular lipid transport protein, showed contrasted dietary modulation. The first, *fabp3*, was significantly down-regulated by VEG diet compared with MAR and ABP diets, while *apolipoprotein B-100* was down-regulated by ABP diet compared with MAR diet, thus resulting in an apparent higher transcription with VEG diet than with ABP diet. The microarray-identified genes related to nucleotide synthesis were mostly down-regulated by the non-marine diets.

According to the results of the microarray analysis, genes associated with proteolysis and protein synthesis (Table 4) were, in general, up-regulated by the non-marine diets. However, *60S ribosomal protein L7* transcript levels showed down-regulation by the non-marine diets, this down-regulation being statistically significant for ABP diet. Similarly, microarray-identified genes related to cell growth were largely down-regulated by non-marine diets. Three genes annotated with GO terms for cell proliferation showed opposed trends as two were up-regulated (i.e., *protein regulator of cytokinesis 1-like isoform X1* and *pre-mRNA-splicing factor syf2*) while the other one was down-regulated (i.e., *cbp/p300-interacting transactivator 2-like*) by the non-marine diets. Among the genes putatively associated with apoptotic processes, *serine/threonine-protein kinase ULK4* and *growth arrest and DNA-damage-inducible protein GADD45 beta* showed a trend towards up-regulation by ABP diet.

Regarding the immune-related genes (Table 5), five of those classified as antibacterial were down-regulated by non-marine diets. However, four features identified as immunoglobulins, also classified as antibacterial, were up-regulated by ABP diet. The two putative antiviral genes identified in the microarray experiment (i.e., *mxb* and *ifit5*) presented up-regulated transcript levels in fish fed VEG diet. The transcript levels of the genes putatively related to other immune pathways showed trends similar to either that of the antibacterial (e.g., *alpha-1-acid glycoprotein 1*, *glucosidase 2*), the immunoglobulins (e.g., *dedicator of cytokinesis protein 11*), or the antiviral genes (e.g., *major histocompatibility complex class I*). Of the four features annotated as *major histocompatibility complex class I* (*mhcI*), one showed an opposite trend, showing down-regulated transcript levels in the VEG group (probe ID C027R162). Given that the probe was designed based on a *Salmo trutta* cDNA sequence (best BLASTn hit is a *Salmo trutta mhcI* UBA allele; GenBank accession number AM262751) and the highly polymorphic nature of the *mhcI* genes, it is difficult to assert if observed expression discrepancies are due to differential paralogue regulation or to the specificity of the microarray probe.

The microarray features identified as transcripts involved in oxidation-reduction processes (Table 6) displayed contrasted expression patterns. Some transcripts encoding oxidizing enzymes showed up-regulation in salmon fed ABP diet compared with MAR or VEG diets (e.g., *cytochrome oxidase subunit I*, *glycine dehydrogenase*). By contrast, others were downregulated by ABP diet compared with MAR or VEG diets (e.g., *glutathione S-transferase theta-2B*). On the other hand, the expression levels of *cytochrome P450 1A1* and other transcripts were significantly reduced by VEG diet compared with MAR diet.

### qPCR validation

**Fig. 1:**
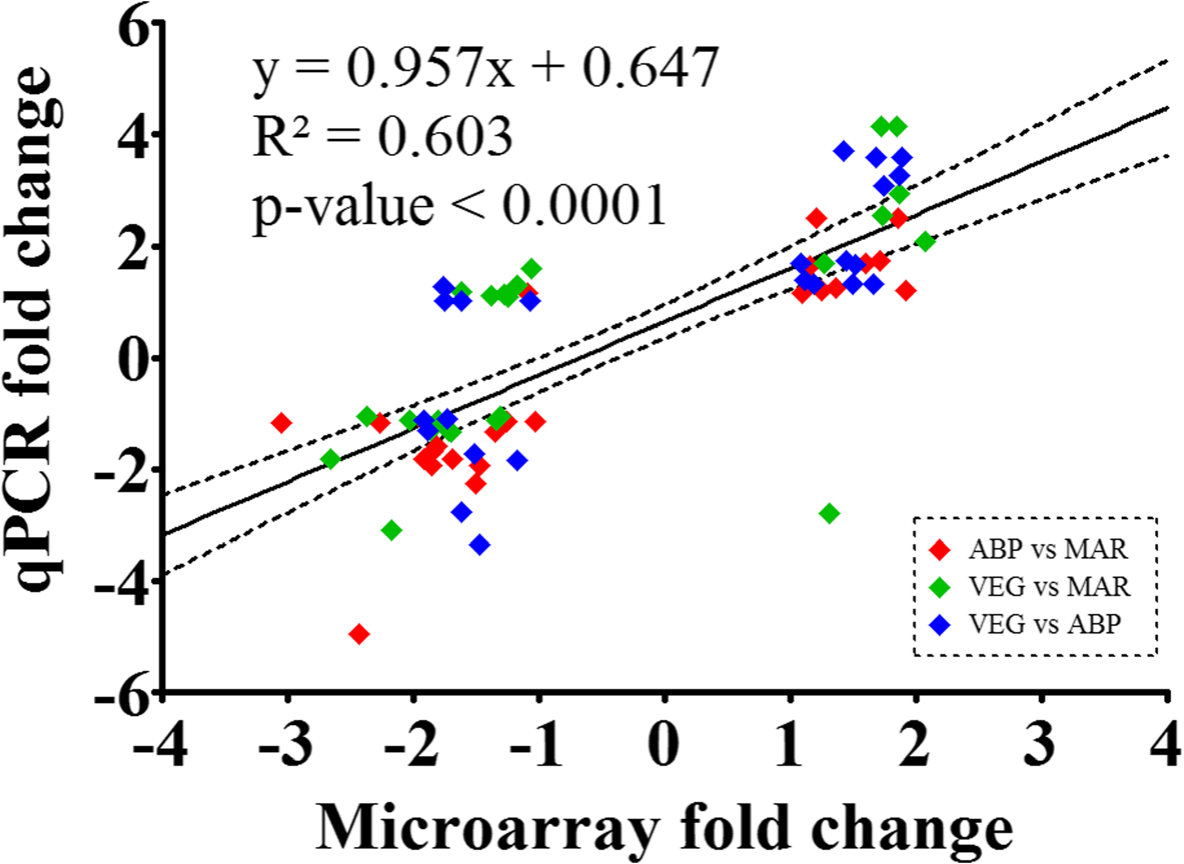
Scatter plot of gene expression fold-changes between diets calculated from the microarray log_2_ ratios and log_2_-transformed qPCR relative quantity (RQ) ratios. Each dot represents either an ABP vs MAR, VEG vs MAR, or VEG vs ABP comparison for a given gene

Fold-change values (FCs) calculated from microarray log_2_ ratios, and log_2_-transformed qPCR RQ ratios, showed a significant correlation (Fig. 1). The main deviation from linearity consisted of a group of dots corresponding to microarray-derived FCs between − 2 and − 1 and qPCR-derived FCs between 1 and 2. A closer look at these FCs revealed that most of them pertained to VEG vs MAR (six out of 12) and VEG vs ABP (five out of 12) comparisons. None of the genes was especially represented among those FCs. The sum of the potential divergences between microarray and qPCR results (see Discussion), and the inclusion of more biological replicates in the qPCR study, would likely be behind this deviation.

**Fig. 2:**
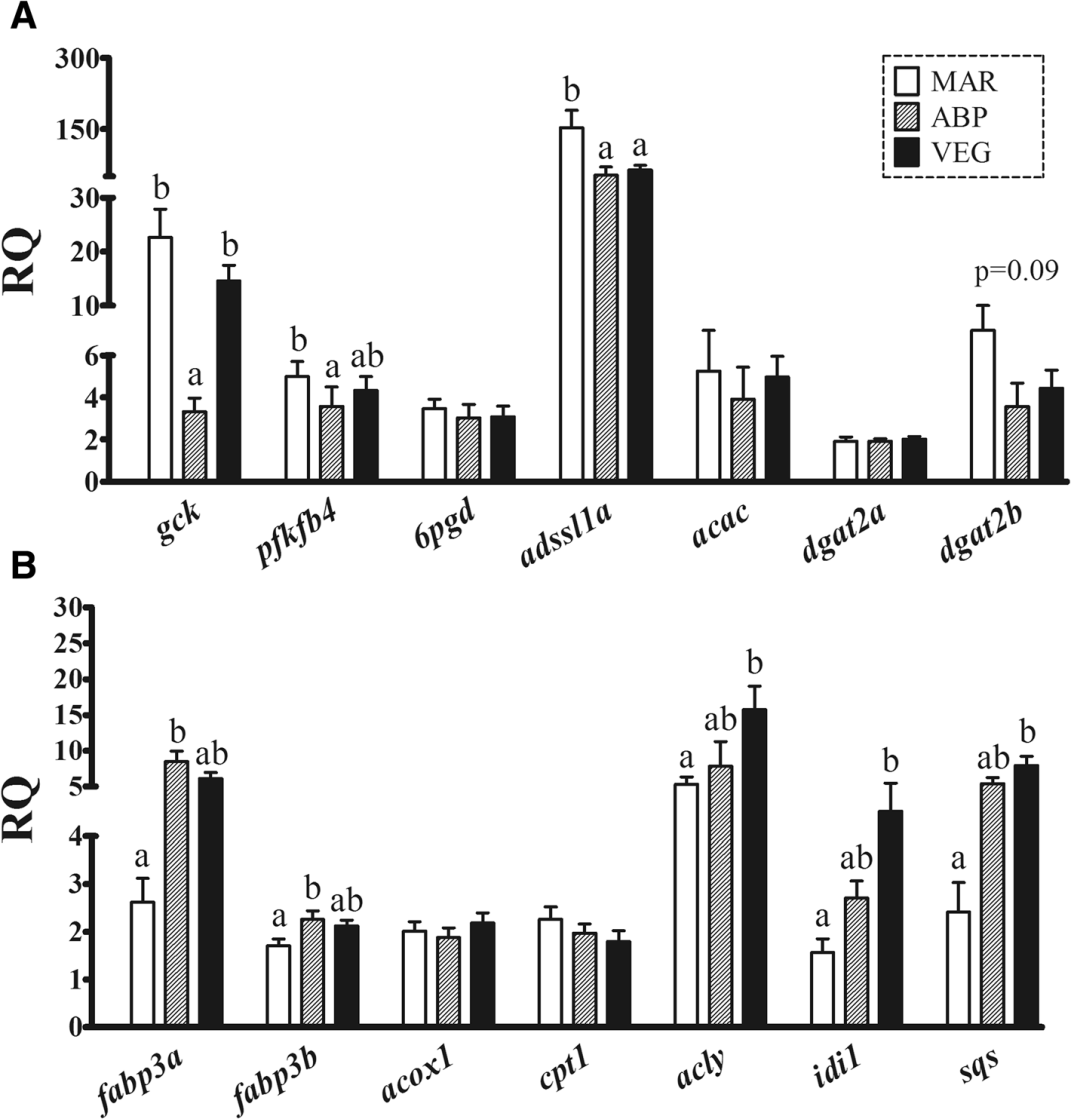
Results from the qPCR analysis of selected transcripts involved in carbohydrate (A: *gck*, *pfkfb4*, *6pgd*), nucleotide (**A**: *adssl1a*), and lipid metabolism (**A**: *acac*, *dgat2a*, *dgat2b*; **B**: *fabp3a*, *fabp3b*, *acox1*, *cpt1*, *acly*, *idi1*, *sqs*). Columns and error bars represent mean relative quantity (RQ) values and S.E., respectively. Different letters above error bars represent significant differences between diets [one-way ANOVA, Tukey’s (homogeneity of variances among groups) or Games-Howell (variances not homogeneous across groups) post-hoc test, *p* < 0.05]. Gene abbreviations (below their corresponding columns) are according to UniProt terminology

The qPCR results showed that feeding ABP diet resulted in significantly lower transcript levels of *gck* and *pfkfb4* compared with MAR diet (Fig. 2A). In addition, the qPCR analysis showed a significant down-regulation of *gck* transcription by ABP diet compared with VEG diet. No significant differences among dietary treatments were found in the transcription of *6pgd* and *acac* (Fig. 2A). Nevertheless, *adssl1a* transcription was significantly down-regulated by non-marine diets (Fig. 2A).

The qPCR experiment did not find significant changes in *dgat2a* transcription among dietary groups (Fig. 2A). However, *dgat2b* showed a trend towards lower transcript levels in the liver of salmon fed non-marine diets (Fig. 2A; *p* = 0.09). The qPCR analysis also showed that ABP diet significantly up-regulated the transcript levels of *fabp3a* and *fabp3b* compared with MAR diet (Fig. 2B). Neither *acox1*, nor *cpt1* were found to be significantly modulated by the diets (Fig. 2B). In contrast, the qPCR analysis found up-regulated *acly*, *idi1* and *sqs* hepatic mRNA levels in salmon fed VEG diet compared with those fed MAR diet (Fig. 2B).

**Fig. 3:**
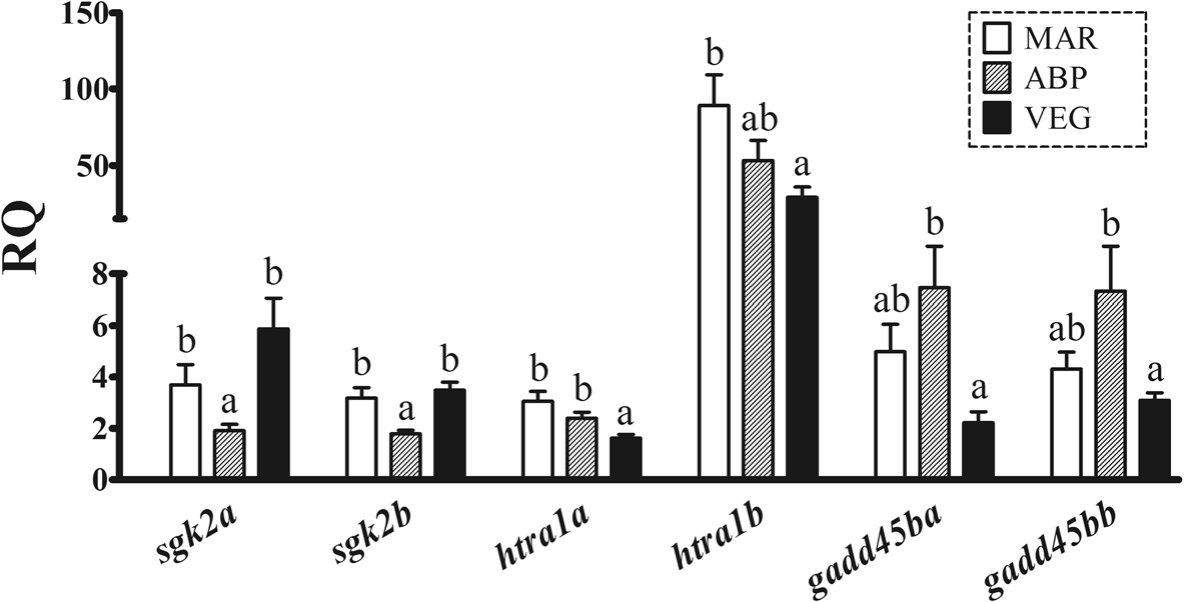
Results from the qPCR analysis of selected transcripts involved in cell growth and proliferation (*sgk2a*, *sgk2b*, *htra1a*, *htra1b*), and apoptosis (*gadd45ba*, *gadd45bb*). Columns and error bars represent mean relative quantity (RQ) values and S.E., respectively. Different letters above error bars represent significant differences between diets [one-way ANOVA, Tukey’s (homogeneity of variances among groups) or Games-Howell (variances not homogeneous across groups) post-hoc test, *p* < 0.05]. Gene abbreviations (below their corresponding columns) are according to UniProt terminology

The qPCR analysis of *sgk2a* and *sgk2b* mRNA levels confirmed their down-regulation by ABP diet compared with MAR and VEG diets (Fig. 3). Both *htra1* paralogues were down-regulated by VEG diet as compared with ABP (only *htra1a*) and MAR (both). Similarly, *gadd45ba* and *gadd45bb* transcript levels were down-regulated by VEG diet compared with ABP diet.

**Fig. 4:**
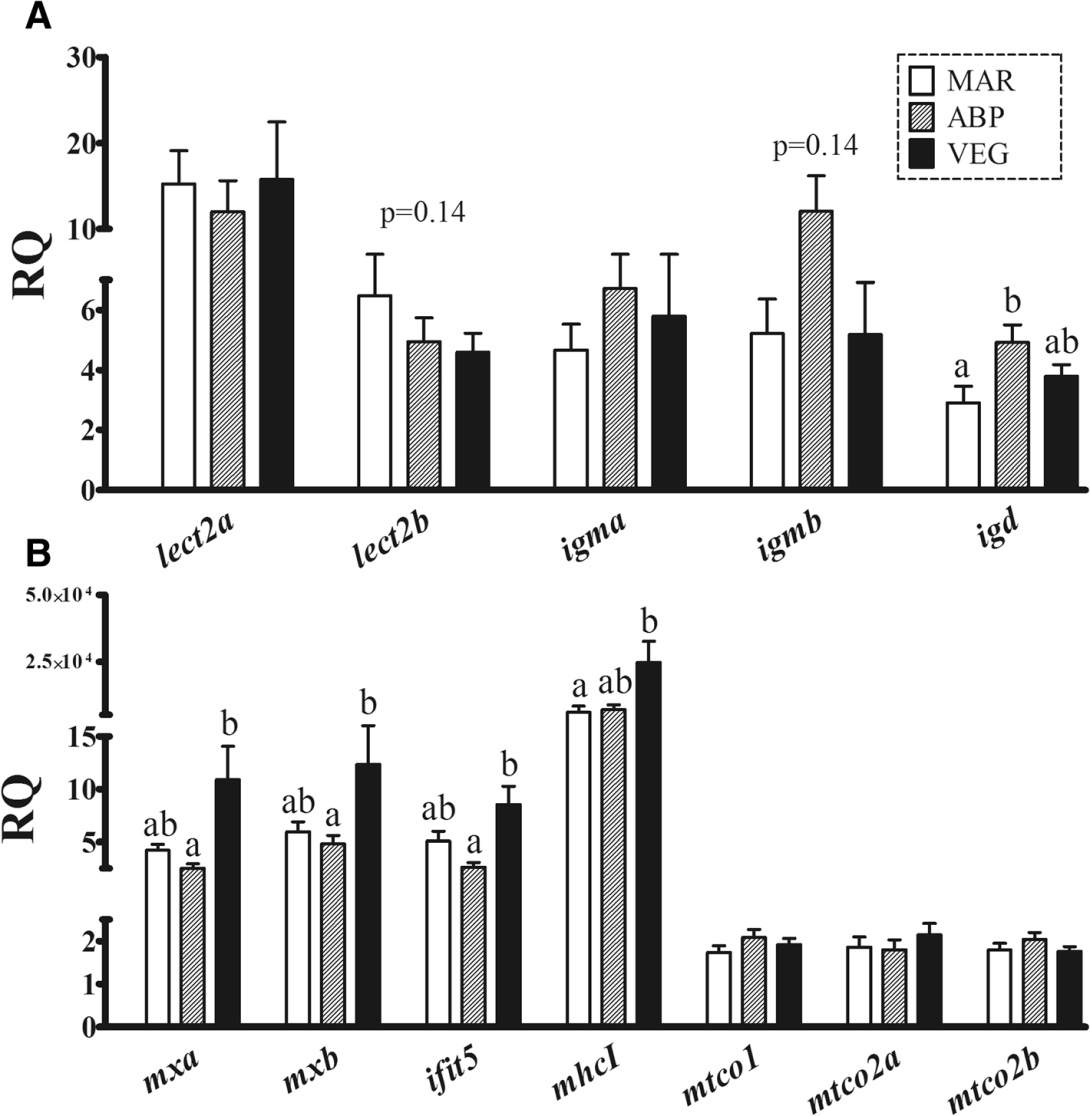
Results from the qPCR analysis of selected antibacterial (**A**: *lect2a*, *lect2b*, *igma*, *igmb*, *igd*), antiviral (**B**: *mxa*, *mxb*, *ifit5*), and other immune-related (**A**: *mhcI*) transcripts, as well as transcripts involved in oxidation-reduction processes (**A**: *mtco1*, *mtco2a*, *mtco2b*). Columns and error bars represent mean relative quantity (RQ) values and S.E., respectively. Different letters above error bars represent significant differences between diets [one-way ANOVA, Tukey’s (homogeneity of variances among groups) or Games-Howell (variances not homogenous across groups) post-hoc test, *p* < 0.05]. Gene abbreviations (below their corresponding columns) are according to UniProt terminology

According to the qPCR results, *lect2a* transcription was not responsive to diet (Fig. 4A). Although not statistically significant, *lect2b* mRNA levels seemed to be down-regulated by the non-marine diets (Fig. 4A; *p* = 0.14). *Igma*, *igmb*, and *igd* showed a similar trend towards up-regulation by ABP, which was only statistically significant for *igd* as compared with the results of MAR-fed salmon (Fig. 4A). The expression of the antiviral transcripts *mxa*, *mxb*, and *ifit5* was significantly increased by VEG diet compared with ABP diet (Fig. 4B). VEG diet also up-regulated *mhcI* transcription as compared with salmon fed MAR diet (Fig. 4B). The transcripts representative of the oxidation-reduction processes in the qPCR experiment, *mtco1*, *mtco2a*, and *mtco2b*, did not show significant expression differences among dietary groups (Fig. 4B).

For more details on DNA sequence identity between paralogues, and between genes and 60-mer microarray probes and their related contigs, see Additional file 2. For supporting information on paralogue-specific primer design, see Additional files 3, 4, 5, 6, 7, 8, 9, 10, 11 and 12.

### Correlations between gene expression and liver fatty acids

**Fig. 5:**
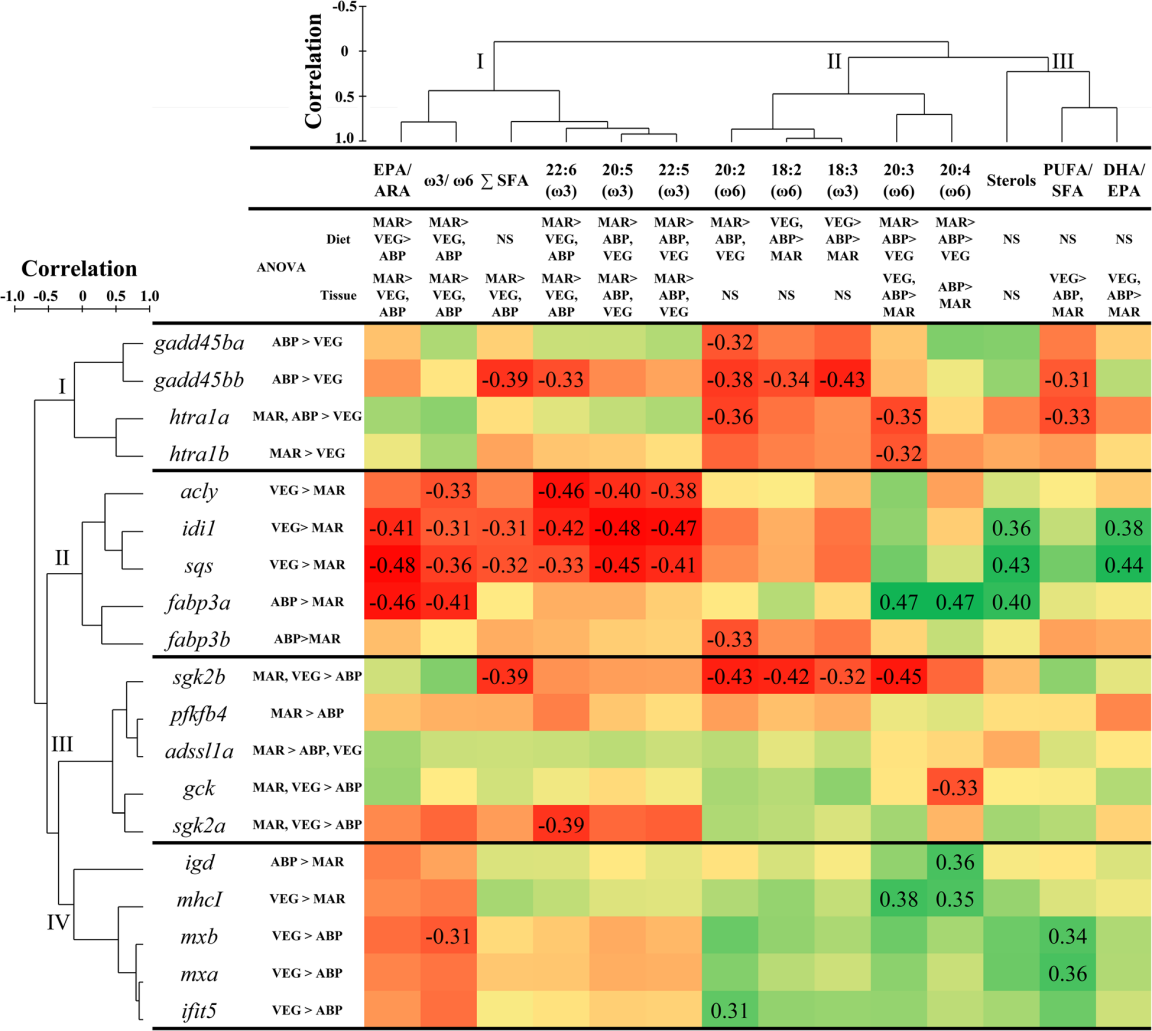
Matrix representing Pearson’s correlation coefficients between log_2_-transformed RQs of the qPCR-analyzed transcripts (rows) and the log_2_-transformed levels of different lipid composition parameters (columns) in the liver of salmon fed the experimental diets. Significant differences among diets (one-way ANOVA, *p* < 0.05) are indicated on the right of the transcript abbreviations and below the phenotypic variables. Transcriptomic and phenotypic data were arranged based on a hierarchical clustering performed using Pearson correlation resemblance matrices (PRIMER, Version 6.1.15, Ivybridge, UK). Dendrograms next to transcript abbreviations and above lipid parameters represent the results of the hierarchical clustering analyses. EPA: eicosapentaenoic acid (20:5ω3); ARA: arachidonic acid (20:4ω6); SFA: saturated fatty acids; PUFA: polyunsaturated fatty acid; DHA: docosahexaenoic acid (22:6ω3)

Hierarchical clustering segregated the qPCR-validated genes of interest (GOIs) into four groups (Fig. 5). *Gadd45ba*, *gadd45bb*, *htra1a,* and *htra1b* constituted the first cluster and were all repressed in fish fed diet VEG. The second cluster comprised all those genes related to lipid metabolism, and thus, they are referred to as ‘lipid metabolism-related’ genes. Genes directly or indirectly involved in the metabolism of carbohydrates were grouped in Cluster III and referred to as ‘carbohydrate metabolism-related’ genes. All immune-related genes were grouped in Cluster IV.

Liver FA levels and ratios were segregated into three major groups by hierarchical clustering (Fig. 5). The ω3 LC-PUFAs EPA (20:5ω3), 22:5ω3, and DHA (22:6ω3), together with EPA/ARA, the ω3/ω6 ratio, and the sum of saturated FAs (∑SFA) constituted Cluster I. All these parameters showed significantly higher values in the liver of salmon fed MAR diet. In contrast, omega-6 (ω6) LC-PUFAs (i.e., 20:2ω6, linoleic acid [18:2ω6], 20:3ω6, and ARA [20:4ω6]) and α-linolenic acid (18:3ω3) in Cluster II were present at lower levels in the liver of fish fed MAR diet, although differences were only statistically significant for 20:3ω6 (for MAR vs ABP and VEG) and ARA (for MAR vs ABP). Hepatic sterol levels and PUFA/SFA, and DHA/EPA ratios were grouped in Cluster III as values tended to be higher in salmon fed VEG diet, and lower in those fed MAR diet. This trend was statistically significant for hepatic PUFA/SFA and DHA/EPA ratios, but not for sterol levels.

The transcription of *gadd45ba*, *gadd45bb*, and *htra1a* showed negative correlation with 20:2ω6 (Fig. 5). Also, *htra1a* and *htra1b* were inversely proportional to liver 20:3ω6 levels. The hepatic mRNA levels of *acly*, *idi1*, *sqs,* and *fabp3a* correlated negatively with ω3/ω6. The transcription of *acly*, *idi1*, and *sqs* and the hepatic levels of EPA, 22:5ω3, and DHA were also negatively correlated. Separately, *idi1,* and *sqs* transcript levels correlated negatively with ∑SFA, and positively with DHA/EPA. *Idi1*, *sqs*, and *fabp3a* showed negative correlation with EPA/ARA and positive correlation with sterols. Among the carbohydrate metabolism-related genes, *sgk2b* showed transcript levels that correlated negatively with ∑SFA, 20:2ω6, 18:2ω6, 18:3ω3, and 20:3ω6, whereas those of *sgk2a* were inversely correlated with DHA. *Gck* transcript levels correlated negatively with hepatic ARA levels. Overall, the transcription of the immune-related genes (i.e., *igd*, *mhcI*, *mxb*, *mxa*, and *ifit5*) seemed to correlate negatively with FAs levels and ratios of Cluster I, and positively with those of Clusters II and III. However, the trends above were significant only in some cases. The mRNA levels of *igd* and *mhcI* were significantly positively correlated with ARA hepatic levels. The transcript levels of both *mx* paralogues showed significant positive correlation with PUFA/SFA, and those of *mxb* correlated negatively with liver ω3/ω6. The liver levels of *ifit5* transcripts and 20:2ω6 were significantly and positively correlated.

### Correlations between gene expression and muscle fatty acids

**Fig. 6:**
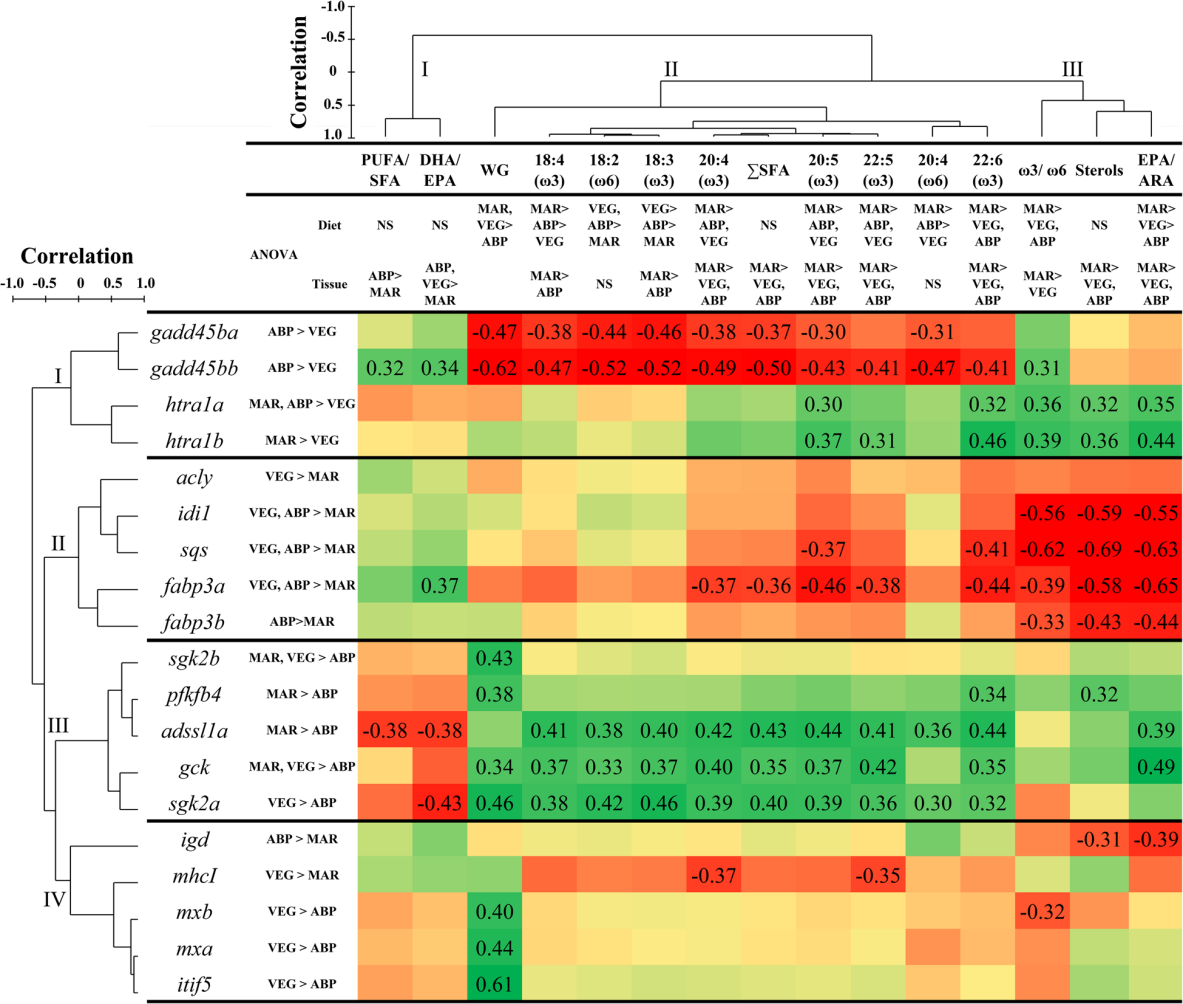
Matrix representing Pearson’s correlation coefficients between log_2_-transformed RQs of hepatic transcripts (rows) and phenotypic features such as omega-3 and omega-6 FAs levels in muscle and weight gains (columns) of the salmon fed the experimental diets. Significant differences among diets (one-way ANOVA, *p* < 0.05) are indicated on the right of the transcript abbreviations and below the phenotypic variables. Transcriptomic and phenotypic data were arranged based on a hierarchical clustering performed using Pearson correlation resemblance matrices (PRIMER, Version 6.1.15, Ivybridge, UK). Dendrograms next to transcript abbreviations and above phenotypic parameters represent the results of the hierarchical clustering analysis. PUFA: polyunsaturated fatty acid; SFA: saturated fatty acids; DHA: docosahexaenoic acid (22:6ω3); EPA: eicosapentaenoic acid (20:5ω3); WG: weight gain; ARA: arachidonic acid (20:4ω6)

Three major groups could be identified after the hierarchical clustering of muscle FA levels and ratios (Fig. 6). Cluster I is comprised of PUFA/SFA and DHA/EPA ratios, both of them showing higher values in the muscle of salmon fed ABP diet compared with those fed MAR diet. All individual FAs included in the analysis and ∑SFA were grouped in Cluster II, and all showed a trend towards higher values with MAR diet than with ABP diet, a trend that was significant in all cases but for 18:2ω6 and ARA. Similarly, muscle ω3/ω6 and EPA/ARA ratios, and sterol levels in Cluster III were increased with MAR diet compared with VEG diet, and compared to ABP diet in the case of sterols and EPA/ARA.

The expression of *gadd45b* and *gadd45b* correlated negatively with most FA parameters in Cluster II (all except 22:5ω3 and DHA for *gadd45ba*; Fig. 6). In contrast, *htra1a* and *htra1b* correlated positively with muscle EPA and DHA levels in Cluster II, as well as with muscle FA parameters in Cluster III. The transcription of the lipid metabolism-related genes (i.e., *idi1*, *sqs*, *fabp3a*, and *fabp3b*) in the liver of salmon correlated negatively with muscle parameters in Cluster III. Also, *fabp3a* transcript levels correlated negatively with many of the ω3 FAs (i.e., 20:4ω3, EPA, 22:5ω3, and DHA) and ∑SFA in Cluster II, and positively with DHA/EPA in Cluster I. Of the carbohydrate metabolism-related genes, the hepatic mRNA levels of *adssl1a*, *gck*, and *sgk2a* correlated positively with all muscle FA parameters in Cluster II, except for ARA, which showed significant correlations only with *adssl1a* and *sgk2a.* Regarding the connection between immune-related genes and muscle FA composition: hepatic *igd* transcript levels correlated negatively with muscle sterol and EPA/ARA; those of *mhcI* showed negative correlation with 20:4ω3 and 22:5ω3; and *mxb* transcription was inversely correlated with ω3/ω6. Interestingly, weight gain (WG) values correlated positively with several carbohydrate metabolism-related (i.e., *sgk2b*, *pfkfb4*, *gck*, *sgk2a*) and immune-related genes (i.e., *mxa*, *mxb*, *ifit5*), and negatively with the *gadd45b* paralogues.

### Correlation between apparent feed intake, weight gain, and gene expression

As indicated before, differences among dietary groups in apparent feed intake (AFI) were not statistically significant (*p* = 0.069, Table 2). However, AFI showed to be highly and positively correlated with salmon weight gain (*r* = 0.94, *p* < 0.0001; Additional file 13). In addition, AFI was negatively correlated with the transcript levels of *igd* (*r* = − 0.71, *p* = 0.009) and *fabp3a* (*r* = − 0.65, *p* = 0.022). Conversely, tank AFI values correlated positively with WG (r = 0.94, p < 0.0001), and the levels of the carbohydrate metabolism-related transcripts *sgk2b* (*r* = 0.65, *p* = 0.022) and *gck* (*r* = 0.74, *p* = 0.006), as well as with those of the immune-related transcripts *mxa* (*r* = 0.59, *p* = 0.044) and *ifit5* (r = 0.65, 0.022).

### Gene biomarker identification by discriminant analysis

The stepwise discriminant analysis of the qPCR data generated two functions capable of classifying fish individuals among dietary groups with a 90% accuracy (cross-validated, Table 7). The diet-discriminating functions were constructed using expression data of *sqs*, *gck*, *gadd45ba*, *sgk2b*, and *fabp3a*. On the other hand, the analysis produced a function capable of discriminating salmon with high or low growth performance with a 94% accuracy. The growth-discriminating function included *sgk2b*, *gck*, *mxa*, and *fabp3b*. Another function to discriminate fish with high or low muscle EPA + DHA levels was obtained using *fabp3a* and *igd* transcript levels. This latter function was less accurate (67%) than the diet- and growth-discriminating functions, but had a significant discriminant ability (χ^2^, *p* < 0.05).

**Table 7:** Results obtained from the stepwise discriminant analysis performed on log_2_-transformed qPCR-analyzed transcripts

Grouping variable	Biomarkers included	Function	Eigenvalue^a^	Canonical correlation^b^	Wilks’ Lambda^c^				Accuracy (%)^d^
					λ	χ^2^	df	*p*-value	
Diet (MAR, ABP, VEG)	*sqs, gck, gadd45ba, sgk2b, fabp3a*	1	4.50	0.904	0.075	67.2	10	< 0.001	90
		2	1.41	0.765	0.415	22.9	4	< 0.001	
Growth (High, Low)	*sgk2b, gck, mxa, fabp3b*	1	2.02	0.818	0.331	29.9	4	< 0.001	94
EPA + DHA (High, Low)	*fabp3a, igd*	1	0.394	0.532	0.717	7.98	2	0.019	67

^a^Ratio between sum of squares among groups and within groups calculated from the discriminant scores of each function. Strong discriminant functions present eigenvalues above one

^b^Measure of the association between the groups in the dependent variable (i.e., diet, growth, and EPA + DHA levels) and the discriminant function

^c^Wilk’s lambda is used to test the significance of the discriminant functions

^d^Accuracy of the function(s) in predicting an individual’s membership to its corresponding phenotypic class

## Discussion

Replacing FM and FO with terrestrial alternative ingredients affected the liver transcriptome of Atlantic salmon. Moreover, our findings indicate that feed formulation had an impact not only on liver transcripts related to nutrient metabolism [e.g., *glucokinase* (*gck*), *squalene synthase* (*sqs*)] but also on immune-related transcripts [e.g., *interferon-induced protein with tetratricopeptide repeats 5* (*ifit5*)]. Changes in the liver transcriptome with replacement of FM by plant protein sources have been reported in previous studies on Atlantic salmon [63, 64] and other fish species [65]. FO replacement by vegetable oils had also been found to modulate gene expression in the liver of Atlantic salmon [23, 36, 66, 67]. As could be expected, the combination of both FM and FO replacement by plant products also results in an adaptation of the liver transcriptome of Atlantic salmon [35] and rainbow trout [68]. In the present study, the experimental diets proposed as potential substitutes to the conventional marine-based (i.e., ABP and VEG diets) were formulated with rapeseed oil as the main source of dietary lipids and different proportions of plant and animal by-product meals as the main source of dietary proteins. To date, little is known about the effect of replacing FM by animal by-products on the fish liver transcriptome. Liland et al. [69] analyzed through qPCR the effects of feeding Atlantic salmon feeds formulated with a mix of animal by-products on the transcription of a selection of metabolic and immune-related genes. They did not detect significant changes in the expression of any of the selected genes. To the best of our knowledge, ours is the first experiment conducted to explore the liver transcriptome of Atlantic salmon fed high levels of protein from animal by-products.

The transcript levels of some genes (e.g., *6pgd*, *acac*, and *igma*) showed high variability among fish individuals of the same dietary treatment, which could be a major source of mismatch between microarray and qPCR fold-changes, as the sample size was increased for the qPCR analyses. Other genes (e.g., *gck*, *sgk2a*, and *ifit5*) were less affected by inter-individual variability or were more sensitive to dietary modulation, and showed better microarray-qPCR correlation. Technical limitations of the microarray platform could also explain discrepancies between qPCR and microarray results. As discussed by Booman et al. [45], one of these limitations could be the lower specificity of the microarray probe compared with that of the qPCR primers. This could be the case for those microarray features designed using cDNA sequences from other salmonid species, such as those targeting *mtco1*, and *mtco2* transcripts. Another of the limitations listed by Booman et al. [45] is that the microarray probe and qPCR primers may target different regions of the mRNA, which occurs with most of the qPCR-analyzed genes in the present study (see Additional files 3, 4, 5, 6, 7, 8, 9, 10, 11, and 12). On this point, it should be noted that paralogue-specific analyses restrict the suitable nucleotide regions for primer design (see Methods). Finally, the possibility of contig misassembly should be considered as a potential source of conflict between microarray and qPCR results [45].

### Effects of terrestrial ingredients on liver lipid metabolism: promoted cholesterol biosynthesis

The transcription of genes involved in the metabolism of carbohydrates and lipids in the liver was modulated significantly, thus showing adaptation of the hepatic molecular machinery to the different nutritional conditions. Further, the nutritional composition of the feeds based on terrestrial products appeared to be metabolically challenging, especially for lipid metabolism. Numerous studies have shown cholesterol biosynthesis in fish is affected by dietary replacement of marine ingredients by vegetable alternatives [36, 67, 70–74]. In our study, the transcripts involved in the synthesis of cholesterol (i.e., *idi1* and *sqs*) were significantly up-regulated by VEG diet. A moderate increase in their transcription was also observed in fish fed ABP diet, but it was only statistically significant in the microarray experiment. Up-regulation of *idi1* transcription in the liver of Atlantic salmon fed vegetable oils was previously reported [70]. Cholesterol is the main sterol in animal fats and oils [75]. In contrast, in vegetable oils like the rapeseed oil of the non-marine diets ABP and VEG, sterols consist mainly of phytosterols, which are known to impede normal cholesterol absorption through the intestine of fish and mammals [75–77]. In response to this impaired absorption, the hepatic metabolism of Atlantic salmon promotes the biosynthesis of cholesterol [67, 78]. Higher hepatic *idi1* and *sqs* transcript levels in salmon fed ABP and VEG diets would agree with the higher inclusion of rapeseed oil and thereby higher phytosterol levels in those diets. Other anti-nutrients present in plant meals (e.g., oligosaccharides and saponins) can reduce bile acid reabsorption by the intestine by inducing enteritis, increasing intestinal content viscosity, or by directly binding or trapping bile acids [11, 67, 79]. Decreased intestinal bile acid reabsorption drives an increased de novo synthesis in the liver, that requires cholesterol molecules as a precursor, thus increasing the metabolic demand for cholesterol [80]. No transcripts related to bile acid synthesis were detected in the present microarray experiment. In this regard, it should be noted that the vegetable protein sources chosen for our experimental diets were soy protein concentrate and glutens from wheat and corn, which contain low levels of anti-nutrients compared with other plant meals.

The up-regulation of cholesterol biosynthesis would contribute to maintaining cholesterol homeostasis in salmon fed non-marine diets. At the end of the trial, sterol concentrations in the liver of Atlantic salmon were similar among dietary treatments. Furthermore, liver sterol concentrations were positively correlated with *idi1* and *sqs* mRNA levels. In contrast, the muscle of fish fed ABP and VEG diets had sterol levels ~ 4 times lower than those of fish fed MAR diet [42]. Consequently, muscle sterol levels and *idi1* and *sqs* transcript levels were negatively correlated. As the liver is the main organ for the regulation of cholesterol homeostasis, with a faster turnover than skeletal muscle [77], it is not surprising that fish fed the non-marine diets could metabolically maintain liver but not muscle sterols levels. The question arises as to what pathway supplied the acetyl-CoA required for an up-regulated synthesis of cholesterol. One possible source could be the β-oxidation of fatty acids [81]. Up-regulated *fabp3a* and *fabp3b* transcription in the liver of fish fed ABP diet – also in fish fed VEG diet, although to a smaller extent – seems to support this hypothesis, as FABP3 transports fatty acids from cell membranes to mitochondria for β-oxidation [82]. In line with this, the microarray analysis identified the gene encoding adiponectin, a protein widely accepted to stimulate FA β-oxidation in mammals [83], as up-regulated by ABP and VEG diets. Moreover, *idi1* and *sqs* transcript levels correlated negatively with the liver ∑SFA levels, which were reduced in salmon fed the non-marine diets, even though all diets presented similar SFA contents. However, and despite all this evidence, genes directly involved in mitochondrial (*cpt1*) and the peroxisomal (*acox1*) β-oxidation were not induced by the increased acetyl-CoA demand. On the other hand, acetyl-CoA can also be synthesized from citrate by ACLY [84], a cytosolic enzyme that showed to be up-regulated at the mRNA level by VEG diet. The precursor citrate derives from the mitochondrial TCA cycle, making of ACLY a connection between the catabolism of glucose, FAs, and amino acids, and the synthesis of cholesterol [84, 85]. In other words, the source of carbon skeletons for the increased cholesterol synthesis rates remains unclear, since *acly* dietary modulation differed from that of the glycolysis-related transcripts (i.e., *gck* and *pfkfb4*), and there is no clear trend among the transcripts involved in fatty acid β-oxidation. Contradictory findings on the effects of plant feedstuffs on the expression of transcripts related to β-oxidation can be found in the Atlantic salmon literature [35, 36, 70, 82, 83]. Also, the post-translational regulation of these pathways should be considered as suggested by previous research in mammals [85, 86]. In sum, further research is needed on the molecular mechanisms controlling acetyl-CoA availability for cholesterol synthesis in the liver of farmed Atlantic salmon.

Besides acetyl-CoA, SQS and other enzymes involved in the anabolism of cholesterol would also need NADPH as cofactors. Most of the NADPH in the cytosol is supplied by the oxidative branch of the pentose phosphate pathway (PPP) [87]. Glucose-6-phosphate dehydrogenase (G6PD) and 6PGD mediate in this pathway that converts glucose-6-phosphate into ribulose-5-phosphate, producing two molecules of NADPH. Although identified in the microarray experiment, the qPCR analyses could not detect significant differences in *6pgd* transcription among diets. Moreover, all transcriptomic evidence leaned more towards a slight downregulation of *6pgd* in fish fed ABP and VEG diets. Other NADPH-producing pathways such as malic enzyme could help cover the increased demand for NADPH in fish fed the non-marine diets [70], but none of them was detected in the microarray experiment. Instead, a more likely explanation could be found in the modulation of other metabolic-related genes. Our microarray and qPCR data suggest a decrease in the expression of genes related to the synthesis of triacylglycerols (e.g., *dgat2b*). Furthermore, fatty acid synthesis also appears to be down-regulated by non-marine diets, according to the microarray data (e.g., *acac*, *fatty acid hydroxylase domain-containing protein 2*, *fatty acid synthase*), although it could not be confirmed by the qPCR analysis of *acac* mRNA levels. Just as for cholesterol, synthesis of fatty acids and triacylglycerols requires NADPH. Therefore, increased cholesterol synthesis in fish fed ABP and VEG diets was possibly enabled by decreasing the synthesis of other lipid species.

### Effect of feed intake and dietary carbohydrate levels on liver metabolism

Atlantic salmon fed ABP showed down-regulated hepatic glycolysis as indicated by lower transcript levels of *gck* and *pfkfb4*. GCK catalyzes the phosphorylation of glucose to glucose-6-phosphate in the liver of fish and other vertebrates [88]. Similar to mammals, the mRNA and activity levels of glucokinase in the liver of fish respond to the proportion of carbohydrates in the diet [89]. ABP diet had a lower proportion of raw wheat (Table 1) – the main source of carbohydrates in our experimental diets – than MAR diet. However, so did VEG diet and salmon under this dietary treatment also showed up-regulated *gck* transcription compared with ABP diet. Instead, the significant positive correlation between *gck* transcript levels and AFI suggests that feed intake could have been the main driver in the *gck* transcriptional regulation. Glucokinase gene expression and activity were previously found to adapt to feed ration size in gilthead seabream [58]. In mammals and fish [89], the bifunctional enzyme PFKFB controls the cytosolic concentration of fructose 2, 6-bisphosphate, a metabolite that allosterically regulates the activity of the enzymes phosphofructokinase-1 (glycolysis) and fructose-1, 6-biphosphatase (gluconeogenesis). The transcription of *pfkfb* has also been reported to increase with carbohydrate levels in the diet and feed ration [59]. The fact that *pfkfb4* transcript levels did not correlate with AFI values and were a bit lower, although not significantly, with VEG diet could be due to an interaction of both factors: dietary carbohydrate levels, and feed intake.

GCK activity supplies the oxidative branch of the PPP with glucose-6-phosphate molecules, the oxidation of which generates NADPH for anabolic processes. 6PGD mediates in the pathway and was microarray-detected in our study as down-regulated by VEG diet. However, the qPCR analysis could only verify that *6pgd* transcription was not up-regulated in response to the higher NADPH demand for cholesterol synthesis in salmon fed ABP and VEG diets. Thus, we infer that lower carbohydrate intake due to lower AFI (Table 2) or dietary carbohydrate proportion might have restricted the PPP. As discussed before, a limited production by PPP could have driven liver cells to divert NADPH from other anabolic pathways towards cholesterol synthesis. The influence of feed intake and dietary carbohydrates on hepatic cholesterol synthesis in Atlantic salmon warrants further investigation. Further downstream of the pathway, in the non-oxidative branch of the PPP, ribulose-5-phosphate originating from the oxidation of glucose-6-phosphate is metabolized into ribose-5-phosphate, which serves as a substrate for de novo synthesis of nucleotides [87]. In our study, nucleotide synthesis in the liver of salmon was likely down-regulated by ABP and VEG diets, as suggested by the microarray experiment, and confirmed by the qPCR analysis of *adssl1a* transcripts. Mammals possess two ADSS isozymes: a basic form (ADSS1) involved in the purine nucleotide cycle, and an acidic form (ADSS2) that catalyzes the de novo synthesis of adenosine monophosphate [90, 91]. ADSS proteins and genes have not been molecularly characterized in fish yet, although *adssl1* has been related to cell apoptosis in zebrafish [92], and Atlantic salmon *adssl2* was found to be down-regulated by dietary glucosinolate supplementation [93]. From a nutritional perspective, it is worth investigating the role of ADSS1 in fish glucose metabolism, as recent findings suggest a regulatory function in insulin secretion by mammalian β cells [94]. Interestingly, transcript levels of *adssl1a* correlated well with other hierarchically clustered transcripts (the ‘carbohydrate metabolism-related’ genes, especially *pfkfb4*). Taken together, differences in carbohydrate intake among dietary treatments seemed to have affected not only liver glucose metabolism but also other pathways that branch off from its intermediaries.

Based on their transcription patterns, two genes encoding SGK2a and SGK2b were also grouped within the ‘carbohydrate metabolism-related’ genes cluster in Figs. 5 and 6. SGKs are a family of serine/threonine kinases implicated in the regulation of ion transport, proliferation, apoptosis, and differentiation of mammalian cells [95], and therefore *sgk2a* and *sgk2b* were initially classified as related to “cell growth and proliferation” (Table 4). Three SGK proteins have been identified in mammals, and SGK2 was found to be the predominant form in the liver [96]. SGKs have been poorly studied in fish, and the little information available is limited to SGK1 [97, 98]. In the human hepatoma cell line HepG2, Gotoh and Negishi [99] found SGK2 dephosphorylation to contribute to the activation of drug-induced gluconeogenesis. Interestingly, these authors’ findings in mouse liver implied the existence of a different pathway by which SGK2 dephosphorylation would inactivate gluconeogenesis. Therefore, the molecular function of SGK2 may vary among species. Based on our correlation analyses, *sgk2a* and *sgk2b* transcripts responded to dietary conditions in a similar fashion to the transcripts involved in the metabolism of carbohydrates. Moreover, the transcription of *sgk2b* correlated significantly with AFI. Carnivorous fish such as Atlantic salmon have a limited ability to regulate hepatic gluconeogenesis in response to carbohydrate intake, which has been proposed as a possible explanation for fish glucose intolerance [100]. After considering all the above, future studies should functionally characterize SGK2 and investigate its significance in carbohydrate metabolism in the liver of fish.

Our analyses showed a correlation between the expression of the ‘carbohydrate metabolism-related’ transcripts and certain tissue FA levels. This strengthens the idea that the interaction between lipid and carbohydrate metabolisms through NADPH availability could have influenced the FA composition of salmon tissues, especially the muscle. Feed intake would have also contributed to the regulation of hepatic lipid metabolism, as indicated by the significant correlation between *fabp3a* transcript levels and AFI values. All our experimental diets were formulated by EWOS Innovation (now Cargill Innovation) to meet salmonid requirements of essential nutrients [41]. Therefore, salmon growth can only be attributed to the balance between metabolic resources provided by diet and the metabolic cost of maintaining existing tissues. In fact, weight gain was found to be positively correlated with AFI values. Considering the latter, it is not surprising that the mRNA levels of *sgk2a*, *sgk2b*, *gck*, and *pfkfb4* correlated positively with salmon weight gain.

### Dietary modulation of apoptosis, immunity, and inflammation

The liver of fish fed VEG diet showed decreased transcription of *htra1a* and *htra1b*. Tacchi et al. [63] previously reported hepatic *htra1* transcription to be affected by feeding Atlantic salmon a diet with low FM/ high plant protein levels. In mammals, serine protease HTRA1 mediates in multiple biological processes by antagonizing IGF-binding proteins and proteins of the TGF-beta family [101]. Tacchi et al. [63] hypothesized that *htra1* up-regulation by plant proteins could be a compensatory response upon the concurrent induction of apoptotic transcripts involved in the TGF-beta pathway. In contrast to Tacchi et al. [63], *htra1a* and *htra1b* transcription was repressed by replacing dietary FM with plant proteins in the present study. However, the interaction between *htra1* and apoptosis may be inferred from the down-regulation of *gadd45b* paralogues (qPCR validated) and *serine/threonine-protein kinase ULK4* (microarray identified) by VEG diet. In mammals, GADD45B and ULK4 were related to the regulation of apoptosis through p38 MAPK signaling pathways [102, 103], pathways in cross-talk with that of TGF-beta [104]. Piscine HTRA1 would require further investigation before drawing conclusions on its significance in salmon physiology. However, should HTRA1 in fish regulate the TGF-beta signaling pathway, then it may explain the promoted transcription of some immune transcripts in the liver of fish fed VEG diet (i.e., *mxa*, *mxb*, *ifit5*, and *mhcI*). Evidence in fish suggests that TGF-beta plays immunomodulatory roles similar to those observed in mammals [105]. Interestingly, recombinant human TGF-β1 was found to induce *mhcI* in grass carp [106]; and in the liver transcriptome of Atlantic salmon fed a plant-based diet, the induction of the transcript encoding the hypothesized TGF-β1-antagonist *htra1* coincided with a repression of *mhcI* transcription [63].

Both qPCR and microarray agreed in that VEG diet up-regulated the basal expression of antiviral transcripts (i.e., *mxa*, *mxb*, and *ifit5*) and *mhcI* in the liver of salmon. In a study conducted subsequent to the present feeding trial, VEG diet was found to boost the expression of antiviral transcripts in the head kidney of Atlantic salmon 24 h after an intraperitoneal injection of the viral mimic polyriboinosinic polyribocytidylic acid (pIC) [28]. In mammals, the liver acts as a defense barrier against pathogens after they pass through the digestive tract [107]. Leukocytes in the liver of rainbow trout have been reported to represent 15–29% of non-hepatocytes [108]. In contrast, the head kidney of fish equates to the red bone marrow of higher vertebrates as a major hematopoietic and lymphoid organ [109]. Considering all the above, VEG diet appeared to fortify the constitutive immune defenses of the liver, and provide the metabolic conditions for the head kidney to mount a stronger immune response against immune challenges.

In the liver of salmon, the extent of the transcription of the antiviral genes and/or *mhcI* correlated positively with the proportion of some ω6 FAs and the PUFA/SFA ratio in the tissue. Omega-6 FAs are known to be pro-inflammatory as they are metabolized into more bioactive eicosanoids than ω3 FAs [24]. SFAs have also been proven to be pro-inflammatory in in vitro and in vivo mammalian models [110, 111]. In this regard, although neither of the *lect2* paralogues showed a significant response to diet, the microarray analysis detected C-C motif chemokine 13-like (*ccl13*) transcripts as more highly expressed in fish fed VEG diet than in those fed ABP diet. In mammals, CCL13 (alias monocyte chemoattractant protein 4) attracts monocytes, T cells and immature dendritic cells [112]. In fish, *ccl13* has been found to be highly responsive to pIC in cod macrophages [113], and to *Piscirickettsia salmonis* in the liver and head kidney of Atlantic salmon injected with the pathogen [114]. Likewise, VEG diet was found to up-regulate *ccl19b* transcription in head kidney of PBS-injected salmon compared with ABP diet [28]. Nevertheless, as VEG diet repressed the expression of head kidney transcripts involved in the synthesis of eicosanoids, the diet was not deemed pro-inflammatory [28], which would agree with its lower proportion in ARA compared with MAR and ABP diets. Therefore, the concurrence of up-regulated *ccl13* and immune-related transcripts in the liver of salmon fed VEG diet may suggest an increased homing of leukocytes not related to diet-induced inflammation.

In contrast with VEG diet, ABP diet was previously taken as pro-inflammatory since it up-regulated the expression of transcripts related to the synthesis of leukotrienes in the head kidney of Atlantic salmon [28]. Furthermore, lower EPA/ARA ratios of ABP would have promoted the synthesis of ARA-derived eicosanoids, which possess stronger pro-inflammatory activity than those synthesized from EPA [26, 115, 116]. No evidence of changes in the hepatic eicosanoid metabolism was found in the present microarray experiment. However, Atlantic salmon fed ABP diet showed a trend towards higher transcription of *igm* and *igd* in the liver. IgM+ B cells have been found to dominate rainbow trout peritoneal inflammatory response to bacteria and viruses [117, 118]. On the other hand, IgD function in salmonids is yet to be investigated. Nevertheless, research on IgD in vertebrates is gaining interest as evidence accumulates regarding its activity enhancing basophil-mediated inflammatory responses [119, 120]. Attending to diet formulation, ABP’s pro-inflammatory properties would be most likely related to its higher content in animal by-products, which contributed to the lower EPA/ARA ratio in the diet. In line with this, *igd* mRNA levels correlated positively with hepatic ARA levels and negatively with muscle EPA/ARA ratios. In addition, ABP-promoted *gadd45b* transcription (compared to VEG diet) could also be considered as further evidence of the pro-inflammatory nature of the diet. *Gadd45b* has been reported to play a role in modulating inflammation in mice [121] and be induced upon infection by *Piscirickettsia salmonis* [114], and infectious salmon anemia virus [122] in the head kidney and liver, respectively, of Atlantic salmon. Considering that liver is central to regulating systemic nutrient homeostasis [123], and may act as barrier defense against pathogens [107], we can conclude that there is a need for a better understanding of the metabolic and immune implications of chronic inflammation in the liver of Atlantic salmon.

### Identification of predictive gene biomarkers

The search for economically and environmentally superior sustainable feeds for the Atlantic salmon aquaculture industry has contributed to the development of new tools to analyze the performance of the fish under varying nutritional conditions. In the present study, we used qPCR data of hepatic diet-responsive transcripts to identify gene biomarkers capable of predicting desired salmon phenotypes. We report two discriminant functions based on the transcript levels of five genes (*sqs, fabp3a*, *gck, sgk2b, gadd45ba*) capable of classifying individual fish by dietary treatment with a 90% accuracy. These biomarkers cover the differences between MAR diet and the non-marine diets (*sqs*, *fabp3a*), and those between ABP diet and the other two (*gck*, *sgk2b*, *gadd45ba*). Another discriminant function could correctly classify 94% of the individuals by weight gain (two categories: low and high) using only four genes: *gck*, *sgk2b*, *fabp3b*, *mxa*. Understandably, all four genes were characteristically down-regulated by ABP diet in comparison with MAR and VEG diets. A third discriminant function with *fabp3a* and *igd* as predictors allowed the prediction of EPA + DHA levels in the muscle of Atlantic salmon with a 67% accuracy. As explained above, hepatic *fabp3a* mRNA levels showed a negative correlation with muscle EPA and DHA concentrations. What is not so evident is the connection between the transcription of *igd* and that of genes related to lipid metabolism. To our knowledge, no molecular mechanisms co-regulating the transcription of *igd* and lipid-metabolism genes have been reported to date. Nevertheless, as discussed above, *igd* transcript levels showed a negative correlation with muscle EPA/ARA ratios, which could explain its ability to predict EPA + DHA levels in muscle.

In the present study, we propose the stepwise discriminant analysis as a promising method for predicting the performance of farmed Atlantic salmon based on the expression of biomarker genes. This method could also be used to predict other relevant diet effects for Atlantic salmon aquaculture, such as diet-derived immune resistance against pathogens. In fact, stepwise discriminant analysis has previously been utilized and proposed for prognosis prediction of cancer and tuberculosis patients [124, 125]. Notwithstanding the accuracy of the present post hoc predictions, for these functions to be used to classify cases in a predictive manner, they will need to be cross-validated with results from other feeding trials with Atlantic salmon. Ideally, new and more robust functions could be generated by using trancriptomic data compiled from multiple studies. In this way, the influence of differential genetic backgrounds and holding conditions (e.g., water quality, photoperiod, feeding regime) could be accounted for in the predictive function. Also, the applicability of these functions will depend on the diet formulations tested in the studies from which the gene expression data is collected. For example, the growth-predicting biomarkers identified here were associated with the inclusion of animal by-products and reduced feed intake. Well-established microarray platforms like the one used here are adequate for such analyses as the assays should be standardized across studies. Furthermore, a more inexpensive alternative could be the use of multiplex qPCR panels composed by highly predictive biomarker genes selected by stepwise discriminant analysis. This opens an opportunity for the development of routine assays for the screening of different fish performance parameters (e.g., growth, stress, immune status) by the Atlantic salmon farming industry. Lastly, the stepwise discriminant analysis may become a powerful tool for the formulation of superior feeds for Atlantic salmon should the phenotype-predictive functions be applied to transcriptomic data from early time points of the feeding trials (e.g., two to 3 weeks after the beginning of the trial).

## Conclusions

We present new findings on the transcriptomic changes in the liver of Atlantic salmon fed diets with high replacement of FM and FO by terrestrial alternatives. Based on our results, the hepatic metabolic machinery of Atlantic salmon adapted to the nutritional challenges of such dietary formulations. The need for increased cholesterol biosynthesis in ABP and VEG groups seemed to be the main driver of the modulation of the hepatic metabolism and the changes in the tissue fatty acid composition. On the other hand, the poorer growth performance of fish fed ABP diet seemed to be related to lower feed intake, which was found to be correlated with the mRNA levels of the ‘carbohydrate metabolism-related’ transcripts analyzed (i.e., *gck* and *sgk2b*). We provide new evidence on the expression of *htra1a* and *htra1b* transcripts being modulated by the proportion of plant products in the diet of salmon. Concurrently, the plant-based formulation of VEG diet promoted higher mRNA levels of antiviral transcripts (i.e., *mxa*, *mxb*, and *ifit5*) and *mhcI*, as opposed to the trend observed with *htra1a* and *htra1b*. Therefore, we hypothesize a possible interaction between *htra1* paralogues and antiviral immunity in the liver of Atlantic salmon. We found no indication of a pro-inflammatory effect by VEG diet, which agrees with our previous findings in the head kidney of Atlantic salmon fed these experimental diets [26]. In contrast, ABP diet up-regulated *igd* transcription, which correlated positively with a higher abundance of ARA in the liver, thus suggesting a pro-inflammatory effect of this diet. Using stepwise discriminant analysis, we identified gene biomarkers capable to accurately predict desired phenotypes within this study such as growth and muscle EPA + DHA levels. This study could lay the foundation for future research on the relevance of cholesterol and certain genes (i.e., *sgk2* and *htra1* paralogues) in the nutrition and immune system of Atlantic salmon and other fish species. From an industry perspective, on the other hand, we propose a molecular approach to analyze diet performance that could help manufacturers reduce the proportion of ingredients from overexploited fisheries in salmon aquafeeds.

## Additional files

Additional file 1: **Table S1.** Primers used for qPCR analyses. (DOCX 21 kb)

Additional file 2: **Table S2.** Complete list of microarray probes identified as diet-responsive. The results from the gene identification analysis, functional annotation, and microarray-based fold changes between diets are presented. For the qPCR-analyzed genes, data regarding the cDNA sequences utilized for primer design (e.g., the similarity between those corresponding to different paralogues) and qPCR-based fold changes between diets are also included. AA: amino acid; bp: base pair; RQ: relative quantity. (XLS 85 kb)

Additional file 3: **Figure S1.** Alignment of nucleotide sequences corresponding to *adssl1a* and *adssl1b*. Conserved nucleotides in the aligned sequences are highlighted in blue. *Adssl1a* and *adssl1b* sequences share 93% identity over 597 aligned nucleotides. The alignment and percentage identity calculation were performed using AlignX (Vector NTI Advance 11). The nucleotide regions covered by probes C107R157 and C098R022 from the Agilent 44 K salmonid microarray (GEO accession number: GPL11299) are indicated within boxes. The forward qPCR primer for *adssl1a* is in bold and single underlined, whereas the reverse qPCR primer is in bold and double underlined. The qPCR primers designed for *adssl1b* are not included in the figure as none of them passed our quality tests. (DOCX 28 kb)

Additional file 4: **Figure S2.** Alignment of nucleotide sequences corresponding to *dgat2a* and *dgat2b*. Nucleotides conserved in all sequences are highlighted in yellow, and in blue for those nucleotides shared by three sequences from different paralogues, or by two but the nucleotide is deleted in the third (or the sequence is not long enough); finally, non-conserved nucleotides between paralogues are indicated by highlighting the nucleotides of one of them in green. “Rc” after the GenBank accession numbers stands for reverse complement. *Dgat2a* and *dgat2b* sequences share 83% identity over 2170 aligned nucleotides. The alignment and percentage identity calculation were performed using AlignX (Vector NTI Advance 11). The nucleotide regions covered by probes C103R066 and C134R089 from the Agilent 44 K salmonid microarray (GEO accession number: GPL11299) are indicated within boxes. Forward qPCR primers are in bold and single underlined, whereas reverse qPCR primers are in bold and double underlined. (DOCX 38 kb)

Additional file 5: **Figure S3.** Alignment of nucleotide sequences corresponding to *fabp3a* and *fabp3b*. Conserved nucleotides in all the aligned sequences are highlighted in yellow. *Fabp3a* and *fabp3b* sequences share 92% identity over 400 aligned nucleotides. The alignment and percentage identity calculation were performed using AlignX (Vector NTI Advance 11). The nucleotide region covered by the probe C086R144 from the Agilent 44 K salmonid microarray (GEO accession number: GPL11299) is indicated within boxes. Forward qPCR primers are in bold and single underlined, whereas reverse qPCR primers are in bold and double underlined. (DOCX 22 kb)

Additional file 6: **Figure S4.** Alignment of nucleotide sequences corresponding to *sgk2a* and *sgk2b*. Conserved nucleotides in all the aligned sequences are highlighted in yellow. *Sgk2a* and *sgk2b* sequences share 92% identity over 1390 aligned nucleotides. The alignment and percentage identity calculation were performed using AlignX (Vector NTI Advance 11). The nucleotide regions covered by the probes C050R117 and C168R030 from the Agilent 44 K salmonid microarray (GEO accession number: GPL11299) is indicated within boxes. Forward qPCR primers are in bold and single underlined, whereas reverse qPCR primers are in bold and double underlined. (DOCX 33 kb)

Additional file 7: **Figure S5.** Alignment of nucleotide sequences corresponding to *htra1a* and *htra1b*. Conserved nucleotides in all three aligned sequences are highlighted in yellow, those conserved in two are highlighted in blue. The cDNA sequence named as “htra1b_assembly” was assembled using the *Salmo salar* EST sequences DW576053, DW556574, DW539580, EG831192, and EG831191. *Htra1a* and *htra1b* sequences share 91% identity over 1117 aligned nucleotides. The alignment and percentage identity calculation were performed using AlignX (Vector NTI Advance 11). The nucleotide regions covered by the probes C265R134, C231R170 and C170R142 from the Agilent 44 K salmonid microarray (GEO accession number: GPL11299) is indicated within boxes. Forward qPCR primers are in bold and single underlined, whereas reverse qPCR primers are in bold and double underlined. (DOCX 37 kb)

Additional file 8: **Figure S6.** Alignment of nucleotide sequences corresponding to *gadd45ba* and *gadd45bb*. Conserved nucleotides in all the aligned sequences are highlighted in yellow. “Rc” after the GenBank accession numbers stands for reverse complement. *Gadd45ba* and *gadd45bb* sequences share 78% identity over 767 aligned nucleotides. The alignment and percentage identity calculation were performed using AlignX (Vector NTI Advance 11). The nucleotide regions covered by the probe C100R113 from the Agilent 44 K salmonid microarray (GEO accession number: GPL11299) is indicated within a box. Forward qPCR primers are in bold and single underlined, whereas reverse qPCR primers are in bold and double underlined. (DOCX 20 kb)

Additional file 9: **Figure S7.** Alignment of nucleotide sequences corresponding to *lect2a* and *lect2b*. Conserved nucleotides in all the aligned sequences are highlighted in yellow. *Lect2a* and *lect2b* sequences share 88% identity over 531 aligned nucleotides. The alignment and percentage identity calculation were performed using AlignX (Vector NTI Advance 11). The nucleotide regions covered by the probes C134R121, C164R142 and C159R112 from the Agilent 44 K salmonid microarray (GEO accession number: GPL11299) is indicated within boxes. Forward qPCR primers are in bold and single underlined, whereas reverse qPCR primers are in bold and double underlined. (DOCX 29 kb)

Additional file 10: **Figure S8.** Alignment of nucleotide sequences corresponding to *igma* and *igmb*. Conserved nucleotides in all the aligned sequences are highlighted in yellow. *Igma* and *igmb* sequences share 96% identity over 1548 aligned nucleotides. The alignment and percentage identity calculation were performed using AlignX (Vector NTI Advance 11). The nucleotide regions covered by the probes C134R121, C164R142 and C159R112 from the Agilent 44 K salmonid microarray (GEO accession number: GPL11299) is indicated within boxes. Forward qPCR primers are in bold and single underlined, whereas reverse qPCR primers are in bold and double underlined. (DOCX 32 kb)

Additional file 11: **Figure S9.** Alignment of nucleotide sequences corresponding to *mxa* and *mxb*. Conserved nucleotides in both aligned sequences are highlighted in yellow. *Mxa* and *mxb* sequences share 89% identity over 1955 aligned nucleotides. The alignment and percentage identity calculation were performed using AlignX (Vector NTI Advance 11). The nucleotide region covered by the probe C236R043 from the Agilent 44 K salmonid microarray (GEO accession number: GPL11299) is indicated within a box. Forward qPCR primers are in bold and single underlined, whereas reverse qPCR primers are in bold and double underlined. (DOCX 23 kb)

Additional file 12: **Figure S10.** Alignment of nucleotide sequences corresponding to two *mtco2* paralogues and the probe C060R108 from the Agilent 44 K salmonid microarray (GEO accession number: GPL11299). Conserved nucleotides in all three aligned sequences are highlighted in yellow, those conserved in two are highlighted in blue. *Mtco2a* and *mtco2b* sequences share 96% identity over 547 aligned nucleotides, and 83 and 85% identity compared with the 60mer microarray probe, respectively. The alignment and percentage identity calculation were performed using AlignX (Vector NTI Advance 11). Forward qPCR primers are in bold and single underlined, whereas reverse qPCR primers are in bold and double underlined. (DOCX 13 kb)

Additional file 13: **Figure S11.** Scatter plots showing linear correlations between log_2_ apparent feed intake (AFI) and: A) log_2_ weight gain (WG); B) log_2_ *gck* RQs; C) log_2_ *sgk2b* RQs; D) log_2_ *mxa* RQs; E) log_2_ *ifit5* RQs; F) log_2_ *fabp3a* RQs; G) log_2_ *igd* RQs. The analyses were performed using the tank mean values of each variable as AFI could only be calculated by tank. Regression lines and 95% confidence intervals are represented by solid and dashed lines, respectively. (TIF 95 kb)

## Abbreviations

*6pgd*: *6-phosphogluconate dehydrogenase*
*acac*: Acetyl-CoA carboxylase
*acly*: *ATP-citrate synthase*
*acox1*: *Acyl-coenzyme A oxidase 1*
*adssl1a*: *Adenylosuccinate synthetase isozyme 1 C-A*
AFI: Apparent feed intake
ARA: Arachidonic acid
*ccl13*: *C-C motif chemokine 13-like*
*cpt1*: *Carnitine palmitoyltransferase 1*
*dgat2*: *Diacylglycerol O-acyltransferase 2*
DHA: Docosahexaenoic acid
EPA: Eicosapentaenoic acid
FA: Fatty acid
*fabp3a*: *Fatty acid-binding protein 3*
*gadd45b*: *Growth arrest and DNA-damage-inducible protein GADD45 beta*
*gck*: *Glucokinase*
GOI: Gene of interest
*htra1*: *Serine protease HTRA1*
*idi1*: *Isopentenyl-diphosphate delta-isomerase 1*
*ifit5*: *Interferon-induced protein with tetratricopeptide repeats 5*
*igd*: *Immunoglobulin delta heavy chain*
*igm*: *Immunoglobulin mu heavy chain*
LC-PUFA: Long-chain polyunsaturated fatty acids
*lect2*: *Leukocyte cell-derived chemotaxin 2*
*mhcI*: *MHC class I antigen*
*mtco1*: *Cytochrome c oxidase subunit 1*
*mtco2*: *Cytochrome c oxidase subunit 2*
*mx*: *Interferon-induced GTP-binding protein Mx*
*pfkfb4*: *6-phosphofructo-2-kinase/fructose-2,6-biphosphatase 4-like*
PFP: Percentage of false-positives
SFA: Saturated fatty acids
*sgk2*: *Serine/threonine-protein kinase Sgk2*
*sqs*: *Squalene synthase*
WG: Weight gain
ω3: Omega-3
ω6: Omega-6
