# Supplementary material for: Changes in the liver transcriptome of farmed Atlantic salmon (Salmo salar) fed experimental diets based on terrestrial alternatives to fish meal and fish oil
Source: BMC Genomics. 2018 Nov 3;19:796. doi: 10.1186/s12864-018-5188-6 (PMC6215684; doi:10.1186/s12864-018-5188-6)
Supplement: Supplementary file 1 — Table S1. Primers used for qPCR analyses. (DOCX 21 kb) [file 12864_2018_5188_MOESM1_ESM.docx]

Table S1. Primers used for qPCR analyses.

| Gene name (symbol) | GenBank accession number | Nucleotide sequence (5'-3') | Size (bp) | Efficiency (%) |
| --- | --- | --- | --- | --- |
| *Glucokinase* (*gck*) | XM_014171080^1^ | F: CTTTGGAGCCAACGGAGA | 131 | 99.8 |
|  |  | R: GCACCAGCTCTCCCATGTA |  |  |
| *6-phosphofructo-2-kinase/fructose-2,6-biphosphatase 4-like* (*pfkfb4*) | BT044025 | F: CACGGAAGTGAAAGTGAGCA | 113 | 90.0 |
|  |  | R: CGTCCAAAGGCACATAGGTT |  |  |
| *6-phosphogluconate dehydrogenase* (*6pgd*) | BT059099 | F: GAAGAGATGAGGGGCATTTTC | 100 | 100.0 |
|  |  | R: TCTGATTGAAGGAAGGGGTCT |  |  |
| *Adenylosuccinate synthetase isozyme 1 C-A* (*adssl1a*) | NM_001139706^2^ | F: TGGAATTCAGGAGACCCAGA | 104 | 91.3 |
|  |  | R: GTCCTATGCGAGATGCCTTG |  |  |
| *Acetyl-CoA carboxylase* (*acac*) | DY706703 | F: TTTTGATGGCGATCTTGACA | 139 | 104.5 |
|  |  | R: CATCACAATGCCTCGCTCTA |  |  |
| *Diacylglycerol O-acyltransferase 2 A* (*dgat2a*) | BQ036283 | F: AAACCTCTTAGGGGCTTCCA | 176 | 106.8 |
|  |  | R: ACCTAGGATCATTGGCTGGAT |  |  |
| *Diacylglycerol O-acyltransferase 2 B* (*dgat2b*) | EG878494 | F: CGCACTTATTGTGGGGTTCT | 185 | 93.1 |
|  |  | R: TCAACATTTCACCACATGGAA |  |  |
| *Fatty acid-binding protein 3 A* (*fabp3a*) | NM_001123578 | F: CGATAGACGGTGGTAAGATG | 120 | 98.8 |
|  |  | R: GAGACGACGTCACCCAGAGT |  |  |
| *Fatty acid-binding protein 3 B* (*fabp3b*) | BT050105 | F: AACCCACCACCATCATTGAG | 102 | 90.9 |
|  |  | R: CGAACTCCTCTCCCAGTTTG |  |  |
| *Acyl-coenzyme A oxidase 1* (*acox1*) | DW555884 | F: GTGCACCTACGAGGGAGAGA | 111 | 103.0 |
|  |  | R: TAGGACACGATGCCACTCAG |  |  |
| *Carnitine palmitoyltransferase 1* (*cpt1*)^4^ | EG857609 | F: TAGCCACCATGAGCACCATA | 136 | 96.5 |
|  |  | R: GTGGAAAACAGGATGGCACT |  |  |
| *ATP-citrate synthase* (*acly*) | DY705688 | F: GGATACAGATGAGTGTCTCCCTATTT | 111 | 107.3 |
|  |  | R: GTGGGAGATTCAGTCTCATCAAC |  |  |
| *Isopentenyl-diphosphate delta-isomerase 1* (*idi1*) | DY710563 | F: GTCGGGATGCCTGAAATAAA | 129 | 97.8 |
|  |  | R: GAGGTGGCAGTTCTTTTTGC |  |  |
| *Squalene synthase* (*sqs*) | BT072164 | F: GACAAGGAGAGGAAGCCACA | 102 | 96.0 |
|  |  | R: GCCTGCTACTATGTGCTCTGC |  |  |
| *Serine/threonine-protein kinase Sgk2 A* (*sgk2a*) | NM_001139943 | F: AGCCATCGCTGACTTTCTACA | 174 | 92.3 |
|  |  | R: CCCACTGAGTTAGGCACCAT |  |  |
| *Serine/threonine-protein kinase Sgk2 B* (*sgk2b*) | BT059352 | F: AATTCCTCAACCCCAAATCC | 161 | 99.9 |
|  |  | R: CTGCGCATAGCCTACACAAA |  |  |
| *Serine protease HTRA1 A* (*htra1a*) | NM_001141717 | F: GCTGATGTGGTGGAGGAGAT | 127 | 102.2 |
|  |  | R: TCAAGCCGTCCTCTGACAC |  |  |
| *Serine protease HTRA1 B* (*htra1b*) | EG831192 | F: ATGATGACTCTCACACCAATGC | 104 | 100.9 |
|  |  | R: GTTTTTGGGATGACCTCGATT |  |  |
| *Growth arrest and DNA-damage-inducible protein GADD45 beta A* (*gadd45ba*) | BT047350 | F: CGATGGCTGTCAGTTAAGAGG | 140 | 106.1 |
|  |  | R: AATTCAGCAATGCAGTTAGTTCAC |  |  |
| *Growth arrest and DNA-damage-inducible protein GADD45 beta B* (*gadd45bb*) | EG900267 | F: GAGGAGGGACAAAGCAACTG | 152 | 105.2 |
|  |  | R: TGCACTGTTACTCATAACATCCAAC |  |  |
| *Leukocyte cell-derived chemotaxin* *2 A* (*lect2a*) | BT059281 | F: CAGATGGGGACAAGGACACT | 150 | 91.0 |
|  |  | R: GCCTTCTTCGGGTCTGTGTA |  |  |
| *Leukocyte cell-derived chemotaxin 2 B* (*lect2b*) | DV106130 | F: ACAACTGGGGACAAGGACAG | 125 | 90.0 |
|  |  | R: CACTTTGCCGTTGAGTTTCA |  |  |
| *Immunoglobulin mu heavy chain A* (*igma*) | BT058702 | F: AGCATTCACTTGCGTGTTTG | 115 | 90.4 |
|  |  | R: CGGGGTGATCTTAATGACTACTG |  |  |
| *Immunoglobulin mu heavy chain B* (*igmb*) | BT059185 | F: GAAGTTTCATTCACTTGCGTGT | 121 | 92.0 |
|  |  | R: GCGGGATGATGTTAATGACC |  |  |
| *Immunoglobulin delta heavy chain* (*igd*) | AF141606 | F: ACCCTAGGAGTTCAACTGGAAAG | 138 | 106.3 |
|  |  | R: AAACCTGCAACAGGAAAATGTAA |  |  |
| *Interferon-induced GTP-binding protein Mx A* (*mxa*)^3^ | U66475 | F: CTGAAAAGCGGAGTTCGTCT | 112 | 91.0 |
|  |  | R: CTCCCTCGATCCTCTGGTTA |  |  |
| *Interferon-induced GTP-binding protein Mx B* (*mxb*)^3^ | BT044881 | F: ACGCACCACTCTGGAGAAAT | 184 | 91.8 |
|  |  | R: CTTCCATTTCCCGAACTCTG |  |  |
| *Interferon-induced protein with tetratricopeptide repeats 5* (*ifit5*)^3^ | BT046021 | F: AGAGAGGTGCCAGGCTAACA | 149 | 97.5 |
|  |  | R: GGGCATCTGTGAGGTCATCT |  |  |
| *MHC class I antigen* (*mhcI*)^3^ | AF504022 | F: CATGAAGATGTGGAGCATGG | 131 | 94.7 |
|  |  | R: AGACCCGTGACTTGAACCAC |  |  |
| *Cytochrome c oxidase subunit 1* (*mtco1*) | BT044032 | F: CCTTCTGGGAGATGACCAAA | 188 | 100.1 |
|  |  | R: AGGAGGGAGGGAGAAGTCAA |  |  |
| *Cytochrome c oxidase subunit 2 A* (*mtco2a*) | BT044012 | F: CGAAATTAATGACCCACACCTTA | 141 | 90.0 |
|  |  | R: GACGCGGATTGGAGATTCTA |  |  |
| *Cytochrome c oxidase subunit 2 B* (*mtco2*b) | DW556807 | F: ACGAAGACTTGGGCTTTGATT | 119 | 96.6 |
|  |  | R: GACGCGGATTGGAAATTCT |  |  |
| *60S ribosomal protein L32* (*rpl32*)^4^ | BT043656 | F: AGGCGGTTTAAGGGTCAGAT | 119 | 94.3 |
|  |  | R: TCGAGCTCCTTGATGTTGTG |  |  |
| *Elongation factor 1 alpha 1* (*eef1a1*)^4^ | AF321836 | F: TGGCACTTTCACTGCTCAAG | 197 | 93.6 |
|  |  | R: CAACAATAGCAGCGTCTCCA |  |  |

^1^ No *Salmo salar* ESTs were found for *gck*. However, the 60mer microarray probe (C035R008) shared 97% similarity with a predicted cDNA sequence for *Salmo salar* *gck*, which was used for qPCR primers design.

^2^ The cDNA sequence is annotated as ‘*Salmo salar* adenylosuccinate synthase like 2’ in NCBI nr/nt database. However, best BLASTx-hits correspond to *Salmo salar* and other species’ ‘adenylosuccinate synthetase isozyme 1 C’. Therefore, we named the qPCR-analyzed paralogue as ‘adenylosuccinate synthetase isozyme 1 C-A (*adssl1a*)’.

^3^ Primers previously designed in Caballero-Solares et al. [28], and quality-tested again using the reference cDNA template of the present study.

^4^ Primers previously designed in Xue et al. [35], and quality-tested again using the reference cDNA template of the present study.
