# Supplementary material for: Changes in the liver transcriptome of farmed Atlantic salmon (Salmo salar) fed experimental diets based on terrestrial alternatives to fish meal and fish oil
Source: BMC Genomics. 2018 Nov 3;19:796. doi: 10.1186/s12864-018-5188-6 (PMC6215684; doi:10.1186/s12864-018-5188-6)
Supplement: Supplementary file 3 — Figure S1. Alignment of nucleotide sequences corresponding to adssl1a and adssl1b. Conserved nucleotides in the aligned sequences are highlighted in blue. Adssl1a and adssl1b sequences share 93% identity over 597 aligned nucleotides. The alignment and percentage identity calculation were performed using AlignX (Vector NTI Advance 11). The nucleotide regions covered by probes C107R157 and C098R022 from the Agilent 44 K salmonid microarray (GEO accession number: GPL11299) are indicated within boxes. The forward qPCR primer for adssl1a is in bold and single underlined, whereas the reverse qPCR primer is in bold and double underlined. The qPCR primers designed for adssl1b are not included in the figure as none of them passed our quality tests. (DOCX 28 kb) [file 12864_2018_5188_MOESM3_ESM.docx]

**Figure S1. Alignment of nucleotide sequences corresponding to *adssl1a* and *adssl1b*.**

1 50

adssl1a_NM_001139706 (1) -----------------------------------------------GGA

adssl1b_XM_014210487 (1) CTGATATGGCAATGCTACTGTTAGAAACATTCTAGTCTGTTTCAGACAGA

adssl1b_DN163954 (1) --------------------------------------------------

adssl1b_GE623728 (1) --------------------------------------------------

51 100

adssl1a_NM_001139706 (4) CAGCATAAGCAACATCATGTCGTTTAGCTGGTCAGCAAAAGACCACAAGA

adssl1b_XM_014210487 (51) CAGCCTAAGCAACATCATGTCGTTGAGCTGGTCAGCAAAAGACCACAAGA

adssl1b_DN163954 (1) ---------CAACATCATGTCGTTGAGCTGGTCAGCAAAAGACCACAAGA

adssl1b_GE623728 (1) --------------------------------------------------

101 150

adssl1a_NM_001139706 (54) GTTATACAAATCCACCCTCCAACCCAACCCAAGGGCTGAAGCGGCCACGG

adssl1b_XM_014210487 (101) GTTATACAAATTCACCCTCCAACCCTACCCAAGGGCTGAAGCGGCCACGG

adssl1b_DN163954 (42) GTTATACAAATTCACCCTCCAACCCTACCCAAGGGCTGAAGCGGCCACGG

adssl1b_GE623728 (1) --------------------------------------------------

151 200

adssl1a_NM_001139706 (104) AACGACACAGGGAACAAAGTGACAGTAGTGCTCGGTGCGCAATGGGGAGA

adssl1b_XM_014210487 (151) AATGACGCAGGGAACAAAGTGACAGTGGTGCTCGGTGCGCAATGGGGAGA

adssl1b_DN163954 (92) AATGACGCAGGGAACAAAGTGACAGTGGTGCTCGGTGCGCAATGGGGAGA

adssl1b_GE623728 (1) --------------------------------------------------

201 250

adssl1a_NM_001139706 (154) TGAAGGCAAAGGAAAAGTCGTCGATTTATTGGCGACTGAGTCTGACCTTG

adssl1b_XM_014210487 (201) TGAAGGCAAAGGAAAAGTCGTCGATTTACTGGCGACTGAGTCTGACCTTG

adssl1b_DN163954 (142) TGAAGGCAAAGGAAAAGTCGTCGATTTACTGGCGACTGAGTCTGACCTTG

adssl1b_GE623728 (1) --------------------------------------------------

251 300

adssl1a_NM_001139706 (204) TTTGCAGATGTCAGGGCGGTAACAATGCAGGCCACACAGTGGTTGTGGAA

adssl1b_XM_014210487 (251) TTTGCAGATGTCAGGGTGGTAACAATGCAGGCCACACAGTGGTAGTAGAA

adssl1b_DN163954 (192) TTTGCAGATGTCAGGGTGGTAACAATGCAGGCCACACAGTGGTAGTAGAA

adssl1b_GE623728 (1) --------------------------------------------------

301 350

adssl1a_NM_001139706 (254) GGCAAAGAGTATGACTTCCACCTTCTCCCCAGTGGAATTATCAACCCCAA

adssl1b_XM_014210487 (301) GGCACAGAGTATGACTTCCACCTTCTCCCCAGTGGCATTATCAACCCCAA

adssl1b_DN163954 (242) GGCACAGAGTATGACTTCCACCTTCTCCCCAGTGGCATTATCAACCCCAA

adssl1b_GE623728 (1) --------------------------------------------------

351 400

adssl1a_NM_001139706 (304) AAGTATATGTGTCATTGGTAATGGCGTAGTCATACACCTACCAGGTTTGT

adssl1b_XM_014210487 (351) AAGCATATGTGTCATTGGTAATGGCGTAGTCATACACCTACCGGGCTTGT

adssl1b_DN163954 (292) AAGCATATGTGTCATTGGTAATGGCGTAGTCATACACCTACCGGGCTTGT

adssl1b_GE623728 (1) --------------------------------------------------

401 450

adssl1a_NM_001139706 (354) TTGAGGAGGCAGAGAACAATGAGAAGAAAGGTCTCAAAGGCTGGGAGAAG

adssl1b_XM_014210487 (401) TTGAGGAGGCGGAGAAGAATGAGAAGAAAGGTCTCAAAGGCTGGGAGAAG

adssl1b_DN163954 (342) TTGAGGAGGCGGAGAAGAATGAGAAGAAAGGTCTCAAAGGCTGGGAGAAG

adssl1b_GE623728 (1) --------------------------------------------------

451 500

adssl1a_NM_001139706 (404) AGACTAATAGTCTCTGACAGAGCTCACCTTGTGTTTGATTTCCATCAGGT

adssl1b_XM_014210487 (451) AGACTAATAGTCTCTGACAGAGCTCACCTCGTGTTTGATTTCCACCAGGT

adssl1b_DN163954 (392) AGACTAATAGTCTCTGACAGAGCTCACCTCGTGTTTGATTTCCACCAGGT

adssl1b_GE623728 (1) --------------------------------------------------

501 550

adssl1a_NM_001139706 (454) TGTGGA**TGGAATTCAGGAGACCCAGA**GACAAGCAACAGAGGGAAAGATAA

adssl1b_XM_014210487 (501) TGTGGATGGAATTCAGGAGAGCCAGCGACAAGCAACAGAGGGAAAGATTA

adssl1b_DN163954 (442) TGTGGATGGAATTCAGGAGAGCCAGCGACAAGCAACAGAGGGAAAGATTA

adssl1b_GE623728 (1) --------------------------------------------------

551 600

adssl1a_NM_001139706 (504) TTGGAACAACCAAGAAAGGCATTGGACCCACCTATGCCAG**CAAGGCATCT**

adssl1b_XM_014210487 (551) TTGGAACGACCAAGAAAGGCATTGGACCCACCTATACCAGCAAAGCATCT

adssl1b_DN163954 (492) TTGGAACGACCAAGAAAGGCATTGGACCCACCTATACCAGCAAAGCATCT

adssl1b_GE623728 (1) --------------------------------------------------

601 650

adssl1a_NM_001139706 (554) **CGCATAGGAC**TGCGTGTCTGTGACCTGCTGGGAGACTTCAAGGAGTTCTC

adssl1b_XM_014210487 (601) CGCATTGGACTGCGTGTCTGTGACCTGCTGGGAGACTTTAAAGAGTTCTC

adssl1b_DN163954 (542) CGCATTGGACTGCGTGTCTGTGACCTGCTGGGAGACTTTAAAGAGTTCTC

adssl1b_GE623728 (1) --------------------------------------------------

651 700

adssl1a_NM_001139706 (604) TACCAAATTCAAGAACCTTGTCGAACAGTACCAGTCCATGTACTCATCCC

adssl1b_XM_014210487 (651) TACCAAATTCAAGAACCTTGTCGCGCAGTACCAGTCCATGTACTCATCCC

adssl1b_DN163954 (592) TACCAA--------------------------------------------

adssl1b_GE623728 (1) -----------------------------------------ACTCATCCC

701 750

adssl1a_NM_001139706 (654) TGACAGTTGATACTGAAAGTCAGCTGAAAAAACTGAAGGAGTATGGAGAG

adssl1b_XM_014210487 (701) TGACAGTTGATACTGATACTCAGCTGAAAAAACTGAAGGAGTATGGAGAG

adssl1b_DN163954 (598) --------------------------------------------------

adssl1b_GE623728 (10) TGACAGTTGATACTGATACTCAGCTGAAAAAACTGAAGGAGTATGGAGAG

751 800

adssl1a_NM_001139706 (704) AGGTTGCGGCCGATGGTTCGGGATGGAGTCTACTACATGTACGAGGCTCT

adssl1b_XM_014210487 (751) AGGTTGCGGCCGATGGTGCGGGATGGAGTCTACTACATGTATGAGGCTCT

adssl1b_DN163954 (598) --------------------------------------------------

adssl1b_GE623728 (60) AGGTTGCGGCCGATGGTGCGGGATGGAGTCTACTACATGTATGAGGCTCT

801 850

adssl1a_NM_001139706 (754) TCATGGACCCCCAAAGAAAATTCTGGTGGAAGGGGCCAACGCTGCCCTCC

adssl1b_XM_014210487 (801) TCATGGACCCCCAAAGAAAATTCTGGTGGAAGGGGCCAATGCTGCCCTCC

adssl1b_DN163954 (598) --------------------------------------------------

adssl1b_GE623728 (110) TCATGGACCCCCAAAGAAAATTCTGGTGGAAGGGGCCAATGCTGCCCTCC

851 900

adssl1a_NM_001139706 (804) TCGACATTGACTTTGGCACATATCCTTTTGTGACCTCATCAAACTGCACC

adssl1b_XM_014210487 (851) TCGACATTGACTTTGGCACATATCCTTTTGTGACCTCATCAAACTGCACT

adssl1b_DN163954 (598) --------------------------------------------------

adssl1b_GE623728 (160) TCGGCATTGACTTTGGCACATATCCTTTTGTGACCTCATCAAACTGCACT

901 950

adssl1a_NM_001139706 (854) GTTGGTGGGGCATGCACTGGTCTTGGCATCCCTCCTCTGAATATTGGTGA

adssl1b_XM_014210487 (901) GTTGGTGGGGCATGCACTGGTCTTGGCATCCCTCCCCTGAATATTGGTGA

adssl1b_DN163954 (598) --------------------------------------------------

adssl1b_GE623728 (210) GTTGGTGGGGCATGCACTGGTCTTGGCATCCCTCCCCTGAATATTGGTGA

951 1000

adssl1a_NM_001139706 (904) AGTGTATGGTGTATCAAAGGCCTACACCACCAGGGTGGGAATTGGTGCCT

adssl1b_XM_014210487 (951) AGTGTATGGTGTATCAAAGGCCTACACCACCAGGGTAGGAATTGGTGCCT

adssl1b_DN163954 (598) --------------------------------------------------

adssl1b_GE623728 (260) AGTGTATGGTGTATCAAAGGCCTACACCACCAGGGTAGGAATTGGTGCCT

1001 1050

adssl1a_NM_001139706 (954) TCCCAACAGAACAACTCAATGCAACAGGTGAGCTGCTGCAGACAAGGGGT

adssl1b_XM_014210487 (1001) TCCCCACAGAACAGCTCAATGCGACAGGTGAGCTGCTGCAGACGAGAGGT

adssl1b_DN163954 (598) --------------------------------------------------

adssl1b_GE623728 (310) TCCCCACAGAACAGCTCAATGCGACAGGTGAGCTGCTGCAGACGAGAGGT

1051 1100

adssl1a_NM_001139706 (1004) CATGAGGTGGGCGTGACCACAGGCAGGAAACGTCGCTGTGGCTGGCTGGA

adssl1b_XM_014210487 (1051) CATGAGGTGGGCGTGACAACGGGCAGGAAACGTCGCTGTGGCTGGCTGGA

adssl1b_DN163954 (598) --------------------------------------------------

adssl1b_GE623728 (360) CATGAGGTGGGCGTGACAACGGGCAGGAAACGTCGCTGTGGCTGGCTGGA

1101 1150

adssl1a_NM_001139706 (1054) CCTGGTCATCCTGAGATACGCTCACATGATCAATGGCTTCACTGCAATTG

adssl1b_XM_014210487 (1101) CCTGGTCATCCTGAGATACGCCCACATGATCAATGGCTTCACTGCCATTG

adssl1b_DN163954 (598) --------------------------------------------------

adssl1b_GE623728 (410) CCTGGTCATCCTGAGATACGCCCACATGATCAATGGCTTCACTGCCATTG

1151 1200

adssl1a_NM_001139706 (1104) CTTTGACAAAACTTGACATCCTTGATGTGCTGGATGAGATCAAAGTAGGA

adssl1b_XM_014210487 (1151) CTTTGACAAAACTTGACATCCTTGATGTGCTGGATGAGATCAAAGTAGGA

adssl1b_DN163954 (598) --------------------------------------------------

adssl1b_GE623728 (460) CTTTGACAAAACTTGACATCCTTGATGTGCTGGATGAGATCAAAGTAGGA

1201 1250

**C107R157**

adssl1a_NM_001139706 (1154) ATGGCCTACAAAATCAATGGCAAAAGAATTCCCCATTTCCCAGCTGACAT

adssl1b_XM_014210487 (1201) GTGGCCTACAAAATCAGTGGCAAAAGAATTCCCCATTTCCCAGCTAACAT

adssl1b_DN163954 (598) --------------------------------------------------

adssl1b_GE623728 (510) GTGGCCTACAAAATCAGTGGCAAAAGAATTCCCCATTTCCCAGCTAACAT

1251 1300

adssl1a_NM_001139706 (1204) GGAGCTGTTGCACAAAGTGGAGGTAGAGTATGAGACCTTCCCCGGCTGGA

adssl1b_XM_014210487 (1251) GGAGCTGTTGCACAAAGTGGAGGTTGAGTATGAGACCTTCCCAGGCTGGA

adssl1b_DN163954 (598) --------------------------------------------------

adssl1b_GE623728 (560) GGAGCTGTTGCACAAAGTGGAGGTTGAGTATGAGACCTTCCCAGGCTGGA

1301 1350

adssl1a_NM_001139706 (1254) AGAGTGACACGTCTGCAGCCAGGAAGTGGAATAATCTCCCCCAGAAGGCT

adssl1b_XM_014210487 (1301) AGAGTGATACGTCTGCAGCCAGGAAGTGGAATGATCTCCCCCAGAAGGCT

adssl1b_DN163954 (598) --------------------------------------------------

adssl1b_GE623728 (610) AGAGTGATACGTCTGCAGCCAGGAAGTGGAATGATCTCCCCCAGAAGGCT

1351 1400

adssl1a_NM_001139706 (1304) CAGAACTACATCCGCTTTGTGGAGAGCCACATTGGAGTACCCATTAAGTG

adssl1b_XM_014210487 (1351) CAGAACTACATCCGCTTTGTGGAGAACCACATTGGAGTACCCATTAAGTG

adssl1b_DN163954 (598) --------------------------------------------------

adssl1b_GE623728 (660) CAGAACTACATCCGCTTTGTGGAGAACCACATTGGAGT------------

1401 1450

adssl1a_NM_001139706 (1354) GGTCGGTGTAGGAAAGTCCAGAGAGTGCATGATCCAGATGTTCTAGAGAG

adssl1b_XM_014210487 (1401) GGTCGGCGTCGGAAAGTCCAGAGAGTGCATGATCCAGATGTTCTAGAGAC

adssl1b_DN163954 (598) --------------------------------------------------

adssl1b_GE623728 (698) --------------------------------------------------

1451 1500

adssl1a_NM_001139706 (1404) TTTACCTCCTCTCTCCCCAAGATGGATGCGCAA---------AGATGTGG

adssl1b_XM_014210487 (1451) TTTTCCTTTTCTCTCCCCAAGATGGATGCACATGGATGGACGAGATGTGG

adssl1b_DN163954 (598) --------------------------------------------------

adssl1b_GE623728 (698) --------------------------------------------------

1501 1550

adssl1a_NM_001139706 (1445) CATGATGTGACATATGTTTGCAACCCCCAAACTGTCTAATATTAGGTAAA

adssl1b_XM_014210487 (1501) CTTGATGTGACATAAGTTTACAATCCTCAAACTATCTAATATTCTGTAAA

adssl1b_DN163954 (598) --------------------------------------------------

adssl1b_GE623728 (698) --------------------------------------------------

1551 1600

adssl1a_NM_001139706 (1495) AAAAACTTATGCCAACAGATTTGTGTAGCTTTGAAACAATGTTGACTGCT

adssl1b_XM_014210487 (1551) AC---CTGTTAACAACAAATACGTGTAGCCTTGAAACAATGTTGAGTGGA

adssl1b_DN163954 (598) --------------------------------------------------

**C098R022**

adssl1b_GE623728 (698) --------------------------------------------------

1601 1650

adssl1a_NM_001139706 (1545) AATGTTGGTAACTTTACATCTATCTATGCAGTGCTTCTCATCAGTAAGTT

adssl1b_XM_014210487 (1598) AACGTTGGTAACTTTACATCTATCTGTGTTG-------CATCAGTAAATT

adssl1b_DN163954 (598) --------------------------------------------------

adssl1b_GE623728 (698) --------------------------------------------------

1651 1700

adssl1a_NM_001139706 (1595) GCCTTGTGATGATGTCGGCCAGAAAGTCCTA-----TTACTGACCCAGGA

adssl1b_XM_014210487 (1641) GCCTTGTAGTGATGTCTGCCAGAAAGTCCTACAGTATGACTGAGCCATGT

adssl1b_DN163954 (598) --------------------------------------------------

adssl1b_GE623728 (698) --------------------------------------------------

1701 1750

adssl1a_NM_001139706 (1640) GTAAATCTTACCTTGTGCCACACAACAAACATGAAAGATAAAAACACATC

adssl1b_XM_014210487 (1691) GTAAATCTTACCTTGTGCCACACAACAACCCAGAAAGAAAAAAACACATC

adssl1b_DN163954 (598) --------------------------------------------------

adssl1b_GE623728 (698) --------------------------------------------------
