# Supplementary material for: Changes in the liver transcriptome of farmed Atlantic salmon (Salmo salar) fed experimental diets based on terrestrial alternatives to fish meal and fish oil
Source: BMC Genomics. 2018 Nov 3;19:796. doi: 10.1186/s12864-018-5188-6 (PMC6215684; doi:10.1186/s12864-018-5188-6)
Supplement: Supplementary file 4 — Figure S2. Alignment of nucleotide sequences corresponding to dgat2a and dgat2b. Nucleotides conserved in all sequences are highlighted in yellow, and in blue for those nucleotides shared by three sequences from different paralogues, or by two but the nucleotide is deleted in the third (or the sequence is not long enough); finally, non-conserved nucleotides between paralogues are indicated by highlighting the nucleotides of one of them in green. “Rc” after the GenBank accession numbers stands for reverse complement. Dgat2a and dgat2b sequences share 83% identity over 2170 aligned nucleotides. The alignment and percentage identity calculation were performed using AlignX (Vector NTI Advance 11). The nucleotide regions covered by probes C103R066 and C134R089 from the Agilent 44 K salmonid microarray (GEO accession number: GPL11299) are indicated within boxes. Forward qPCR primers are in bold and single underlined, whereas reverse qPCR primers are in bold and double underlined. (DOCX 38 kb) [file 12864_2018_5188_MOESM4_ESM.docx]

**Figure S2. Alignment of nucleotide sequences corresponding to *dgat2a* and *dgat2b*.**

1 50

dgat2a_ XM_014197366 (1) TCAAAACACGATCGACGCCGACATCACGTCACTGCAGTACCTGATAAATA

dgat2a_BQ036283rc (1) --------------------------------------------------

dgat2b_XM_014137863 (1) --------------------------------------------------

dgat2b_EG878494rc (1) --------------------------------------------------

51 100

dgat2a_ XM_014197366 (51) CGTGGTTGAGAGTACTTTCAGACCACCGCAGGCTGACTTGAGAAAAATTA

dgat2a_BQ036283rc (1) --------------------------------------------------

dgat2b_XM_014137863 (1) --------------------------------------------------

dgat2b_EG878494rc (1) --------------------------------------------------

101 150

dgat2a_ XM_014197366 (101) TGTTTTCGAGTAGCGCAATTTGCATCCTCTTCAGTTCTTCTACAGGAGCG

dgat2a_BQ036283rc (1) --------------------------------------------------

dgat2b_XM_014137863 (1) --------------------------------------------------

dgat2b_EG878494rc (1) --------------------------------------------------

151 200

dgat2a_ XM_014197366 (151) CATCGGTTTGGACCCAGTACACCTCCCACCCTCCCTCACGTACTGACTTT

dgat2a_BQ036283rc (1) --------------------------------------------------

dgat2b_XM_014137863 (1) ------------------------------CCCCCTCCCATACTGACTGT

dgat2b_EG878494rc (1) --------------------------------------------------

201 250

dgat2a_ XM_014197366 (201) GAACTGGCTCCTCCTATACGGGTTAAATGC-CGTTCAGTTATTCAATTTA

dgat2a_BQ036283rc (1) --------------------------------------------------

dgat2b_XM_014137863 (21) GA-CTGGCTCCTCCCATACCGGTTAAATGCGCGCTCGGTGCTTCAATTCA

dgat2b_EG878494rc (1) --------------------------------------------------

251 300

dgat2a_ XM_014197366 (250) AT----------GCAGTCATGATAGAGGAACTCTAGAGTTTATTAACTTT

dgat2a_BQ036283rc (1) --------------------------------------------------

dgat2b_XM_014137863 (70) GTACAGTGGAAGGCAGTCATGATAGAGGAGCTGTAGAGTTTACAAACTTT

dgat2b_EG878494rc (1) --------------------------------------------------

301 350

dgat2a_ XM_014197366 (290) ATTGTAGTAAATATTTCCAAGTATTGTAACCTGTTAATTCGCGGAGCCAC

dgat2a_BQ036283rc (1) --------------------------------------------------

dgat2b_XM_014137863 (120) AAAGCAGCAAGTATTTCCAAGTCTTGTAATCTGTTTATTCCCGGGGCCAC

dgat2b_EG878494rc (1) --------------------------------------------------

351 400

dgat2a_ XM_014197366 (340) AGTTATGAAGACAATACTTGCTGCCTACCCCGGTGTCCTAAAAGGCACCG

dgat2a_BQ036283rc (1) --------------------------------------------------

dgat2b_XM_014137863 (170) AGTTATGAAGACCATACTTGCTGCCTACTCCGGTGCCCTAAAAGGCACAG

dgat2b_EG878494rc (1) --------------------------------------------------

401 450

dgat2a_ XM_014197366 (390) GCTGCAGCATCCTCTCCGCCCTGCAGGACCTACCCTCTGCTCCTTGGCCT

dgat2a_BQ036283rc (1) --------------------------------------------------

dgat2b_XM_014137863 (220) GCTATAGCATCCTCTCGTCCCTGCAGGACCTGCCCTCCCCTCCATGGCCT

dgat2b_EG878494rc (1) --------------------------------------------------

451 500

dgat2a_ XM_014197366 (440) GCACTCCGATCCAAGATGGAGAAACACCTTCAGGTCATCTCAGTTCTGCA

dgat2a_BQ036283rc (1) --------------------------------------------------

dgat2b_XM_014137863 (270) GCCCTTCGATCCAAGATGGAGAAACACCTTCAGGTCATCGCAGTTCTTCA

dgat2b_EG878494rc (1) --------------------------------------------------

501 550

dgat2a_ XM_014197366 (490) GTGGGTCATCAGCTTCCTCGCCATGGGTGCCGCTGGCACTGTGCTCTTAA

dgat2a_BQ036283rc (1) --------------------------------------------------

dgat2b_XM_014137863 (320) GTGGGTCATCTCCTTCCTTGCCATGGGCATCAGCTGCACTGTGCTGTTAA

dgat2b_EG878494rc (1) --------------------------------------------------

551 600

dgat2a_ XM_014197366 (540) TCTACATGTTCTGCACAGACCTCTGGGTGATCGCTGCCATGTACACTGCC

dgat2a_BQ036283rc (1) --------------------------------------------------

dgat2b_XM_014137863 (370) TCTACATGTTCTGCACAGACCTCTGGGTGATCGCTGCCATGTACACTACC

dgat2b_EG878494rc (1) --------------------------------------------------

601 650

dgat2a_ XM_014197366 (590) TGGCTCATCTTCGACTGGAACACCCCCAAACAAGGTGGCAGGAGGTCCTC

dgat2a_BQ036283rc (1) --------------------------------------------------

dgat2b_XM_014137863 (420) TGGCTAATCTTCGACTGGAACACCCCCAAACAAGGTGGCAGGAGGTCCTC

dgat2b_EG878494rc (1) --------------------------------------------------

651 700

**C103R066**

dgat2a_ XM_014197366 (640) TTGGGTGAGGAACTGGACCATGTGGACTTACTTCAGAGACTACTTCCCCA

dgat2a_BQ036283rc (1) --------------------------------------------------

dgat2b_XM_014137863 (470) TTGGGTGAGGAACTGGACTGTGTGGACTTACTTCAGAGATTACTTTCCCA

dgat2b_EG878494rc (1) --------------------------------------------------

701 750

dgat2a_ XM_014197366 (690) TCAGGCTCATCAAGACACACAACCTGCTGCCCAGCCGAAACTACATCTTT

dgat2a_BQ036283rc (1) --------------------------------------------------

dgat2b_XM_014137863 (520) TCAGGCTCATCAAGACACACAACCTGCTGCCCAGCCGAAACTACATCTTT

dgat2b_EG878494rc (1) --------------------------------------------------

751 800

dgat2a_ XM_014197366 (740) GGCTACCACCCCCATGGGATCTTCTCTTTTGGAGCCTTCTGTAACTTCGG

dgat2a_BQ036283rc (1) --------------------------------------------------

dgat2b_XM_014137863 (570) GGGTACCACCCCCATGGGATCTTCTCTTTCGGAGCCTTCTGTAACTTCGG

dgat2b_EG878494rc (1) --------------------------------------------------

801 850

dgat2a_ XM_014197366 (790) AACAGAGGCCACCGGCTTCTCTAAGAAGTTCCCGGGCATCAAGCCTTCCC

dgat2a_BQ036283rc (1) --------------------------------------------------

dgat2b_XM_014137863 (620) TACTGAGGCCACTGGATTCTCCAAGAAGTTCCCGGGCATCAAGCCTTCTC

dgat2b_EG878494rc (1) --------------------------------------------------

851 900

dgat2a_ XM_014197366 (840) TGGCCACCCTGGCTGGAAACTTCCGGATGCCAGTCTTTAGAGACTATCTC

dgat2a_BQ036283rc (1) --------------------------------------------------

dgat2b_XM_014137863 (670) TTGCCACCCTGGCTGGAAACTTCCGGATGCCAGTCCTTAGAGACTATCTC

dgat2b_EG878494rc (1) --------------------------------------------------

901 950

dgat2a_ XM_014197366 (890) ATGTCTGGAGGTATCTGCCCAGTGAACCGTAACTCCATTGACTACCTCCT

dgat2a_BQ036283rc (1) --------------------------------------------------

dgat2b_XM_014137863 (720) ATGTCTGGGGGTATCTGCCCAGTGAACCGTAACTCCATCGACTACCTCCT

dgat2b_EG878494rc (1) --------------------------------------------------

951 1000

dgat2a_ XM_014197366 (940) CTCTCAGAATGGAACTGGCAATGCAGTGGTCATTGTTGTCGGGGGAGCAG

dgat2a_BQ036283rc (1) --------------------------------------------------

dgat2b_XM_014137863 (770) CTCTCAGAATGGAACTGGCAACGCAGTGGTCATCGTTGTCGGGGGAGCAG

dgat2b_EG878494rc (1) --------------------------------------------------

1001 1050

dgat2a_ XM_014197366 (990) CTGAATCTTTGAACTGTGCGCCAGGAAAGAATTCTGTCACCCTGAATAAC

dgat2a_BQ036283rc (1) --------------------------------------------------

dgat2b_XM_014137863 (820) CAGAATCTCTGGACTGTGCTCCAGGCATGAATTCTGTCACCCTGAATAAC

dgat2b_EG878494rc (1) --------------------------------------------------

1051 1100

dgat2a_ XM_014197366 (1040) CGCAAGGGCTTTGTGAGGTTGGCCCTCCAGCAAGGGTCTGACCTGGTACC

dgat2a_BQ036283rc (1) --------------------------------------------------

dgat2b_XM_014137863 (870) CGCAAGGGCTTTGTGAGGCTGGCCCTCCAGCAAGGATCTGACCTGGTGCC

dgat2b_EG878494rc (1) --------------------------------------------------

1101 1150

dgat2a_ XM_014197366 (1090) AGTCTACTCCTTTGGGGAGAACGATGTGTACAAACAGGTGATCTTCGAGG

dgat2a_BQ036283rc (1) --------------------------------------------------

dgat2b_XM_014137863 (920) GGTCTACTCCTTTGGGGAGAACAATGTGTACAAGCAGGTGATCTTTGAGG

dgat2b_EG878494rc (1) --------------------------------------------------

1151 1200

dgat2a_ XM_014197366 (1140) AGAGAACCTGGTGGCGGCTGGCTCAAAAGCGATTGCAGAAGATTATTGGT

dgat2a_BQ036283rc (1) --------------------------------------------------

dgat2b_XM_014137863 (970) AGGGAACCTGGTGTCGGCTAGCTCAGAAGCGGCTGCAGAAGATTCTGGGC

dgat2b_EG878494rc (1) --------------------------------------------------

1201 1250

dgat2a_ XM_014197366 (1190) TTTGCTCCCTGTCTGTTCCATGGCTGTGGCTTCTTCTCCTCCGACTCCTG

dgat2a_BQ036283rc (1) --------------------------------------------------

dgat2b_XM_014137863 (1020) TTTGCTCCCTGTCTGTTCCATGGCTGTGGCCTCTTCTC---CGACTCCTG

dgat2b_EG878494rc (1) --------------------------------------------------

1251 1300

dgat2a_ XM_014197366 (1240) GGGAATGGTGCCTTACAACAAACCCATCACCACCATCGTGGGTGAACCGA

dgat2a_BQ036283rc (1) --------------------------------------------------

dgat2b_XM_014137863 (1067) GGGAATGGTGCCTTACAATAAACCCATCACCACCATCGTGGGTGAACCGA

dgat2b_EG878494rc (1) --------------------------------------------------

1301 1350

dgat2a_ XM_014197366 (1290) TCACAGTGCCAAAGATTGAGCAGCCTCCTCGGGATATGGTGGATCTGTAC

dgat2a_BQ036283rc (1) --------------------------------------------------

dgat2b_XM_014137863 (1117) TCACGGTACCAAAGGTTGAGGAGCCTACTCGGGTTATGGTGGAACTGTAC

dgat2b_EG878494rc (1) --------------------------------------------------

1351 1400

dgat2a_ XM_014197366 (1340) CATGCCATGTACATCAATTCCCTCACGAGCCTCTTTGACAAGTATAAGAC

dgat2a_BQ036283rc (1) --------------------------------------------------

dgat2b_XM_014137863 (1167) CATGCCATGTACATCAAGTCCCTCAGGAGCCTCTTTGACAAGTATAAGAC

dgat2b_EG878494rc (1) --------------------------------------------------

1401 1450

dgat2a_ XM_014197366 (1390) CTGCTTCGGCCTGAAGGAGAGTGACATCCTGCACATCCATTGAGAAAGG-

dgat2a_BQ036283rc (1) --------------------------------------------------

dgat2b_XM_014137863 (1217) CCGCTTCGGATTGAAAGAGAGTGACATCCTGCACATCCAATGAGAAAAGA

dgat2b_EG878494rc (1) --------------------------------------------------

1451 1500

dgat2a_ XM_014197366 (1439) GTCAGC------------------------TGTGGCAGCTGTCTGTTCAG

dgat2a_BQ036283rc (1) --------------------------------------------------

dgat2b_XM_014137863 (1267) GTGAGCAGGACCCCCTCGGAACCTCAGAACTGTGGCAGCTGTTTGTTCAG

dgat2b_EG878494rc (1) --------------------------------------------------

1501 1550

dgat2a_ XM_014197366 (1465) CCCTGCCCCTCCATTCACGTGCCTGGCTCTGATTGATTGTCTCTGGAATT

dgat2a_BQ036283rc (1) --------------------------------------------------

dgat2b_XM_014137863 (1317) CCTCGACACCCCATTCACGTGCATGGCTCTGATTGATTGTCTCTGGAATT

dgat2b_EG878494rc (1) --------------------------------------------------

1551 1600

dgat2a_ XM_014197366 (1515) CAATCGATATACATAAAGCTGTG----TCTATATAAACCCACCGCGCTCT

dgat2a_BQ036283rc (1) --------------------------------------------------

dgat2b_XM_014137863 (1367) CAATCGATATACATAAAGTTGTGGGTGTGTGTATAAACCCACCGCGCTCT

dgat2b_EG878494rc (1) --------------------------------------------------

1601 1650

dgat2a_ XM_014197366 (1561) CAGCATCA--------------CTCACGTCGGTCCTTCACTCAGGATGAA

dgat2a_BQ036283rc (1) --------------------------------------------------

dgat2b_XM_014137863 (1417) CATCCTCGTCCAGTATACAAGGCTCACGTCCCTACACCACTCAGGGTGAA

dgat2b_EG878494rc (1) --------------------------------------------------

1651 1700

dgat2a_ XM_014197366 (1597) AATGGCAGGTCACCTTACTATTCTCTCTGACCTAGTCACTGTCAATGGCA

dgat2a_BQ036283rc (1) --------------------------------------------------

dgat2b_XM_014137863 (1467) AATGGCAGGT----TT--TAGGCTCCCTGACCTAGTCCTTGCCAATAGCA

dgat2b_EG878494rc (1) --------------------------------------------------

1701 1750

dgat2a_ XM_014197366 (1647) AAGTTGACTAACTTCCGTTTATTGAAT---ATTTCATTGAGTGTATGACA

dgat2a_BQ036283rc (1) --------------------------------------------------

dgat2b_XM_014137863 (1511) AAGTTGATTAACTTAAATGTCTCCAAATGGAGTCTCTTCAGCGTATGCCA

dgat2b_EG878494rc (1) --------------------------------------------------

1751 1800

dgat2a_ XM_014197366 (1694) CATCTATAGATGTGTTCCATGACAGAAAAGATGAGGGAGTATAGTT----

dgat2a_BQ036283rc (1) --------------------------------------------------

dgat2b_XM_014137863 (1561) CATTTCTAGATATGTTCCATGACAGAAGAGATGAGGGAGTATAGTTGGAT

dgat2b_EG878494rc (1) --------------------------------------------------

1801 1850

dgat2a_ XM_014197366 (1740) AAGGTATGATCCCTTGC----------AGTGGAACTCCCACTAGGTGGCA

dgat2a_BQ036283rc (1) --------------------------------------------------

dgat2b_XM_014137863 (1611) AAGATACGATCACTTGCTTTCATGTGCAGTGGAACTCCCACTAGGTGGCA

dgat2b_EG878494rc (1) --------------------------------------------------

1851 1900

dgat2a_ XM_014197366 (1780) CTAGAAGGTTACACAAGACACACACAAGCACCATCTTTGTTTCCTCCATG

dgat2a_BQ036283rc (1) --------------------------------------------------

dgat2b_XM_014137863 (1661) CTAGAAGGTTATACA---CACA-ACAAGCACCATGTTTGTTTCCCTCATG

dgat2b_EG878494rc (1) --------------------------------------------------

1901 1950

dgat2a_ XM_014197366 (1830) CATCCATGTAATTTTCACTGAGAATATGCCTGTACAAAGAATGTGTTAAG

dgat2a_BQ036283rc (1) --------------------------------------------------

dgat2b_XM_014137863 (1707) CATCCATGTAG---TCCCATATATTAGGCCC-TACAAA---------A-G

dgat2b_EG878494rc (1) --------------------------------------------------

1951 2000

dgat2a_ XM_014197366 (1880) TGGTAAAAGGTAAATATAGGATGATGTTTTGAGACCTTACATTTTTTGTT

dgat2a_BQ036283rc (1) --------------------------------------------------

dgat2b_XM_014137863 (1743) T---------TCAATATCAGA---TGTTTTGAGGCCTT---TATTTATTT

dgat2b_EG878494rc (1) --------------------------------------------------

2001 2050

dgat2a_ XM_014197366 (1930) GTTGCTAAATGCACAAACAATGCAATTGTAAGGGTGTTGCCTCACTGAGA

dgat2a_BQ036283rc (1) --------------------------------------------------

dgat2b_XM_014137863 (1778) TTTGCTAACTGCACAAACAATGCAATTGTAATGGTATGGCCTCAATCAGA

dgat2b_EG878494rc (1) --------------------------------------------------

2051 2100

dgat2a_ XM_014197366 (1980) TGTTGA---TGGTGTGGTTTATTGAGCCAAAGAGCTGATTGGTGTCCCCT

dgat2a_BQ036283rc (1) --------------------------------------------------

dgat2b_XM_014137863 (1828) AGTTTAATATAGTGTGGTA-ATTGAGCCAAAGAGTTGATGGGTGTCCCCT

dgat2b_EG878494rc (1) --------------------------------------------------

2101 2150

dgat2a_ XM_014197366 (2027) CCCCACACATGAACACTTCATTGAAACTTGAAAGTGAAGGAATTCTATTG

dgat2a_BQ036283rc (1) --------------------------------------------------

dgat2b_XM_014137863 (1877) CCCAGCACACGCATACTTCATTGAAACTGGAAGGTGAAGTAATTCTA---

dgat2b_EG878494rc (1) --------------------------------------------------

2151 2200

dgat2a_ XM_014197366 (2077) AATGGATTTAGATTGTGCATATTGGTAATGCAATCTG-ATTGTGTGATAG

dgat2a_BQ036283rc (1) --------------------------------------------------

dgat2b_XM_014137863 (1924) -ATGGATTTAGATTGTGATTATATG-ATGCCACTAAGTATTGTATTATGT

dgat2b_EG878494rc (1) --------------------------------------------TTATGT

2201 2250

dgat2a_ XM_014197366 (2126) CACTAAGTATTAAT-TGTATT---TTGTAAATAAGAACA---------TT

dgat2a_BQ036283rc (1) --------------------------------------------------

dgat2b_XM_014137863 (1972) AAATAAGAAAAAACCTGTCCTCCCTTGTTGAAAGGAACAATTTTTCTTTT

dgat2b_EG878494rc (7) AAATAAGAAAAAACCTGTCCTCCCTTGTTGAAAGGAACAATTTTTCTTTT

2251 2300

dgat2a_ XM_014197366 (2163) TTCAATGAAAATTAATATATTGATGCACTTTTTGTATTTGTGACAACCCT

dgat2a_BQ036283rc (1) --------------------------------------------------

dgat2b_XM_014137863 (2022) TTAAATTAAAATTTATATATTGATGCACTTTTTGTATTTGTTACAACCCT

dgat2b_EG878494rc (57) TTAAATTAAAATTTATATATTGATGCACTTTTTGTATTTGTTACAACCCT

**C134R089**

2301 2350

dgat2a_ XM_014197366 (2213) CTGAGGTATTTCTCTAAAACATGCCTTTGAAAGAGTCCTATGTCAAATTT

dgat2a_BQ036283rc (1) --------------------------------------------------

dgat2b_XM_014137863 (2072) CTGAGGTATTTCTCTAAAATGTGCCTTAGGAATTTTTCAATGTCACATTT

dgat2b_EG878494rc (107) CTGAGGTATTTCTCTAAAATGTGCCTTAGGAATTTTTCAATGTCACATTT

2351 2400

dgat2a_ XM_014197366 (2263) GCCAATTTCTAAGAGAATCACTATGGTGCTTTTGTCATTTAACCTTGACC

dgat2a_BQ036283rc (1) --------------------------------------------------

dgat2b_XM_014137863 (2122) T-------------------------------------------------

dgat2b_EG878494rc (157) T-------------------------------------------------

2401 2450

dgat2a_ XM_014197366 (2313) TGTGTTTTAGTTTTCTTGTACATTTCTCCACATGAGTGTATTGACTGGGT

dgat2a_BQ036283rc (1) --------------------------------------------------

dgat2b_XM_014137863 (2123) --------------------------------------TATTGACTGGGC

dgat2b_EG878494rc (158) --------------------------------------TATTGACTGGGC

2451 2500

dgat2a_ XM_014197366 (2363) TGCAGAG--TGTTACCTAGTGCATTGAGGCCCCATGTACTGTAGTGATGG

dgat2a_BQ036283rc (1) -------------------------------------ACTGTAGTGATGG

dgat2b_XM_014137863 (2135) TGCAGAGAGTGTTGTTTAGTGCCTTGATGCCCCATGTACTGTAGT-----

dgat2b_EG878494rc (170) TGCAGAGAGTGTTGTTTAGTGCCTTGATGCCCCATGTACTGTAGT-----

2501 2550

dgat2a_ XM_014197366 (2411) TGATGTAAGAACAATTTCAGGGTGAAGAACATTTCTTCCCCTACACAGAG

dgat2a_BQ036283rc (14) TGATGTAAGAACAATTTCAGGGTGAAGAACATTTCTTCCCCTACACAGAG

dgat2b_XM_014137863 (2180) ---TGTT-GACTATTCTCT--------ACTAATTCTTCCCCCACACAGAG

dgat2b_EG878494rc (215) ---TGTT-GACTATTCTCT--------ACTAATTCTTCCCCCACACAGAG

2551 2600

dgat2a_ XM_014197366 (2461) GGCAGATTACTGATGAATGAAATTAAGAGATTGCAAAGCATATGCCTTAT

dgat2a_BQ036283rc (64) GGCAGATTACTGATGAATGAAATTAAGAGATTGCAAAGCATATGCCTTAT

dgat2b_XM_014137863 (2218) GGCAGATCAGTGATGAA--------AGGGATTGTAAAGCATATGCCTTGT

dgat2b_EG878494rc (253) GGCAGATCAGTGATGAA--------AGGGATTGTAAAGCATATGCCTTGT

2601 2650

dgat2a_ XM_014197366 (2511) TTTAAAAACTACAACTCAAACCTCTTAGGGGCTTCCACTCAATTGAGTAA

dgat2a_BQ036283rc (114) TTTAAAAACTACAACTC**AAACCTCTTAGGGGCTTCCA**CTCAATTGAGTAA

dgat2b_XM_014137863 (2260) ----AAAAATAAAACGCA--CTTATTGTGGGGTTCTACTCAATTGAGGAA

dgat2b_EG878494rc (295) ----AAAAATAAAA**CGCA--CTTATTGTGGGGTTCT**ACTCAATTGAGGAA

2651 2700

dgat2a_ XM_014197366 (2561) AATGGCAAA-------------GGGGTTGCTATCACAGATTTGAAATAGA

dgat2a_BQ036283rc (164) AATGGCAAA-------------GGGGTTGCTATCACAGATTTGAAATAGA

dgat2b_XM_014137863 (2304) AATGGCAAATGTACAGTATAAGGGTTTTTCTATCACACATTTGGATTAGA

dgat2b_EG878494rc (339) AATGGCAAATGTACAGTATAAGGGTTTTTCTATCACACATTTGGATTAGA

2701 2750

dgat2a_ XM_014197366 (2598) GTATAAAAGTCACAAACTCCCCTAGTAATTACCAAGAAGTGTTTGAGAGA

dgat2a_BQ036283rc (201) GTATAAAAGTCACAAACTCCCCTAGTAATTACCAAGAAGTGTTTGAGAGA

dgat2b_XM_014137863 (2354) GT-TAAAAGTC--AAACTCCCCTGGTAATTACCAAGAAGTGTTTGAGATA

dgat2b_EG878494rc (389) GT-TAAAAGTC--AAACTCCCCTGGTAATTACCAAGAAGTGTTTGAGATA

2751 2800

dgat2a_ XM_014197366 (2648) AATCATTTTTTCATATTGGGCATATCAAGTCCATTATCCAGCCAATGATC

dgat2a_BQ036283rc (251) AATCATTTTTTCATATTGGGCATATCAAGTCCATT**ATCCAGCCAATGATC**

dgat2b_XM_014137863 (2401) AAT-------T---ACTGGGCGTATCAAGTC---TATCAAT--TATTTTT

dgat2b_EG878494rc (436) AAT-------T---ACTGGGCGTATCAAGTC---TATCAAT--TATTT**TT**

2801 2850

dgat2a_ XM_014197366 (2698) CTAGGTGCAGAAATGTATGTATGTTGTATATTA-TGCAAGATAAAAG-GA

dgat2a_BQ036283rc (301) **CTAGGT**GCAGAAATGTATGTATGTTGTATATTA-TGCAAGATAAAAG-GA

dgat2b_XM_014137863 (2436) CCATGTGGTGAAATGT-TG-AGGTTGGTGATGAGTGCTAAATAGAAGTGT

dgat2b_EG878494rc (471) **CCATGTGGTGAAATGT-TG-A**GGTTGGTGATGAGTGCTAAATAGAAGTGT

2851 2900

dgat2a_ XM_014197366 (2746) C-TACGAGATTTCACCTCATG--GCAAAATAATTGAGATT--------GG

dgat2a_BQ036283rc (349) C-TACGAGATTTCACCTCATG--GCAAAATAATTGAGATT--------GG

dgat2b_XM_014137863 (2484) CATTTGAAATGTAAATTCTTCTAGCTTCAACATTGACTTCTTGTAAAGGG

dgat2b_EG878494rc (519) CATTTGAAATGTAAATTCTTCTAGCTTCAACATTGACTTCTTGTAAAGGG

2901 2950

dgat2a_ XM_014197366 (2785) TGAGAAATAGTGTGTCATTCAAAATGTAATTTCTTCTAGCTTCATCGGTG

dgat2a_BQ036283rc (388) TGAGAAATAGTGTGTCATTCAAAATGTAATTTCTTCTAGCTTCATCGGTG

dgat2b_XM_014137863 (2534) GCAGTAAACATCTCACTCTCTTAATTAATGTGCAGTTAACAGCAGCCAAG

dgat2b_EG878494rc (569) GCAGTAAACATCTCACTCTCTTAATTAATGTGCAGTTAACAGCAGCCAAG

2951 3000

dgat2a_ XM_014197366 (2835) ACTTTT--TGCAAA-GAGCAG--TGAACATCCCGCTCTCCTAATTCATGT

dgat2a_BQ036283rc (438) ACTTTT--TGCAAA-GAGCAG--TGAACATCCCGCTCTCCTAATTCATGT

dgat2b_XM_014137863 (2584) GCCTTTGTTGTGAATGAATAGCTTGAGCAAAAT-CTGTC-TACTTAAATT

dgat2b_EG878494rc (619) GCCTTTGTTGTGAATGAATAGCTTGAGCAAAAT-CTGTC-TACTTAAATT

3001 3045

dgat2a_ XM_014197366 (2880) ACAATAAATACAGCAATA-AATAGCAGCCAA--TGCATTTTTA--

dgat2a_BQ036283rc (483) ---------------------------------------------

dgat2b_XM_014137863 (2632) AAACTTTTTATGTTTTTAGAATAAAACTTAACATGTCTTAGTAAA

dgat2b_EG878494rc (667) AAACTTTTTATGTTTTTAGAATAAAACTT----------------
