# Supplementary material for: Changes in the liver transcriptome of farmed Atlantic salmon (Salmo salar) fed experimental diets based on terrestrial alternatives to fish meal and fish oil
Source: BMC Genomics. 2018 Nov 3;19:796. doi: 10.1186/s12864-018-5188-6 (PMC6215684; doi:10.1186/s12864-018-5188-6)
Supplement: Supplementary file 5 — Figure S3. Alignment of nucleotide sequences corresponding to fabp3a and fabp3b. Conserved nucleotides in all the aligned sequences are highlighted in yellow. Fabp3a and fabp3b sequences share 92% identity over 400 aligned nucleotides. The alignment and percentage identity calculation were performed using AlignX (Vector NTI Advance 11). The nucleotide region covered by the probe C086R144 from the Agilent 44 K salmonid microarray (GEO accession number: GPL11299) is indicated within boxes. Forward qPCR primers are in bold and single underlined, whereas reverse qPCR primers are in bold and double underlined. (DOCX 22 kb) [file 12864_2018_5188_MOESM5_ESM.docx]

**Figure S3. Alignment of nucleotide sequences corresponding to *fabp3a* and *fabp3b*.**

1 50

fabp3a_NM_001123578 (1) -----------------------ATGGCTGAGGCATTCGCAGGCACATGG

fabp3b_BT050105 (1) CATTTCTCCACACTACTGCTAACATGGCTGAGGCATTCGCAGGCACATGG

51 100

fabp3a_NM_001123578 (28) AACCTGAAGGACAGCAAGAACTTTGATGAATACATGAAGGCTCTGGGTGT

fabp3b_BT050105 (51) AACCTGAAGGAGAGCAAGAACTTTGATGAATACATGAAGGCCCTGGGTGT

101 150

fabp3a_NM_001123578 (78) GGGCTTTGCGACACGCCAGGTTGGCGGTATGACCAAGCCCACCACCATCA

fabp3b_BT050105 (101) GGGCTTTGCAACCCGCCAGGTTGGCAGTATGACTA**AACCCACCACCATCA**

151 200

fabp3a_NM_001123578 (128) TCGAGGTAGCTGGAGACACAGTCACTCTGAAGACACAGAGCACTTTCAAG

fabp3b_BT050105 (151) **TTGAG**GTAGCAGGAGACATGGTCACTCTGAAGACACAGAGCACCTTCAAG

201 250

fabp3a_NM_001123578 (178) AACACCGAGATCTCCTTCAAACTAGGAGAAGAGTTCGACGAGACCACCGC

fabp3b_BT050105 (201) AACACAGAGATCAACTT**CAAACTGGGAGAGGAGTTCG**ACGAGACCACCGC

251 300

fabp3a_NM_001123578 (228) TGACGACAGGAAAGTCAAGTCCCTAATAA**CGATAGACGGTGGTAAGATG**G

fabp3b_BT050105 (251) CGACGACAGGAAGGTCAAGTCCCTAATAACGGTGGACGGTGGTAAGATGG

301 350

fabp3a_NM_001123578 (278) TTCACGTGCAAAAGTGGGACGGCAAGGAGACCACTCTGGTTCGCGAAGTC

fabp3b_BT050105 (301) TTCACGTGCAGAAGTGGGACGGCAAGGAGACCACTCTGGTCCGTGAAGTC

351 400

fabp3a_NM_001123578 (328) AGCGGCAACGCCCTCGAACGTACTCTG**ACTCTGGGTGACGTCGTCTC**CAC

fabp3b_BT050105 (351) AGCGGCAACGCCCTCGAACTGACACTGACTCTTGGTGATGTCATCTGCAC

401 450

fabp3a_NM_001123578 (378) ACGCTCCTACGTCAAGGCCGAGTGAAAGCTTATCACTATGTAACAGCCC-

fabp3b_BT050105 (401) ACGCTCCTACATCAAGGCCGAGTAACAACGTC-CGCCAAAAAAAACTCCA

451 500

fabp3a_NM_001123578 (427) --------------------------------------------------

fabp3b_BT050105 (450) CTATCCTTTACCACACCTGCCAAAATAACAACAACCCTTCACCCTTAACC

501 550

fabp3a_NM_001123578 (427) --------------------------------------------------

fabp3b_BT050105 (500) CCGATCCCCACTGTAACGACCTCTAACCCCTGTCTGACCTCTAAACCCCG

551 600

fabp3a_NM_001123578 (427) --------------------------------------------------

fabp3b_BT050105 (550) ACCCCTTCTATGTTGTCCCGTCCTTCTCTTTGTAGTGCTTCTCCTTTGTA

**C086R144**

601 650

fabp3a_NM_001123578 (427) --------------------------------------------------

fabp3b_BT050105 (600) TGAAACACTGAATAAATTCCACTGACGTTTATTTTCTTCTCTAAAGCCTT

651 700

fabp3a_NM_001123578 (427) --------------------------------------------------

fabp3b_BT050105 (650) CCTGAGTTCTTCTCTGTCAGTCACCTCACAATCCCCATGACAACAGTCAG

701 750

fabp3a_NM_001123578 (427) --------------------------------------------------

fabp3b_BT050105 (700) AACACCTACACCAGAGACTCTACTTTCATCAAACCACAGGGGTTTGCCAC

751 800

fabp3a_NM_001123578 (427) --------------------------------------------------

fabp3b_BT050105 (750) TGGCCTGGAGAAAACTCAGTGAATCTACTAACTCTGTGTAGCAACCATTT

801 850

fabp3a_NM_001123578 (427) --------------------------------------------------

fabp3b_BT050105 (800) TGTGCCAAATGGCTCATCACGTGTTACAGTTGTAGCAGCCATCTGGGTGG

851 900

fabp3a_NM_001123578 (427) --------------------------------------------------

fabp3b_BT050105 (850) GTTATGGCAGCCATTTTGTGCCAAGTTGAGATCTGAGGCTGTTGTAGGGG

901 950

fabp3a_NM_001123578 (427) --------------------------------------------------

fabp3b_BT050105 (900) AAGCAGCACATGTCTCCTCAGACGGATCAAAGCTGCTCTATATTCTGCTG

951 1000

fabp3a_NM_001123578 (427) --------------------------------------------------

fabp3b_BT050105 (950) ACAGAAAAGGGTGTGTGTAGGCAGTGCGTGTGCGTATTGTTGGATTCGGG

1001 1050

fabp3a_NM_001123578 (427) --------------------------------------------------

fabp3b_BT050105 (1000) ATAAACTTTGAACATTGAGATATTATTAAAGACATGATAGATCAGACGCA

1051 1100

fabp3a_NM_001123578 (427) --------------------------------------------------

fabp3b_BT050105 (1050) TAGTATATAAAGGATTGAGAAGGTAATGGTTCTCTGTAGTAAGACAACTA

1101 1150

fabp3a_NM_001123578 (427) --------------------------------------------------

fabp3b_BT050105 (1100) AGCTTGGAATGCATTGAGTGCAGGGAAAACAATAGCATGTGACAGTACTG

1151 1200

fabp3a_NM_001123578 (427) --------------------------------------------------

fabp3b_BT050105 (1150) ATAAGGGGAGAAACCGTTGAAGGCTAATATCACTTAGTTCCCTGCTTAGC

1201 1250

fabp3a_NM_001123578 (427) --------------------------------------------------

fabp3b_BT050105 (1200) AAGAATGATGCAATGTTTAGAGATACGGAGGAGACTCCACCAAAAGTGGG

1251 1300

fabp3a_NM_001123578 (427) --------------------------------------------------

fabp3b_BT050105 (1250) GTATGAATACTACCACGGGGGAAGCCATGTCGGTGTCTGAATTACAGCTG

1301 1339

fabp3a_NM_001123578 (427) ---------------------------------------

fabp3b_BT050105 (1300) AAACAACCCTTTGGGAAGAATTAAACGTAGATAAGATTC
