# Supplementary material for: Changes in the liver transcriptome of farmed Atlantic salmon (Salmo salar) fed experimental diets based on terrestrial alternatives to fish meal and fish oil
Source: BMC Genomics. 2018 Nov 3;19:796. doi: 10.1186/s12864-018-5188-6 (PMC6215684; doi:10.1186/s12864-018-5188-6)
Supplement: Supplementary file 6 — Figure S4. Alignment of nucleotide sequences corresponding to sgk2a and sgk2b. Conserved nucleotides in all the aligned sequences are highlighted in yellow. Sgk2a and sgk2b sequences share 92% identity over 1390 aligned nucleotides. The alignment and percentage identity calculation were performed using AlignX (Vector NTI Advance 11). The nucleotide regions covered by the probes C050R117 and C168R030 from the Agilent 44 K salmonid microarray (GEO accession number: GPL11299) is indicated within boxes. Forward qPCR primers are in bold and single underlined, whereas reverse qPCR primers are in bold and double underlined. (DOCX 33 kb) [file 12864_2018_5188_MOESM6_ESM.docx]

**Figure S4. Alignment of nucleotide sequences corresponding to *sgk2a* and *sgk2b*.**

1 50

sgk2a_NM_001139943 (1) --------------------CAACAGTGCTGTGCCCTGAGCTG-GGACAC

sgk2b_BT059352 (1) CTCTGGTCTTCTCCTTTTAGCGCAAGAAGTATGAA---AGCTCTGGAGGC

51 100

sgk2a_NM_001139943 (30) TTGACTCGATCTGCGGGG-CTTCAGTCAGTCGACAAGACATCGCTCCGGC

sgk2b_BT059352 (48) TGTAAATGCTGTACGGCCACTACAGACGGCGGATT-GACCAAGCAGAGCC

**C050R117**

101 150

sgk2a_NM_001139943 (79) TGCACGCACCTGCGACAGGTGGCCT---AAGCTTC-GTTTTAACCTTCAC

sgk2b_BT059352 (97) TATAACTAGGCTTTGCACGTTCGCTGTCACGCAGCAGTGTCAGAGAGGAA

151 200

sgk2a_NM_001139943 (125) AAAAGGTCTATGAAACACTGATGTAGGCACCCCGGTGAGCACGGAATGGA

sgk2b_BT059352 (147) AAAAGGTCTGTGAAGCACTGATGTAGGCTCCCCGGTGTGCAAGGAATGGA

201 250

sgk2a_NM_001139943 (175) CCAAGACCATGGCAAATTCTAATCCACTTAGATCACCATCCTCCACCCTC

sgk2b_BT059352 (197) CCAAGACCATGGCAAATTGTAATCCACTTAGATCACCGTCCTCCACCCTC

251 300

sgk2a_NM_001139943 (225) AGTGAGGTCAACCTGGGACCTTCAGCAAACCCACATGCCAAGCCCACTGA

sgk2b_BT059352 (247) AGTGAGGTCAACCTGGGACCCTCGGCAAACCCACATGCCAAGCCCACTGA

301 350

sgk2a_NM_001139943 (275) CTTTGACTTTTTGGCTGTTATTGGAAAAGGGACCTTTGGGAAGGTCCTGC

sgk2b_BT059352 (297) CTTTGACTTCTTGGCTGTTATTGGAAAAGGGACCTATGGGAAGGTCCTGC

351 400

sgk2a_NM_001139943 (325) TCGCCAAGCTCAAAGCTGACAATAAATTCTATGCTGTGAAAGTTCTACAG

sgk2b_BT059352 (347) TCGCCAAGCTCAAAGCTGACAACAAATTCTATGCGGTCAAAGTTTTACAG

401 450

sgk2a_NM_001139943 (375) AAGAAAGTAATCCTGAAGAAAAAGGAGCAAAAGAACATCATGGCAGAGAG

sgk2b_BT059352 (397) AAGAAAGTCATCCTGAAGAAAAAGGAGCAAAAGAACATCATGGCAGAGAG

451 500

sgk2a_NM_001139943 (425) GAACGTGCTGCTGAAGAGCCTGAAGCATCCTTTCCTGGTGGGCCTCCACT

sgk2b_BT059352 (447) GAACGTGCTGCTGAAGAGCCTGAAGCACCCTTTCCTGGTGGGCCTCCACT

501 550

sgk2a_NM_001139943 (475) ACTCCTTCCAGACCCCAGAGAAGCTCTACTTTGTCCTCGACTATGTCAAC

sgk2b_BT059352 (497) ACTCCTTCCAGACCCCAGAGAAGCTCTACTTTGTCCTGGACTATGTCAAC

551 600

sgk2a_NM_001139943 (525) GGGGGAGAGCTCTTCTTCCACCTGCAGAGGGAGCGGTGCTTCTTGGAGCC

sgk2b_BT059352 (547) GGGGGAGAGATCTTCTTCCACCTGCAGAGGGAGCGGTGCTTCTTGGAGCC

601 650

sgk2a_NM_001139943 (575) GAGGGCTCGGTTCTATGCTGCTGAGGTAGCCAGTGCCATCGGCTACCTTC

sgk2b_BT059352 (597) GAGGGCTCGGTTCTACGCTGCTGAGGTAGCCAGTGCCATCGGCTACCTTC

651 700

sgk2a_NM_001139943 (625) ACTCCCTCAACATCGTTTACAGAGATCTAAAGCCAGAGAATATTCTCTTA

sgk2b_BT059352 (647) ACTCCCTCAACATCGTTTACAGAGATCTGAAGCCAGAGAATATTCTCTTA

701 750

sgk2a_NM_001139943 (675) GACTCTCAGGGCCATGTGGTACTTACAGACTTTGGGCTGTGTAAAGAGGG

sgk2b_BT059352 (697) GACTCTCAGGGCCACGTGGTGCTTACGGACTTTGGGCTGTGTAAAGAGGG

751 800

sgk2a_NM_001139943 (725) AGTTGAGCCGGAGACCACCACGTCCACTTTCTGTGGAACCCCTGAGTATT

sgk2b_BT059352 (747) AGTGGAGCCGGATACCACCACGTCCACTTTCTGTGGAACCCCTGAGTATT

801 850

sgk2a_NM_001139943 (775) TGGCCCCTGAGGTTCTGCGTAAGGAGCCCTATGACCGCACAGTGGACTGG

sgk2b_BT059352 (797) TGGCCCCTGAGATTCTGCGTAAGGAGCCCTATGACCGCACTGTGGACTGG

851 900

sgk2a_NM_001139943 (825) TGGTGTCTGGGAGCTGTGCTCTATGAGATGATCTATAGTCTTCCCCCATT

sgk2b_BT059352 (847) TGGTGTCTAGGAGCTGTGCTCTATGAGATGATCTATAGTCTTCCTCCTTT

901 950

sgk2a_NM_001139943 (875) CTACAGCCGGGACGTGTCTGAAATGTACGATGGCATCCTACACAAGCCTC

sgk2b_BT059352 (897) TTACAGCCGGGACATGTCTGAGCTGTATGATGGTATCCTGCACAAGCCTC

951 1000

sgk2a_NM_001139943 (925) TGCCGCTGCCCCCAGGGAAGTCAGGCGCTGTCTGTAGTCTGCTCCAGGGC

sgk2b_BT059352 (947) TGCCTCTGCCCCCAGGGAAGTCAGACGCTGTCTGTAGTCTGCTCCAGAGC

1001 1050

sgk2a_NM_001139943 (975) CTCCTGCAGAAAGACCAGCACTGCAGGTTGGG**AGCCATCGCTGACTTTCT**

sgk2b_BT059352 (997) CTCCTGCAGAAGGACCAGCACTGCAGGCTGGGAGCCATCGACGACTTTTT

1051 1100

sgk2a_NM_001139943 (1025) **ACA**AATAAAGAACCATGTGTTCTTCTCCCCGATTAACTGGGATGACCTGT

sgk2b_BT059352 (1047) AGAAATCAAGAACCATGTGTTCTTCTCCCCGATTAACTGGGATGACCTGT

1101 1150

sgk2a_NM_001139943 (1075) ACCACAAGAGAATCACTCCTCCATACAACCCCAATGTGAAAGGGCCAGCG

sgk2b_BT059352 (1097) ACCACAAGCGGATCACTCCTCCGTACTACCCCAATGTGAAAGGGCCAGCG

1151 1200

sgk2a_NM_001139943 (1125) GACACACAGCACATAGACCCAGAGTTCACCAGAGAG**ATGGTGCCTAACTC**

sgk2b_BT059352 (1147) GACACGCAGCACATAGACCCAGAGTTCACCAGAGAGATGGTGCCTAACTC

1201 1250

sgk2a_NM_001139943 (1175) **AGTGGG**CCGCACCCCTGAGCTCAACGCCGGCACCAGCAGCTCTAACGCAT

sgk2b_BT059352 (1197) GGTGGGCCGCACCCCTGAGCTGAACGCCAGCACCAGCAGCAGCAACGCCT

1251 1300

sgk2a_NM_001139943 (1225) TCAATGGCTTCTCCTACGTTTCTGGTGAAGA---CAGCTTCCTTTGAGAC

sgk2b_BT059352 (1247) TCAATGGCTTCTCCTACGTCTGTGGTGAAGAAGACAGCTTCCTCTAAGAC

1301 1350

sgk2a_NM_001139943 (1272) AGGGAGGAAGGTTCCTCACAGCACCACCACTGCC-------GAGGCCGAG

sgk2b_BT059352 (1297) AGGGAAGAAGGTCCCTCACAGCACAACCACTGCCTGAGCTCGAGGTAGAA

1351 1400

sgk2a_NM_001139943 (1315) GTAGAAGTTACCCGTAACCACATATCTACAATTCGATTCCTTAACCGTAA

sgk2b_BT059352 (1347) GTAGAAGTTACCCCTAACCACATATCTAGGATTC**AATTCCTCAACCCCAA**

1401 1450

sgk2a_NM_001139943 (1365) ATCCTAACCTTAACCTTTAAGTCAGGAGGATAAAACATATATTTTACCCT

sgk2b_BT059352 (1397) **ATCC**TAACCTTAACCTCTAAGTCAGGAGG-TGAAAAAGATATTTGGCCCT

1451 1500

sgk2a_NM_001139943 (1415) GGATTCAACAGTTAGTGGCAACCTCTATCAACTTCATTGGCCACGGTCAT

sgk2b_BT059352 (1446) TGAT----CAGTTAGTGGCAACCTCGATCAACTTCATTGGCCACAGCTAT

1501 1550

sgk2a_NM_001139943 (1465) AGAAATGGAGCACATTGAATATCATGCATTTTTATGTAGGTGAAGCAA--

sgk2b_BT059352 (1492) AGAAATGGAGCACATTGAACAGAATGCATT**TTTGTGTAGGCTATGCGCAG**

1551 1600

sgk2a_NM_001139943 (1513) -GAAAGAGATGTAAG-----------------AAAACATTTAAATGCACC

sgk2b_BT059352 (1542) TGCATGCCACGTCTGTTCATCTAGACCTCATTAGAACAGGCAGACGCAGC

1601 1650

sgk2a_NM_001139943 (1545) ACCCA--ACTGTTGCTTTTG---CTGTAGGTGA----------CTGCCCT

sgk2b_BT059352 (1592) AGGCACTAATGATATCTGTGAAACTCTAGTCGAAACCATATTTCTGCTCT

1651 1700

sgk2a_NM_001139943 (1580) ATGAATTCTATTTATAT-------------GCCCTT--------------

sgk2b_BT059352 (1642) TAAAGTGCTGTTTTTAATACAAACACGGTGGACCTTCCAAACATTTGTTA

1701 1750

sgk2a_NM_001139943 (1603) ------------------GACTCTGC------------TTGATTTCTCA-

sgk2b_BT059352 (1692) TTTTAAATGAAATGTGATGATTGTCCAACAATACATTATTGATTTGAAAA

1751 1800

sgk2a_NM_001139943 (1622) -------TTTCAGGA--CAAAGTCTTTTTGCACATCCAATCGT--GCTGG

sgk2b_BT059352 (1742) CATGCTGTTTCTGTTATCACTGACTTTGTGTAGATTAGATTAGAGGCTTG

1801 1850

sgk2a_NM_001139943 (1661) ACTTTTTATTTGCTAGCAG--ATG-TTGA---------------------

sgk2b_BT059352 (1792) ACTCTATTGTTGCATCCAGGCATGCTTGAGGGGATAGCTCACTCAAATTA

1851 1900

sgk2a_NM_001139943 (1687) ----TTTACTTCTCCTTTCACTA--CAAT-GTAAAG--TCAACAGTGTGA

sgk2b_BT059352 (1842) CAAATTTCCTTTCCCTGTCAGTAGTCTATAGAAAAGGTTTGACGGCAATT

1901 1950

sgk2a_NM_001139943 (1728) CTACATAAA--GGACATGT-----AAATA-------CAGTACACGCTCCT

sgk2b_BT059352 (1892) CTAGATGATTTGAACATGCGTGGAAAATGTGCGTATCAGTACCATGACTT

1951 2000

sgk2a_NM_001139943 (1764) AGATGCTATGTGT------------AGAAGGTAAAGCATCAGGT------

sgk2b_BT059352 (1942) GAATGGGATTTGTGCCACAGGTGCCACAAAGTCATGGTACCGATATAAGC

2001 2050

sgk2a_NM_001139943 (1796) --TCTATGGAAA-GTTGTGGT-GTATGGATTAAGATGGTCTGATTATCAC

sgk2b_BT059352 (1992) AATCTATGGAAAAGGTATGACAGCAATCGGTGCTTTGGTTTTATT-TCCC

2051 2100

sgk2a_NM_001139943 (1842) TAGAACAGG-----AAGACACAAATGTGTGTGAAACTCTAGTCGAACCC-

sgk2b_BT059352 (2041) TGGCACTGTTTCTAAATGTACAAAAGCTGAAGAACATCCTCACATACAGG

2101 2150

sgk2a_NM_001139943 (1886) -ATT-------CCTCTGCTC----TTTAAGTGCTGCTTTT----AATACA

sgk2b_BT059352 (2091) TATTGTTTTTGCCACTGCTTGGAGTTAAAGGGAAACTCGTGAAAAATGTA

2151 2200

sgk2a_NM_001139943 (1920) AACACTGTGGACTT---TCCAAACATT---TCT-TAACTCGTTTGTTGAA

sgk2b_BT059352 (2141) AACACTGCACACTGCTGTTCAACCCTTAGGTCTGTTCCACAAATGCTAAA

2201 2250

sgk2a_NM_001139943 (1963) AATATAAATTTTTG---CATTGATTTGAGAATATG--CTG---------T

sgk2b_BT059352 (2191) ACCTTAGCATTTGGAGGCATTTCCAGGGGAAGTGGGACCGGAGCACGGAT

2251 2300

sgk2a_NM_001139943 (1999) TTCTATTAT--CACTGACTTTTAGTAGATTAGAGTACATAAAGGCTTGAC

sgk2b_BT059352 (2241) TGCTGTTATACCATAGACTGCTTACAGGGTAAGGAAACCAACGTGTTATT

**C168R030**

2301 2350

sgk2a_NM_001139943 (2047) T-GAAATGTTGCGTCCAGGGATGC--TTAACAGCAACATAAACCTATGCC

sgk2b_BT059352 (2291) TTGTGATGTGGGGG--AGCTATCCCTTTAACAGCAACATAAACCTACGCC

2351 2400

sgk2a_NM_001139943 (2094) AGATTATTCTCACAGCTGTAAAGGTGCGTGCCATGGTGCTATTAACCTTG

sgk2b_BT059352 (2339) A---TCTTCTTACAACTGTAAAAGTGCGTGCCATGGTGTTCTTAACCTTG

2401 2450

sgk2a_NM_001139943 (2144) TTAAACAAAACATGTGACTGTGTCAGCACAATGGGTGACATTAAAGACTG

sgk2b_BT059352 (2386) TGCAACAAGCCATATAATTGTGTCAACACGACGGGTAACATT--CGACTG

2451 2500

sgk2a_NM_001139943 (2194) TTGAC--ATGCCAACACCATCTTAAAGATGACTGACATTCAATGAGGCAC

sgk2b_BT059352 (2434) TTGACTTATGCCAGCACCGTGTTAAAGGTGACTGATGCGCAATGATG---

2501 2550

sgk2a_NM_001139943 (2242) AGTTACAAGTATGTGTACATAA---CTTCACAATAAATCACT-TCCCTAT

sgk2b_BT059352 (2481) ---------TATGTGTACATAAAAACGTTACAATAAATAACTATCCCTA-

2551 2573

sgk2a_NM_001139943 (2288) CAAAAAAAAAAAAAAAAAAAAGA

sgk2b_BT059352 (2521) -AAAAAAAAAAAAAAAAAAAAGA
