# Supplementary material for: Changes in the liver transcriptome of farmed Atlantic salmon (Salmo salar) fed experimental diets based on terrestrial alternatives to fish meal and fish oil
Source: BMC Genomics. 2018 Nov 3;19:796. doi: 10.1186/s12864-018-5188-6 (PMC6215684; doi:10.1186/s12864-018-5188-6)
Supplement: Supplementary file 7 — Figure S5. Alignment of nucleotide sequences corresponding to htra1a and htra1b. Conserved nucleotides in all three aligned sequences are highlighted in yellow, those conserved in two are highlighted in blue. The cDNA sequence named as “htra1b_assembly” was assembled using the Salmo salar EST sequences DW576053, DW556574, DW539580, EG831192, and EG831191. Htra1a and htra1b sequences share 91% identity over 1117 aligned nucleotides. The alignment and percentage identity calculation were performed using AlignX (Vector NTI Advance 11). The nucleotide regions covered by the probes C265R134, C231R170 and C170R142 from the Agilent 44 K salmonid microarray (GEO accession number: GPL11299) is indicated within boxes. Forward qPCR primers are in bold and single underlined, whereas reverse qPCR primers are in bold and double underlined. (DOCX 37 kb) [file 12864_2018_5188_MOESM7_ESM.docx]

**Figure S5. Alignment of nucleotide sequences corresponding to *htra1a* and *htra1b*.**

1 50

htra1a_NM_001141717 (1) ----------------------------------------------CAAC

htra1b_assembly (1) CCTTCAAAGGGAGTTACAGCAGCTTTGTTGTCTGACAGCAGTAGACCAAC

htra1b_EG831192 (1) --------------------------------------------------

51 100

htra1a_NM_001141717 (5) AAAGCTCATTTTAGTGAAAAGCGTCTGACATTTATTTGCAAATGTATGTA

htra1b_assembly (51) GAAGCACAGCTTAGTGAAAAGCGACTCACATTTA--TGCAACG---TTTA

htra1b_EG831192 (1) --------------------------------------------------

101 150

htra1a_NM_001141717 (55) ACGTTTATTTATGCAGATCAAACATATTTTTGTAGCAAGTCGTAAAGTTT

htra1b_assembly (96) ----TTATTTATGCAGATAAAACAT--TTTTGTAGAAAC-------GTTT

htra1b_EG831192 (1) --------------------------------------------------

151 200

htra1a_NM_001141717 (105) TAAGAGTCGTTTTTTTTGGCAACTTTGTACCTTTTTAGATACTTTAATAT

htra1b_assembly (133) GAAAATTCGTTTTT--TGACAACATTGCATAGTTTTTCA--ATTGAATAT

htra1b_EG831192 (1) --------------------------------------------------

201 250

htra1a_NM_001141717 (155) TTAACAATGTTTTGGTGCGTCTTCTGCGCAACTTTTATTCTTGCTCCTTT

htra1b_assembly (179) TTAACAATGTTTTGGTCGGTCATCTGCGCAACTTTTCTCCTTGCTCCTTT

htra1b_EG831192 (1) --------------------------------------------------

**C265R134**

251 300

htra1a_NM_001141717 (205) AGTTTGCGAGTCAAGAGCCAAGCGATATGTCATCGGCTGTCCAGATAAAT

htra1b_assembly (229) AGTTTGCGAGGCAAGAACCAAGCGATATGTCATCGGCTGTCCAGATAAAT

htra1b_EG831192 (1) --------------------------------------------------

301 350

htra1a_NM_001141717 (255) GTGACAAATTTCTATGTCCCCCGATCCCTGCGGACTGTTTGGCCGGCGAC

htra1b_assembly (279) GTGACAAATCTCTATGTCCCCCGATCCCTGCGGACTGCATGGCCGGCGAC

htra1b_EG831192 (1) --------------------------------------------------

351 400

htra1a_NM_001141717 (305) ATCCTTGACCAATGCGACTGCTGTCCGGTCTGTGCGCACGGAGAAGGTGA

htra1b_assembly (329) ATACTTGACCAATGCGACTGTTGTCCGGTCTGTGCGTCCGAAGAGGGTGA

htra1b_EG831192 (1) --------------------------------------------------

401 450

htra1a_NM_001141717 (355) GGTGTGCGGCGGCACGGGGAGACTAGGGGACCCGGAGTGCGGAGAGGGCA

htra1b_assembly (379) GGCATGCGGCGGCACAGGGAGATTAGGGGACCCGGAGTGCGGAGAGGGCA

htra1b_EG831192 (1) --------------------------------------------------

451 500

htra1a_NM_001141717 (405) TGGACTGCTCGATATCGGACGGAATTGGGGTGTCCGCCACAGTAAGGCGT

htra1b_assembly (429) TGGAATGCTCGATAACGGACGGCATTGGGGTGTCCGCCACTGTGAGGCGT

htra1b_EG831192 (1) --------------------------------------------------

501 550

htra1a_NM_001141717 (455) CGGGGCAAAAACGGTGTGTGCGTCTGCAAAGTTGCGGACCCGGTGTGCGG

htra1b_assembly (479) CGGGGCAAAAGCGGTGTGTGCGTCTGCAAAGGTTCGGACCCGGTGTGCGG

htra1b_EG831192 (1) --------------------------------------------------

551 600

htra1a_NM_001141717 (505) CAGTGACGGGGTGTCCTACCGAAACATCTGCGAACTGAAGAGATTGAGTA

htra1b_assembly (529) CAGTGACGGGGTGTCCTACCAAAACATCTGCGAACTGAAGAGAGTGAGTA

htra1b_EG831192 (1) --------------------------------------------------

601 650

htra1a_NM_001141717 (555) ACCGGGCTCTGAAGCTTCAGCAGCCACCGGTCATCTTCATACAGAGAGGA

htra1b_assembly (579) ACCGGGCTCTGAAGCTGCAGCAGCCACCGGTCATCTTCATACAGAGAGGA

htra1b_EG831192 (1) --------------------------------------------------

651 700

htra1a_NM_001141717 (605) ACCTGTAGCAAAGGCCAGGAGAATCCAGACAGTCTGCGCCACAGATATAA

htra1b_assembly (629) ACCTGTGGCAAAGGCCAGGAGAATCCAGACAGTCTGCGCCACAGATACAA

htra1b_EG831192 (1) --------------------------------------------------

701 750

htra1a_NM_001141717 (655) CTTCATC**GCTGATGTGGTGGAGGAGAT**CGCTCCCGCTGTGGTTCATATTG

htra1b_assembly (679) CTTTATTGCTGACGTGGTTGAGAAGATTGCTCCTGCTGTGGTTCATATTG

htra1b_EG831192 (1) --------------------------------------------------

751 800

htra1a_NM_001141717 (705) AACTTTACCGCAAGATGGTGTTCTCTAAGCGTGAGGTGGCGGTGGCCAGC

htra1b_assembly (729) AACTCTACCGCAAGATGGTATTCTCTAAGCGGGAGGTGGCGGTGGCCAGC

htra1b_EG831192 (1) --------------------------------------------------

801 850

htra1a_NM_001141717 (755) GGGTCTGGCTTCGTG**GTGTCAGAGGACGGCTTGA**TTGTGACCAACGCCCA

htra1b_assembly (779) GGTTCTGGCTTTGTGGTGTCAGAGGACGGCCTGATCGTGACCAACGCCCA

htra1b_EG831192 (1) --------------------------------------------------

851 900

htra1a_NM_001141717 (805) CGTGGTGGCCAATAAGCACCGGGTGAAGGTGGAGCTGAAGAGTGGCGCCA

htra1b_assembly (829) CGTGGTGGCCAATAAGCACCGGGTGAAGGTGGAGCTGAAGAGTGGCGCTA

htra1b_EG831192 (1) --------------------------------------------------

901 950

htra1a_NM_001141717 (855) CCTTCGACGCCAAGATCACAGACGTGGACGAGAAGGCAGACATTGCCCTC

htra1b_assembly (879) CCTTCGACGCCAAGATCAAAGACGTGGACGAGAAGGCCGACATCGCCCTC

htra1b_EG831192 (1) --------------------------------------------------

951 1000

htra1a_NM_001141717 (905) ATCAAGATCGACACCCCGATGAAACTGCCGGTGCTGCTGCTGGGTCGTTC

htra1b_assembly (929) ATCAAGATCGACGTCCCGATGAAGCTGCCGGTGCTGCTGCTGGGTCGTTC

htra1b_EG831192 (1) --------------------------------------------------

1001 1050

htra1a_NM_001141717 (955) AGCTGACCTGAGGCCTGGTGAGTTTGTTGTGGCCATCGGCAGCCCCTTCT

htra1b_assembly (979) AGCTGACCTGAGGCCTGGTGAGTTTGTGGTGGCCATCGGCAGCCCCTTCT

htra1b_EG831192 (1) ---------------GGGGGAGTTTGTGGTGGCCATCGGCAGCCCCTTCT

1051 1100

htra1a_NM_001141717 (1005) CCCTGCAGAACACGGTCACCACAGGTATCGTCAGCACCACCCAAAGAGGA

htra1b_assembly (1029) CCCTGCAGAACACGGTCACCACAGGTATCGTCAGCACCACCCAGAGAGGA

htra1b_EG831192 (36) CCCTGCAGAACACGGTCACCACAGGTATCGTCAGCACCACCCAGAGAGGA

1101 1150

htra1a_NM_001141717 (1055) GGCAAGGAGCTTGGCCTGAGGAACTCTGATATGGAATACATCCAGACGGA

htra1b_assembly (1079) GGCAAGGAGCTGGGCCTGAGGAACTCTGATATGGACTACATCCAGACGGA

htra1b_EG831192 (86) GGCAAGGAGCTGGGCCTGAGGAACTCTGATATGGACTACATCCAGACGGA

1151 1200

htra1a_NM_001141717 (1105) CGCTATCATCAACGAAGGAAGTGTAGATCTACCATCTGATCATCCCTTTA

htra1b_assembly (1129) CGCTATCATCAAC--------------------------T----------

htra1b_EG831192 (136) CGCTATCATCAAC--------------------------T----------

1201 1250

htra1a_NM_001141717 (1155) ACCTGAATGCTGTTCCTTTTCAGTATGGGAACTCTGGCGGACCCCTGGTC

htra1b_assembly (1143) ------------------------ATGGGAACTCTGGCGGACCCCTGGTC

htra1b_EG831192 (150) ------------------------ATGGGAACTCTGGCGGACCCCTGGTC

1251 1300

htra1a_NM_001141717 (1205) AATCTGGACGGAGAGGTGATTGGGATCAACACACTGAAGGTGACAGCAGG

htra1b_assembly (1169) AATCTGGATGGAGAGGTGATTGGGATCAACACACTGAAGGTGACAGCAGG

htra1b_EG831192 (176) AATCTGGATGGAGAGGTGATTGGGATCAACACACTGAAGGTGACAGCAGG

1301 1350

htra1a_NM_001141717 (1255) AATCTCCTTCGCCATCCCCTCAGACAAGATCCGTCAGTTCTTGGCAGAGT

htra1b_assembly (1219) AATCTCCTTCGCCATCCCCTCAGACAAGATCCGTCAGTTCCTGGCAGAGT

htra1b_EG831192 (226) AATCTCCTTCGCCATCCCCTCAGACAAGATCCGTCAGTTCCTGGCAGAGT

1351 1400

htra1a_NM_001141717 (1305) CCCACGGCAGACAATCTAAAGGTAGA---------------------TTA

htra1b_assembly (1269) CCCATGACAGGCAATCTAAAGGTACAGATTACCAATCTAAAGGGAAATTA

htra1b_EG831192 (276) CCCATGACAGGCAATCTAAAGGTACAGATTACCAATCTAAAGGGAAATTA

1401 1450

htra1a_NM_001141717 (1334) TTACCAAAGAAGAAGTATATCGGTGTGAGGATGATGACTCTCACTACAAC

htra1b_assembly (1319) TCAACAAGTAAGAAATATATCGGTGTGAGGATGATGACTCTCACACCAAT

htra1b_EG831192 (326) TCAACAAGTAAGAAATATATCGGTGTGAGG**ATGATGACTCTCACACCAAT**

1451 1500

htra1a_NM_001141717 (1384) GCTTGCAAAGGAGCTGAAGGAGAGAACATCAGACTTCCCTGATGTTACCT

htra1b_assembly (1369) GCTTGCAAAGGAGCTGAAGGAGAGACAATCAGACTTCCCCGATGTTACCT

htra1b_EG831192 (376) **GC**TTGCAAAGGAGCTGAAGGAGAGACAATCAGACTTCCCCGATGTTACCT

1501 1550

htra1a_NM_001141717 (1434) CAGGGGCATATGTCATCGAGGTCATCCCAAAAACACCAGCTGAGACAGGT

htra1b_assembly (1419) CAGGGGCATATGTAATCGAGGTCATCCCAAAAACACCAGCTGAGACAGGT

htra1b_EG831192 (426) CAGGGGCATATGT**AATCGAGGTCATCCCAAAAAC**ACCAGCTGAGACAGGT

1551 1600

htra1a_NM_001141717 (1484) GGCCTGCAGGAGAGTGACGTCATAATCACCATCAACAGCCAGCGAATCAC

htra1b_assembly (1469) GGCCTGCAGGAGAGTGATGTCATAATCACCATCAACAGCCAGCGAATCAC

htra1b_EG831192 (476) GGCCTGCAGGAGAGTGATGTCATAATCACCATCAACAGCCAGCGAATCAC

1601 1650

htra1a_NM_001141717 (1534) CTCGGCCAGTGATGTCAGCAGCTCTATCAAGAGGGACGACACGCTGCGAA

htra1b_assembly (1519) CTCAGCCAGTGACGTCAGCAGCTCCATCAAGAGGGAAGACACGCTGCGCA

htra1b_EG831192 (526) CTCAGCCAGTGACGTCAGCAGCTCCATCAAGAGGGAAGACACGCTGCGCA

1651 1700

htra1a_NM_001141717 (1584) TGGTGGTCCGGCGGGGGAACGAGGACATCATGCTCACCGTCGTCCCCGAG

htra1b_assembly (1569) TGGTGGTCCGGCGGGGGAACGAGGACGTCATGCTCACCGTCGTCCCAGAG

htra1b_EG831192 (576) TGGTGGTCCGGCGGGGGAACGAGGACGTCATGCTCACCGTCGTCCCAGAG

1701 1750

htra1a_NM_001141717 (1634) GACATTGACCCTTGACCTCTCAGCAACCACGAGCTGGTTCTCAGTGTTTA

htra1b_assembly (1619) GAGATTGACCCTTGACCTCTCAGCAACCACGAGCTGGTTCTCAGTGTTGA

htra1b_EG831192 (626) GAGATTGACCCTTGACCTCTCAGCAACCACGAGCTGGTTCTCAGTGTTGA

1751 1800

htra1a_NM_001141717 (1684) AAACCACGGAC-TTAAACCGGTTGTGTGTCTGGATCCACACCCTACCATA

htra1b_assembly (1669) AAATCACGGACCTTAAACCGG--GTGTGTCTGGAGCCACACCCTACCTTA

htra1b_EG831192 (676) AAATCACGGACCTTAAACCGG--GTGTGTCTGGAGCCACACCCTACCTTA

1801 1850

htra1a_NM_001141717 (1733) GAACCTGTAACTACGGGTG----CTCTTCGCTCTCAGTGAA--ACTGATT

htra1b_assembly (1717) GAACCTGTAACTACTAGTGTCTCCCCCCCACTCCCCTCAAACTACAGAGG

htra1b_EG831192 (724) GAACCTGTAACTACTAGTGTCTCCCCCCCACTCCCCTCAAACTACAGAGG

**C170R142**

**C231R170**

1851 1900

htra1a_NM_001141717 (1777) ATTGTTTTATACTCTACTAACGTCCTTGCA-CAGAAAACCCACCTCAATC

htra1b_assembly (1767) TCTGTGAGGCCCACAACTAACCCTCTGGGAGCTCAAATGTGTCCAGGGTA

htra1b_EG831192 (774) TCTGTGAGGCCCACAACTAACCCTCTGGGAGCTCAAATGTGT--------

1901 1950

htra1a_NM_001141717 (1826) AGATAGAAAAGTGGGTGTCACAGATTACCGTAGTTTTG---TAGTTGTTT

htra1b_assembly (1817) ATAAAGACATATTTTAG-CACCCCTGCTAGTTTTTTTGGATTATTTGTCC

htra1b_EG831192 (816) --------------------------------------------------

1951 2000

htra1a_NM_001141717 (1873) T--TGTTGCTGTTGATATTATGTTTTTTTTTT-ATTG--------TATTT

htra1b_assembly (1866) TCATGTATGAGTCAAAAAGATACTTCTACACTCATTGAAACTGATTATTG

htra1b_EG831192 (816) --------------------------------------------------

2001 2050

htra1a_NM_001141717 (1912) TAATTGCATTTCTCTTGATACGTTTTGACGACAATTTCACAGAGGGAATA

htra1b_assembly (1916) CCATCACGTTTGTTTTTATACTCTATTAACATCCTTGCACAGAGA-ACCC

htra1b_EG831192 (816) --------------------------------------------------

2051 2100

htra1a_NM_001141717 (1962) AAAAGATTTTGAAAAAAAAAAAAAAAAAAAAAAAAAGA------------

htra1b_assembly (1965) TCCTCAGTCAGAGAGAAGAAGTGAAGGGTGTCCTTAATCTGGTTTGTTGT

htra1b_EG831192 (816) --------------------------------------------------

2101 2150

htra1a_NM_001141717 (2000) --------------------------------------------------

htra1b_assembly (2015) TGATATTGTTGATGTTTTTAGTTGTATTTCTCTTGGTATGTTTTGACCAC

htra1b_EG831192 (816) --------------------------------------------------

2151 2177

htra1a_NM_001141717 (2000) ---------------------------

htra1b_assembly (2065) AATTTCACAGAGGGAATAAAATGGTTT

htra1b_EG831192 (816) ---------------------------
