# Supplementary material for: Changes in the liver transcriptome of farmed Atlantic salmon (Salmo salar) fed experimental diets based on terrestrial alternatives to fish meal and fish oil
Source: BMC Genomics. 2018 Nov 3;19:796. doi: 10.1186/s12864-018-5188-6 (PMC6215684; doi:10.1186/s12864-018-5188-6)
Supplement: Supplementary file 8 — Figure S6. Alignment of nucleotide sequences corresponding to gadd45ba and gadd45bb. Conserved nucleotides in all the aligned sequences are highlighted in yellow. “Rc” after the GenBank accession numbers stands for reverse complement. Gadd45ba and gadd45bb sequences share 78% identity over 767 aligned nucleotides. The alignment and percentage identity calculation were performed using AlignX (Vector NTI Advance 11). The nucleotide regions covered by the probe C100R113 from the Agilent 44 K salmonid microarray (GEO accession number: GPL11299) is indicated within a box. Forward qPCR primers are in bold and single underlined, whereas reverse qPCR primers are in bold and double underlined. (DOCX 20 kb) [file 12864_2018_5188_MOESM8_ESM.docx]

**Figure S6. Alignment of nucleotide sequences corresponding to *gadd45ba* and *gadd45bb*.**

1 50

gadd45ba_BT047350 (1) GGGGGAGAAACAACTGAGCCACAGTCAGAGCTAATAGAAGGACTTTGCAA

gadd45bb_EG900267rc (1) --------------------------------------------------

51 100

gadd45ba_BT047350 (51) TTCAACAGCGAGCGAGTTTACGCTGAAACATTGCAAGGGAACCTATACCG

gadd45bb_EG900267rc (1) --------------------------------------------------

101 150

gadd45ba_BT047350 (101) TATTGGATTACTGATTTGCCGGACGCGCTGGAAACCTCGATTCGGGAATA

gadd45bb_EG900267rc (1) --------------------------------------------------

151 200

gadd45ba_BT047350 (151) TACTCATTGGAGATTATCCTTGTGGATTATACAATTAACGAACTTCAATA

gadd45bb_EG900267rc (1) --------------------------------------------------

201 250

gadd45ba_BT047350 (201) TGACTCTGGAAGAACTGGGATGCAATATCACTGAGAAAAAGATGGAGACC

gadd45bb_EG900267rc (1) --------------------------------------------------

251 300

gadd45ba_BT047350 (251) GTGAGTCAAGCTCTAGAAGAGCTGCTGGTGGCAGCGCAGCAACAAGACTG

gadd45bb_EG900267rc (1) --------------------------------------------------

301 350

gadd45ba_BT047350 (301) CCTGACTTTGGGAGTCTACGAGTCTGCAAAACTGATGAATGTTGATCCTG

gadd45bb_EG900267rc (1) --------------------------------------------------

351 400

gadd45ba_BT047350 (351) ATAGTGTAGTCTTGTGTGTTCTGGCGACTGATGAGGAAGACGAGGATGAC

gadd45bb_EG900267rc (1) --------------------------------------------------

401 450

gadd45ba_BT047350 (401) ATCGCACTGCAGATTCACTTCACGCTCATCCAAGCCTTCTGCTGCGACAA

gadd45bb_EG900267rc (1) --------------------------------------------------

451 500

gadd45ba_BT047350 (451) CGACATCAACATACTGAGAGTCTCGGGCATCAGGCGCCTCGCTCAGGTTC

gadd45bb_EG900267rc (1) --------------------------------------------------

501 550

gadd45ba_BT047350 (501) TTGGCGAGCCAAGCACCGCTGACAGCAACGGCAACGAGCCCAAAGATCTG

gadd45bb_EG900267rc (1) --------------------------------------------------

551 600

gadd45ba_BT047350 (551) CACTGCATCCTTGTCACTAACACCCAGTGCCAATCTCTGAAATGCCAAGC

gadd45bb_EG900267rc (1) --------------------------------------------------

601 650

gadd45ba_BT047350 (601) GTTGCAGGACGTGGGCAACTACTGCGAGGAGAGCCGCTGCAAGAACCAGT

gadd45bb_EG900267rc (1) --------------------------------------------------

651 700

gadd45ba_BT047350 (651) GGGTGCCTTATCTGGCCCTGCAGGAGCGCTGAACTAACGACCCTCACTGA

gadd45bb_EG900267rc (1) --------------------------------------------------

701 750

gadd45ba_BT047350 (701) GAAGCGTGAAATAAACAAGTCGTGAAACTAAACAAGTCGTCATTCTTCAA

gadd45bb_EG900267rc (1) -----------AAAGCGATTCAAATAAGTAAACAAGTCGTCATTCTTGAA

751 800

gadd45ba_BT047350 (751) GAATGAGAAAGGCATTCAAG---GGGCATTATCGATAGGCCTGTGTCTGC

gadd45bb_EG900267rc (40) GAATGAGAAAGGCGTTCAAGAAGGGGCATCGTCGACATGCCTGTGTTTGC

801 850

gadd45ba_BT047350 (798) CTACCCCTTGCCGCTACCCAGTGACGGAGCTGGGAATGCGCATGTTCCAA

gadd45bb_EG900267rc (90) CTGCCCTGGTCCGCTATCCAGTGACGGAGTTGAGCATGCGAAGATTCCGA

851 900

gadd45ba_BT047350 (848) GGGACAATGCACATGCATCCATAACGCATCGGGCCCCAGTTCTCTTCCTC

gadd45bb_EG900267rc (140) TGGACAATGCGCATACAACCATGACGCATCTGACCCC-GCTCTCTTCCTT

901 950

gadd45ba_BT047350 (898) TGCGGAAAGGAAAAGTATCCGCTGGAGTGGGGAGTAAAAACGAACGGTAG

gadd45bb_EG900267rc (189) CGCGGAAAGGAACAGTGTCGGCCGGAGTGGGGCGTAGGAACGAACTGTAG

951 1000

gadd45ba_BT047350 (948) AGAAGACTCACTGCTGTTTGCCCAGCCATTTTGGAGCAACCGTGGGCGGC

gadd45bb_EG900267rc (239) AGAAATCTCGCTGCTGTTTGCCCAGCCATGTTGGAACAACCGGGGGCGGC

1001 1050

gadd45ba_BT047350 (998) TGCATAGCGTGGGAACTGATTTGCAGTTTCGTTTATGCAGAGGAGGGACA

gadd45bb_EG900267rc (289) TGCATCGCGTGGGAACGCACTTGCAGTTTCGTTATTTGA**GAGGAGGGACA**

1051 1100

gadd45ba_BT047350 (1048) AAGCAACAG--------AGAATACAGACTTGAAACTGGGTTTAACCCTAT

gadd45bb_EG900267rc (339) **AAGCAACTG**TATCAATGAGAATACAGACTTGAAACTGAGTTTAACCCTAT

1101 1150

gadd45ba_BT047350 (1090) GCTTTCCGACGTGGAAGTCGGGGTATTTATGATGAAATGCAATGTGAATG

gadd45bb_EG900267rc (389) GCTTTCTGACGTGGAAGTCAGTGTGTTTATGATCAAATGCAATGTGAATG

1151 1200

gadd45ba_BT047350 (1140) ACTTCAATGCTCTATGGTTGGATGTTATGAG--------CTTTGAAGAAG

gadd45bb_EG900267rc (439) ACTGCAATGCTCTACA**GTTGGATGTTATGAGTAACAGTGCA**ATGAAGAAG

1201 1250

gadd45ba_BT047350 (1182) TGTCTTCAGAGCAACAATGCCTATGGATTGTTATTATGTGAAATATATTG

gadd45bb_EG900267rc (489) T--CTTCAGAGCAACAATGCCTATGGGTTGTTATTGTGTAAAAGATATTG

1251 1300

gadd45ba_BT047350 (1232) ATCAGGTATTCTG-------------------------GCTACACTCATA

gadd45bb_EG900267rc (537) ATAGGGTATTGTGTAAAAGATATTGATAGGGTATTGTGGTTACACTCATA

1301 1350

gadd45ba_BT047350 (1257) GCC**CGATGGCTGTCAGTTAAGAGG**GTCAAAACTTTG--------CCACAA

gadd45bb_EG900267rc (587) GCCAGGTGGCTGCCTGTTAAGAGGGTCAAAACAATGTAACAATGCCACAA

1351 1400

gadd45ba_BT047350 (1299) TGTAACAATGTATT--------TGACATTGACACATCAAACTAATGTATT

gadd45bb_EG900267rc (637) TGTAACAATGTATTCATTTATTTGACATTGACACAATTAACTAATGTATT

**C100R113**

1401 1450

gadd45ba_BT047350 (1341) GTTATTATTTAAGTTGGACCGAGTAATATATGACA**GTGAACTAACTGCAT**

gadd45bb_EG900267rc (687) GCTATTATTTAAGTTGAACAGAGTAATATATAACAGTGAAAT----GCAT

1451 1494

gadd45ba_BT047350 (1391) **TGCTGAATT**TAATGTTAAACCTATTTGAATAAATTATTGAACC-

gadd45bb_EG900267rc (733) ---TG---------TTAAACCTATTTGAATAAATTATTGAACTG
