# Supplementary material for: Changes in the liver transcriptome of farmed Atlantic salmon (Salmo salar) fed experimental diets based on terrestrial alternatives to fish meal and fish oil
Source: BMC Genomics. 2018 Nov 3;19:796. doi: 10.1186/s12864-018-5188-6 (PMC6215684; doi:10.1186/s12864-018-5188-6)
Supplement: Supplementary file 9 — Figure S7. Alignment of nucleotide sequences corresponding to lect2a and lect2b. Conserved nucleotides in all the aligned sequences are highlighted in yellow. Lect2a and lect2b sequences share 88% identity over 531 aligned nucleotides. The alignment and percentage identity calculation were performed using AlignX (Vector NTI Advance 11). The nucleotide regions covered by the probes C134R121, C164R142 and C159R112 from the Agilent 44 K salmonid microarray (GEO accession number: GPL11299) is indicated within boxes. Forward qPCR primers are in bold and single underlined, whereas reverse qPCR primers are in bold and double underlined. (DOCX 29 kb) [file 12864_2018_5188_MOESM9_ESM.docx]

**Figure S7. Alignment of nucleotide sequences corresponding to *lect2a* and *lect2b*.**

1 50

lect2a_BT059281 (1) -CAAGACTTTCTTGTAGATTTTCTCCTTGA-GTGAAAGCATCATCTAAGG

lect2b_DV106130 (1) GCAAGACCTTCTTGCAGAGTTTTTCCTTGAAGCGAAAGCATCCTCTCAGG

**C134R121**

**C164R142**

51 100

lect2a_BT059281 (49) CTTTACCATGAGGACTGCTGTTCTTTTGTTTACTGTGGTGCTCATAGCTG

lect2b_DV106130 (51) CTTCAACATGAAGACAGCTATTCTTCTGCTTACTGTAGTCCTTATGGCTG

101 150

lect2a_BT059281 (99) TGTTGTCAGAGTGCGAGATGGTCAAGTTTGGTCAGCTGTGCAGCGGCAAC

lect2b_DV106130 (101) TGTTGCCAGAGTGTGAGATGGCCAAGTTTGGTCAGCTGTGTAGCGGAAAT

151 200

lect2a_BT059281 (149) TCCAGTAACAGGAGGAGGACAGGGGA**CAGATGGGGACAAGGACACT**ACGG

lect2b_DV106130 (151) TCCAGTAACAGGAGGAGGACAGGGG**ACAACTGGGGACAAGGACAG**TACGG

201 250

lect2a_BT059281 (199) CGCACGCAGAGGAAACCGTGAGCATCAGGGCCTGGACATTGTGTGTAATG

lect2b_DV106130 (201) AGCAAGCAGAACAGACCATGTGCATAAGGGCATTGACATCGTGTGTAACG

251 300

lect2a_BT059281 (249) ATGGGGCCACAGTGTACGCTCCATTTGATGTGAAACTCAATGGGAAAGTG

lect2b_DV106130 (251) ACGGGGCCACAGTGTACGCTCCATTTGACG**TGAAACTCAACGGCAAAGTG**

301 350

lect2a_BT059281 (299) ATCGTG**TACACAGACCCGAAGAAGGC**AGCCATCAATGATGGGATCAACCT

lect2b_DV106130 (301) ACAGTGTACACAAACCCAAAGAAAGCAGCCATCAACGATGGGATCAACCT

351 400

lect2a_BT059281 (349) CAGTGGAGAGGGTCTGTGCTTTAAGCTGTTCTACGTAAAGCCTGACAAGT

lect2b_DV106130 (351) CAGTGGGGAAGGTCTGTGCTTTAAGCTGTTCTACGTGAAGCCTGACAGTT

401 450

lect2a_BT059281 (399) ACTCTGGGGTGGTGAAGAAGGGCCAGAGGATTGGGACCCTGCTGACCATG

lect2b_DV106130 (401) ACTCTGGGGTGGTGAAGAAGGGCCAGAGGATTGGGACCCTGCTCCCCATG

451 500

lect2a_BT059281 (449) CAAAGTGTCTACCCAGGGATCACTTCTCACGTCCACGTCCAGATGTGTGA

lect2b_DV106130 (451) CAGAGTGTCTACCCAGGGATCACTTCTCACGTCCACGTCCAGATGTGTGA

501 550

lect2a_BT059281 (499) CAAGTCTGACCCCACCAAGTTTTTCTAATGGAGTCCCCTTTGGCCTCTCC

lect2b_DV106130 (501) CAAGTCCGACCCCACCAAGTACTTCTGATTGA-------TTGG---TTAA

551 600

lect2a_BT059281 (549) ATCAATCAATCAATCAATCAATCAATCAATCAATCAATCAATCAATCAAT

lect2b_DV106130 (541) ATAAATCATCTAAATTATCAC-CAATG-----------------------

601 650

lect2a_BT059281 (599) CAATCAATCAATCAATCAATCAATCAATCAATCAATCAATCTACAAAATG

lect2b_DV106130 (567) --------------------------------------------------

651 700

lect2a_BT059281 (649) ATTGTAATCATTGGCCAATAGATGGGCTTACTGTGTTTAAAAATAATAAT

lect2b_DV106130 (567) --------------------------------------------------

701 750

lect2a_BT059281 (699) TTGCTTATTATAATAAACATTTTTTATTACAGTATAAAATACATAAAAGT

lect2b_DV106130 (567) --------------------------------------------------

751 800

lect2a_BT059281 (749) TGCACACTTCTGGAATAAAGTTTTAACCCTTTAAGCATCAACAGGGGTAA

lect2b_DV106130 (567) --------------------------------------------------

801 850

lect2a_BT059281 (799) TTCATAATTTCAAAAACCTTTTTTTTATCCTTTTATGCAACTAGTTACAG

lect2b_DV106130 (567) --------------------------------------------------

851 900

lect2a_BT059281 (849) ACATGAAAATAATTTACAATGATTTCTGTGTTAATATATAATTTCTGTGG

lect2b_DV106130 (567) --------------------------------------------------

901 950

lect2a_BT059281 (899) TATATATATATATATATATATATATATATATATATATATATATATATATA

lect2b_DV106130 (567) --------------------------------------------------

951 1000

lect2a_BT059281 (949) TAAAATCTCTTTTTTGTGTACTAGTTTTACGTGGCTAATCTGACAATGTG

lect2b_DV106130 (567) --------------------------------------------------

1001 1050

lect2a_BT059281 (999) GACTCAATAGTAAACTTTGAAAAAAACATAGATTTAAAACAAATACACGT

lect2b_DV106130 (567) --------------------------------------------------

1051 1100

lect2a_BT059281 (1049) GTCAAGGCTGTGGGTAACTGGTGAAAGGAGTCAGGCGCAGGAGAGCTGAG

lect2b_DV106130 (567) --------------------------------------------------

1101 1150

lect2a_BT059281 (1099) ATGCGTGGACAAGGTATTTAATACAAGAAAACATCAGTATGAACACAATA

lect2b_DV106130 (567) --------------------------------------------------

1151 1200

lect2a_BT059281 (1149) CTATGGTGCTGGAAAAAAAACGGTACCACGAAATTAACAGGCGTAATAAA

lect2b_DV106130 (567) --------------------------------------------------

1201 1250

lect2a_BT059281 (1199) AAAACCCGGTAACAATATACCAGCCGTCAGATACAGCCTTACAATAAAAC

lect2b_DV106130 (567) --------------------------------------------------

1251 1300

lect2a_BT059281 (1249) AAAGACGCACACAAACATCGGGGAAACCAGAGGGTTAAATAATGAACATG

lect2b_DV106130 (567) --------------------------------------------------

1301 1350

lect2a_BT059281 (1299) TAATGGGGGAATTGAAACCAGGTGTGTAAAAAAACAACAACAAAACAAAT

lect2b_DV106130 (567) --------------------------------------------------

1351 1400

lect2a_BT059281 (1349) GGAAAATGAAAAGTGGATCGGTGATGGCTACCGCCGAATGCCGCTCGAAC

lect2b_DV106130 (567) --------------------------------------------------

1401 1450

lect2a_BT059281 (1399) AAGGAGCGGGACCGACTCCGGCGGAAGTCGTGACAACATGTGTGTCATTG

lect2b_DV106130 (567) --------------------------------------------------

1451 1500

lect2a_BT059281 (1449) AACAACCCCATACCATGACTAGATGGTGGAAAAAAACACCCATCTCTTTA

lect2b_DV106130 (567) --------------------------------------------------

1501 1550

lect2a_BT059281 (1499) ATAAGTTCTTTAGACAATTAAAGCTAATTTACCAACATTTCTGAAAATTT

lect2b_DV106130 (567) --------------------------------------------------

1551 1600

lect2a_BT059281 (1549) GGAATGAAACTTCTATTGATAAAAATAACTGTTTAAAACATGAAAAACAT

lect2b_DV106130 (567) --------------------------------------------------

1601 1650

lect2a_BT059281 (1599) ACTAAAATGTATATTTTTAAACATGAAATTTGACTATAAATGTGTGAAAA

lect2b_DV106130 (567) --------------------------------------------------

1651 1700

lect2a_BT059281 (1649) TTTAAAATATATGCATATTTTGGGGAATTTAATAAAAGAAAAAGTGTTAC

lect2b_DV106130 (567) --------------------------------------------------

1701 1750

lect2a_BT059281 (1699) TGTTTGACAGAAAATGCCATTTTGCCATTATTTCCAATTTGGGGTTAAAA

lect2b_DV106130 (567) --------------------------------------------------

**C159R112**

1751 1800

lect2a_BT059281 (1749) TGACCCATGTTGATGCATTTAGGGGTCAAAATATGTTGGTCCTTTTGGGG

lect2b_DV106130 (567) --------------------------------------------------

1801 1850

lect2a_BT059281 (1799) TATTAACAACAAAATAAATACATCATAAATCATTCCTAATAAATAATGTG

lect2b_DV106130 (567) --------------------------------------------------

1851 1900

lect2a_BT059281 (1849) TATTTTTGTAAAAAAAAAAAAAAAAAAAAAAAAAAAAAAAAAAAAAAAAA

lect2b_DV106130 (567) --------------------------------------------------

1901 1914

lect2a_BT059281 (1899) AAAAAAAAAAAAGA

lect2b_DV106130 (567) --------------
