# Supplementary material for: Changes in the liver transcriptome of farmed Atlantic salmon (Salmo salar) fed experimental diets based on terrestrial alternatives to fish meal and fish oil
Source: BMC Genomics. 2018 Nov 3;19:796. doi: 10.1186/s12864-018-5188-6 (PMC6215684; doi:10.1186/s12864-018-5188-6)
Supplement: Supplementary file 10 — Figure S8. Alignment of nucleotide sequences corresponding to igma and igmb. Conserved nucleotides in all the aligned sequences are highlighted in yellow. Igma and igmb sequences share 96% identity over 1548 aligned nucleotides. The alignment and percentage identity calculation were performed using AlignX (Vector NTI Advance 11). The nucleotide regions covered by the probes C134R121, C164R142 and C159R112 from the Agilent 44 K salmonid microarray (GEO accession number: GPL11299) is indicated within boxes. Forward qPCR primers are in bold and single underlined, whereas reverse qPCR primers are in bold and double underlined. (DOCX 32 kb) [file 12864_2018_5188_MOESM10_ESM.docx]

**Figure S8. Alignment of nucleotide sequences corresponding to *igma* and *igmb*.**

1 50

igma_BT058702 (1) --------------------------------------------------

igmb_BT059185 (1) CTTTTACAGAATTTTAGAACTTTTTCACAATGAGAGTTGTAGAATGTGTG

51 100

igma_BT058702 (1) --------------------------------------------------

igmb_BT059185 (51) TTCCTCCTGGCTCTCATCTCAGCTGTTCAGGGGCAGTCACTCACCTCTTC

101 150

igma_BT058702 (1) --------------------------------------------------

igmb_BT059185 (101) TGAGCCATTAGTGAAGAGCCCTGGAGAGTCAGTAACACTGTACTGTACTG

151 200

igma_BT058702 (1) --------------------------------------------------

igmb_BT059185 (151) TGTCTGGTCTGCCCCTGAGCTGGTTACACTGGATCCGTCAGAAACCAGGG

201 250

igma_BT058702 (1) --------------------------------------------------

igmb_BT059185 (201) AAAGGTCTAGAGTGGATTGGACGCATTGACAGTGGCACTGGCACTATATT

251 300

igma_BT058702 (1) ---------------------------------TTGGGATAGCGCTAATT

igmb_BT059185 (251) TTCCCAGTCTCTACAGGGTCAGTTCACCATCACCAAAGACAACTCCAAAA

301 350

igma_BT058702 (18) TCTATCTGCACAT------GACTCAACTGAAGCCAGAGGACTCTGCAGTG

igmb_BT059185 (301) AACAGCTGTACTTAGAGGTGAAAAGCCTGAAGACTGAAGATTCTGCTGTT

351 400

igma_BT058702 (62) TATTACTGTGCTAGACTCATAACTCTCAACTAC---TTTGACTACTGGGG

igmb_BT059185 (351) TATTATTGTGCCAGAGATCGGGGGCGAGACTACGGTTTTGACTACTGGGG

401 450

igma_BT058702 (109) GAAAGGGACCATGGTGACCGTGTCCACAGCCTCATCAACTGCTCCGACTT

igmb_BT059185 (401) GAAAGGGACAATGGTTACAGTTTCATCAGCCTCATCAACTGCTCCGACTT

451 500

igma_BT058702 (159) TGTTTCCTCTTGCGCAATGTGGCTCCGGGACCGGAGATATGATGACTCTG

igmb_BT059185 (451) TGTTCCCTCTTGCGCAATGTGGCTCCGGGACCGGAGATATGGTGACTCTG

501 550

igma_BT058702 (209) GGTTGCATTGCCACTGGCTTCACGCCTGCCTCCCTCACCTTCAAATGGAA

igmb_BT059185 (501) GGTTGCATTGCCACTGGCTTCACGCCTGCCTCCCTCACCTTCAAATGGAA

551 600

igma_BT058702 (259) TGAACAAGGCGGGAATTCCCTGACTGATTTCGTTCAGTACCCTGCGGTCC

igmb_BT059185 (551) TGAACAAGGCGGAAATTCCCTGACTGATTTCGTTCAGTACCCTGCGGTCC

601 650

igma_BT058702 (309) AAACCAGTGGAAGCTACATGGGAGTCAGTCAACTCCGTGTAAAGAGAGCA

igmb_BT059185 (601) AAACCAGTGGAAGCTACATGGGAGTCAGTCAACTCCGTGTAAAGAGAGCA

651 700

igma_BT058702 (359) GACTGGGACAGTAAAATCTTTGAATGCGCCGTGGAACATTCAGCTGGTTC

igmb_BT059185 (651) GACTGGGACAGTAAAATCTTTGAATGCGCCGTGGAACATTCAGCTGGTTC

701 750

igma_BT058702 (409) AAAGACTGTACCAGTGAAGAAACAAGCGGAATATCTGCAGCACCCGTCTC

igmb_BT059185 (701) AAAGACTGTACCATTGAAGAAACAAGTGGAATATCTGCAGCACCCGTCTC

751 800

igma_BT058702 (459) TTTACGTAATGACCCCCTCTAAAGAGGAGATGGCAGAAAATATGACGGCT

igmb_BT059185 (751) TTTACGTAATGACCCCCTCTAAAGAGGAGATGGCAGAAAATAAGACGGCT

801 850

igma_BT058702 (509) TCCTTCGCCTGCTTTGCCAATGACTTTTCACCCCGTACACACACAATCAA

igmb_BT059185 (801) TCCTTCGCCTGCTTTGCCAATGACTTTTCACCCCGTACACACACAATCAA

851 900

igma_BT058702 (559) ATGGATGAGGATGGAACAAGGAATAGAAAAAGAAGTTGTATCTGATTTCA

igmb_BT059185 (851) ATGGATGAGGATGGAAAAAGGAATAGAAAAAGAAGTTGTATCTGATTTCA

901 950

**C205R051**

igma_BT058702 (609) AGAGTTCTTGTGAGAGTGAGAAGAAGAGTGACAAAACTCTGTACAGCACA

igmb_BT059185 (901) AGAGTTCTTGTGAGAGTGAGAAGAAGAGTGAGAAAACTCTGTACAGCACA

951 1000

igma_BT058702 (659) ACCAGCTATCTCAGGGTCAATGAGAGTGAGTGGAAGAGTGAAGAAGT**AGC**

igmb_BT059185 (951) ACCAGCTATCTCAGGGTCAATGAGAGTGAGTGGAAGAGTGAA**GAAGTTTC**

1001 1050

igma_BT058702 (709) **ATTCACTTGCGTGTTTG**AGAACAAAGCTGGAAATGTGAGGAGAACTGTGG

igmb_BT059185 (1001) **ATTCACTTGCGTGT**TTAAGAACAAAGCTGGAAATGTGAGGAGAACTGTGG

1051 1100

igma_BT058702 (759) GCTACACTTCATCAGATGCAGGTCCAGTCCATGCACATT**CAGTAGTCATT**

igmb_BT059185 (1051) GCTACACTTCATCAGATGCAGGTCCAGTCCATGCACATTCAGT**GGTCATT**

1101 1150

igma_BT058702 (809) **AAGATCACCCCG**CCGTCTCTTGAGGATATGCTTATGAACAAAAAAGCTGA

igmb_BT059185 (1101) **AACATCATCCCGC**CGTCTCTTGAGGATATGCTTATGAACAAAAAAGCTGA

1151 1200

**C061R085**

igma_BT058702 (859) GCTTGTGTGCGATGTCGAGGAACTAGTTCCTGGCTTCATGAGTGTCAAAT

igmb_BT059185 (1151) GCTTGTGTGCGATGTCAAAGAACTAGTTACTGGCTTCATGAGTGTCAAAT

1201 1250

igma_BT058702 (909) GGGAAAATGACAATGGAAAGACCTTAACCAGCCGGAAGGGTGTCACTGAC

igmb_BT059185 (1201) GGGAAAATGACAATGGAAAGACCTTAACCAGCCGGATGGGTGTCACTGAC

1251 1300

igma_BT058702 (959) AGAATTGCCATACTTGACATCACTTATGAGGACTGGAGCAATGGGACAGT

igmb_BT059185 (1251) AAAATTGCCATACTTGACATCACTTATGAGGACTGGAGCAATGGGACAGT

1301 1350

igma_BT058702 (1009) ATTTTACTGCGCTGTAGATCACTTGGAAAACCTGGGGTCCTTGGTAAAGA

igmb_BT059185 (1301) ATTTTACTGCGCTGTAGATCACTTGGAAAACCTGGGGTCCTTGGTAAAGA

1351 1400

igma_BT058702 (1059) AACCCTACAAGAGGGAGACCGGAGGAGATCCACAGCGTCCATCTGTCTTT

igmb_BT059185 (1351) AACCCTACAAGAGGGAGACCGGAGGAGATCCACAGCGTCCATCTGTCTTT

1401 1450

**C075R137**

igma_BT058702 (1109) CTGCTGGCCCCAGCAGAAAAAACTAGTGATAATACGGTGACCCTGACTTG

igmb_BT059185 (1401) CTGCTGGCCCCAGCAGAAAAAACTAGTGATAATACGGTGACCCTGACTTG

1451 1500

igma_BT058702 (1159) CTACGTCAAAGACTTCTACCCCAAGGAAGTTTTAGTGGCTTGGCTTATTG

igmb_BT059185 (1451) CTACGTCAAAGACTTCTACCCCAAGGAAGTTTTAGTGGCTTGGCTTATTG

1501 1550

igma_BT058702 (1209) ATGATGAGCCGGTGGAGAGAACGAGCAGTTCAGCATTGTACCAATTCAAC

igmb_BT059185 (1501) ATGATGAGCCGGTGGAGAGAACGAGCAGTTCAGCATTGTACCAATTCAAC

1551 1600

igma_BT058702 (1259) ACCACTAGCCAGATTCAAACAGGAAGGACCTACTCTGTCTACAGTCAGCT

igmb_BT059185 (1551) ACCACTAGCCAGATTCAATCAGGAAGGACCTACTCTGTCTACAGTCAGCT

1601 1650

igma_BT058702 (1309) CACATTTAGCAATGACTTGTGGAAGAACAAAGAAGTGGTGTATAGCTGTG

igmb_BT059185 (1601) CACATTTAGCAATGACTTGTGGAAGAACAAAGAAGTGGTGTATAGCTGTG

1651 1700

igma_BT058702 (1359) TAGTTTACCACGAAAGCATGATCAAGTCCACAAAAATTCTTATGAGAACC

igmb_BT059185 (1651) TAGTTTACCACGAAAGCATGATCAAGTCCACAAAAATTCTTATGAGAACC

1701 1750

igma_BT058702 (1409) ATTGACAGAACCTCAAACCAACCCAACCTAGTTAACCTCAGCTTGAATGT

igmb_BT059185 (1701) ATTGACAGAACCTCAAACCAACCCAACCTAGTTAACCTCAGCTTGAATGT

1751 1800

igma_BT058702 (1459) GCCTCAGAGCTGCAAGGCCCAGTAGAGGTTGTGTTGTGTTTTGTTGATGT

igmb_BT059185 (1751) GCCTCAGTGCTGCAAGGCCCAGTAGAGGTTGTGTTGTGTTTTGTTGATGT

1801 1850

igma_BT058702 (1509) GTGTTGCTGTGTGTTACCTCTGCTGTTTGTGTTTGTGACATAACCATGTT

igmb_BT059185 (1801) GTGTTGCTGCGTGTTACCTCTGCTGTTTGTGTCAGTGACATAAC-ATGTT

1851 1900

igma_BT058702 (1559) GTGTGTCTTCCAAGTGCAGAATCA--------------------------

igmb_BT059185 (1850) GTGTGTCTTTTAAGTGCAGAATCAAAATAAAAATAAAAACTTTAAATCAT

1901 1945

igma_BT058702 (1583) ---------------------------------------------

igmb_BT059185 (1900) TAAAAAAAAAAAAAAAAAAAACAAAAAAAAAAAAAAAAAAAAAGA
