# Supplementary material for: Changes in the liver transcriptome of farmed Atlantic salmon (Salmo salar) fed experimental diets based on terrestrial alternatives to fish meal and fish oil
Source: BMC Genomics. 2018 Nov 3;19:796. doi: 10.1186/s12864-018-5188-6 (PMC6215684; doi:10.1186/s12864-018-5188-6)
Supplement: Supplementary file 11 — Figure S9. Alignment of nucleotide sequences corresponding to mxa and mxb. Conserved nucleotides in both aligned sequences are highlighted in yellow. Mxa and mxb sequences share 89% identity over 1955 aligned nucleotides. The alignment and percentage identity calculation were performed using AlignX (Vector NTI Advance 11). The nucleotide region covered by the probe C236R043 from the Agilent 44 K salmonid microarray (GEO accession number: GPL11299) is indicated within a box. Forward qPCR primers are in bold and single underlined, whereas reverse qPCR primers are in bold and double underlined. (DOCX 23 kb) [file 12864_2018_5188_MOESM11_ESM.docx]

**Figure S9. Alignment of nucleotide sequences corresponding to *mxa* and *mxb*.**

1 50

mxa_U66475 (1) GGCACGAGGGGACACGCAGGTCGATCAGATAGCAGAACACCTTGCTGTTT

mxb_BT044881 (1) ---------------------------------AGTTGCAGTTGCAGTGT

51 100

mxa_U66475 (51) ATTTAAGTTTTATCACTAAATAATAATTCACAATGAATAACACTCTAAAC

mxb_BT044881 (18) ATTAACCATTTTTT--TTTATCGCAAATC---ATGAATTATACGCTGAAC

101 150

mxa_U66475 (101) CAACATTATGAGGAGAAGGTGCGTCCCTGTATAGACCTCATCGACTCCCT

mxb_BT044881 (63) CAACATTATGAGGAGAAGGTGCGTCCTTGTATTGACCTCATCGACTCCCT

151 200

mxa_U66475 (151) GCGCTCCCTTGGCGTAGAGAAGGACCTTGCGCTGCCAGCCATCGCGGTGA

mxb_BT044881 (113) GCGCTCCCTTGGCGTAGAGAAGGACCTTGCGCTGCCAGCCATCGCGGTGA

201 250

mxa_U66475 (201) TAGGGGACCAGAGTTCAGGGAAGAGCTCAGTGCTGGAGGCGCTATCTGGG

mxb_BT044881 (163) TAGGGGACCAGAGTTCAGGGAAGAGCTCCGTGCTGGAGGCGCTATCTGGG

251 300

mxa_U66475 (251) GTGGCTTTGCCAAGGGGGAGTGGTATTGTAACACGATGCCCTCTCGAGCT

mxb_BT044881 (213) GTGGCTTTGCCAAGGGGGAGTGGTATTGTAACACGATGCCCTCTCGAGCT

301 350

mxa_U66475 (301) GAAGATGAAGAGGAAGAAAGAAGGAGAGGAATGGCATGGAAAAATCAGCT

mxb_BT044881 (263) GAAGATGAAGAGGAAGAAAGAAGGAGAGGAATGGCACGGAAAAATCCGCT

351 400

mxa_U66475 (351) ACCAAGACCATGAGGAGGAGATTGAGGATCCCTCTGATGTGGAGAAGAAA

mxb_BT044881 (313) ACCAAGACCGTGAGGAGGAGATTGAGGACCCCTCTGATGTGGAGAAGAAA

401 450

mxa_U66475 (401) ATTCGTGAAGCCCAGGACGAAATGGCAGGTGTGGGGGTGGGTATCAGTGA

mxb_BT044881 (363) ATTCGGAAAGCCCAGGATGAAATGGCAGGTGTGGGGGTGGGTATCAGTGA

451 500

mxa_U66475 (451) TGACCTCATCAGCCTGGAGATTGGCTCCCCTGACGTCCCAGACCTCACAC

mxb_BT044881 (413) TGACCTCATCAGCCTGGAGATTGGCTCCCCTGACGTCCCAGACCTCACAC

501 550

mxa_U66475 (501) TCATCGACCTGCCAGGCATCGCCCGGGTAGCTGTCAAGGGTCAACCTGAG

mxb_BT044881 (463) TCATCGACCTGCCAGGCATCGCCCGGGTAGCTGTCAAAGGTCAACCTGAG

551 600

mxa_U66475 (551) AACATTGGTGAACAGATTAAGAGACTGATACGGAAGTTCATCACGAAGCA

mxb_BT044881 (513) AACATTGGTGAACAGATTAAGAATCTGATACGCAAGTTCATCACAAAGCA

601 650

mxa_U66475 (601) AGAAACAATCAATTTGGTGGTTGTGCCATGCAACGTTGACATTGCAACCA

mxb_BT044881 (563) AGAAACAATCAACTTGGTGGTTGTGCCATGCAACGTTGACATTGCAACCA

651 700

mxa_U66475 (651) CAGAGGCTTTGAAGATGGCACAAGAGGTGGACCCTGAAGGGGAAAGGACA

mxb_BT044881 (613) CAGAGGCTTTGAAGATGGCACAAGAGGTGGACCCTCAAGGTGGAAGGACA

701 750

mxa_U66475 (701) TTAGGCATCCTGACCAAGCCTGACCTGGTAGACAAAGGCACAGAAGAGAC

mxb_BT044881 (663) TTAGGCATCCTGACCAAGCCTGACCTGGTAGACAAAGGCACAGAAGAGAT

751 800

mxa_U66475 (751) GGTGGTGGACATAGTTCATAATGAGGTTATCCACCTGACTAAGGGCTACA

mxb_BT044881 (713) GGTGGTGGACATAGTTCATAATGAGGTTATCCACCTGACTAAGGGCTACA

801 850

mxa_U66475 (801) TGATAGTCAAGTGCAGGGGCCAGAAGGAGATCATGGAGCGAGTCTCGCTG

mxb_BT044881 (763) TGATAGTCAAGTGCAGGGGCCAGAAGGAGATCATGGAGCAAGTCTCACTG

851 900

mxa_U66475 (851) TCCGAGGCCACAGAGAGGGAGAAGGCTTTCTTCAAAGAGCACGCTCATCT

mxb_BT044881 (813) ACCGAGGCCACAGAGAGGGAGAAGGCCTTCTTCAAAGAGCACCTTCATCT

901 950

mxa_U66475 (901) CAGCACACTATATGATGAGGGCCATGCCACCATCCCTAAACTGGCAGAGA

mxb_BT044881 (863) CAGCACTCTTTATGATGAGGGGCATGCCACCATCCCTAAACTGGCAGAGA

951 1000

mxa_U66475 (951) AATTAACTCTTGAACTAGTGCATCATATTGAGAAATCCCTACCTCGTCTA

mxb_BT044881 (913) AATTAACTCTTGAACTAGTGCAACATATCGAGAAATCCATGCCTCGTCTA

1001 1050

mxa_U66475 (1001) GAAGAGCAGATTGAGGCAAAGCTGGCAGAGACACATGCCGAGCTGGAAAG

mxb_BT044881 (963) AAAGAGCAGATTGAGGAAAAGCTGGAGGAGAC**ACGCACCACTCTGGAGAA**

1051 1100

mxa_U66475 (1051) ATATGGTACCGGGCCACCTGAGGACTCGGCAGAAAGGATGTACTTCCTGA

mxb_BT044881 (1013) **AT**GTGGTACCGGACCCCCTGAAGACCCAAAAGAACGGCAGTATTTTCTGA

1101 1150

mxa_U66475 (1101) TCGATAAAGTGACTGCATTCACCCATGATGCCATTAACCTGAGCACTGGG

mxb_BT044881 (1063) TCGATAAAGTGACTTTGTTCACCCAGGATGTCATTAACCTGAGCACTGGG

1151 1200

mxa_U66475 (1151) GAGGAG**CTGAAAAGCGGAGTTCGTCT**CAACGTCTTTTCCACACTCAGAAA

mxb_BT044881 (1113) GAGGAGCTGAAAAGTGGAGACA---TCAACATCTTCTCCACACTCAGAAC

1201 1250

mxa_U66475 (1201) AGAGTTTGGGAAATGGAAGTTACACCTGGATCACTCTGGAGAAAACTT**TA**

mxb_BT044881 (1160) **AGAGTTCGGGAAATGGAAG**GCACAACTGGATCGCTCTGGAAAGAACTTTA

1251 1300

mxa_U66475 (1251) **ACCAGAGGATCGAGGGAG**AAGTGGCTGATTATGAGAAGACGTACCGTGGA

mxb_BT044881 (1210) ATAAGAAGATTGAAAAAGAAGTGGCTGATTATGAGAAGACGTACCGTGGA

1301 1350

mxa_U66475 (1301) AGGGAGCTCCCAGGGTTCATCAACTACAAGACCTTTGAGGTGATGGTGAA

mxb_BT044881 (1260) AGGGAGCTCCCAGGGTTCATCAACTACAAGACCTTTGAGGTGATGGTGAA

1351 1400

mxa_U66475 (1351) AGACCAGATCAAACAACTGGAGGAACCAGCAGTCAAGAAACTGAAGGAGA

mxb_BT044881 (1310) AGACCAGATCAAACAACTGGAGGAACCAGCAGTCAAGAAGCTGAAAGAGT

1401 1450

mxa_U66475 (1401) TTTCAGATGCCGTTAGGAAGGTGTTCTTACTGCTGGCTCAGAGCAGCTTC

mxb_BT044881 (1360) TATCAGATGTTGCTAGGAAGGCGTTCATACTGCTGGCTCAGAACAGCTTC

1451 1500

mxa_U66475 (1451) ATTGGATTTCCTAACCTCCTGAAATCCGCGAAGACAAAGATTGAGGCCAT

mxb_BT044881 (1410) ACAGGTTTCCCTATTCTCCTGAAAACAGCAAAGACTAAGATCGAGACAAT

1501 1550

mxa_U66475 (1501) TAAGCAGGTGAATGAGTCTACTGCTGAGTCCATGTTGAGGACTCAGTTCA

mxb_BT044881 (1460) CAAGCAGGAGAAGGAGTCTACGGCTGAGTCCATGTTGAGGACTCAGTTCA

1551 1600

mxa_U66475 (1551) AGATGGAGATGATAGTGTACACACAGGACAGCACCTACAGCCACAGTCTG

mxb_BT044881 (1510) AGATGGAGCTGATAGTGTACACACAGGACATCACCTACAGCTCTAGCCTG

1601 1650

mxa_U66475 (1601) AGTGAGAGGAAGAGGGAGGAGGAAGAC------GACCGACCCTTA-----

mxb_BT044881 (1560) AGGAAGAGGAAGAGGGAGGAGGAAGAGTTGGAGGAGGGAGAGTTAGTTAA

1651 1700

mxa_U66475 (1640) ----CCGACCATTA------------AGA---------------------

mxb_BT044881 (1610) AAATCCTTCCCTTTCTTTTGGGAGTCAGAAAGTGTTATCTGTTTTCTCTG

1701 1750

mxa_U66475 (1653) TAAGGAGTACAATCTTTAGCACAGACAACCATGCCACCCTACAGGAGATG

mxb_BT044881 (1660) TAAGGAGTACTGTCAACGGCCATGACAACCATGCTGCCCTACGGGAGATG

1751 1800

mxa_U66475 (1703) ATGCTGCACCTCAAGTCCTATTACAGGATATCCAGTCAACGTTTGGCTGA

mxb_BT044881 (1710) ATGCTGCACCTCAAGTCCTATTATAATATTGCCAGTCAGCGTCTGGCTGA

1801 1850

mxa_U66475 (1753) TCAGATTCCCATGGTGATCCGCTACCTGGTGCTGCAGGAGTTTGCTTCCC

mxb_BT044881 (1760) TCAGATTCCCATGGTGATCCGCTACCTGGTTCTGCAGGAGTTTGCTTCCC

1851 1900

mxa_U66475 (1803) AGCTGCAGAGGGAGATGCTTCAGACTCTGCAGGAGAAGGACAACATCGAG

mxb_BT044881 (1810) AGCTGCAGAGGGAGATGCTTCAGACTCTGCAGGAGAAGGACAACATCGAG

1901 1950

mxa_U66475 (1853) CAGCTGCTGAAGGAGGACTTCGACATCGGCAGCAAGAGGGCTGCACTGCA

mxb_BT044881 (1860) CAGCTGCTGAAGGAGGACATCGACATCGGCAGCAAGAGGGCTTCACTGCA

1951 2000

mxa_U66475 (1903) GAACAAGCTCAAACGTCTGATGAAGGCACGCAGCTACCTAGTTGAGTTCT

mxb_BT044881 (1910) GAGCAAGCTCAAACGTCTGATGAAGGCACGCAGCTACCTAGTGGAGTTCT

2001 2050

mxa_U66475 (1953) AGTATGGACAGCTGCTTGTTAACATTTAGGATGGTCTTGATTTTTTTTAA

mxb_BT044881 (1960) AGTATGGACAGCTGCTTGTTAACATTTAGGATGGTCT------------A

**C236R043**

2051 2100

mxa_U66475 (2003) ATGTAATGTATTTTTTGGGAGGATAACAGACATATACAACAAAATGTACA

mxb_BT044881 (1998) ATTTCAGATAGCTTGGTCAATGGAAGTAGGCAT-TGGGGTTTGGGGTAGA

2101 2150

mxa_U66475 (2053) ATGTGGACCCAGAGGATATCACTTGAAAGTGTTAATTAAAAAGCTTGTTT

mxb_BT044881 (2047) TTCTGTTATC------TAACTTATAATAAAGTTACTATGCAATCAAAAAA

2151 2180

mxa_U66475 (2103) CCAAAAAAAAAAAAAAAAAAAAAAAAAAA-

mxb_BT044881 (2091) AAAAAAAAAAAAAAAAAAAAAAAAAAAAGA
