# Supplementary material for: Changes in the liver transcriptome of farmed Atlantic salmon (Salmo salar) fed experimental diets based on terrestrial alternatives to fish meal and fish oil
Source: BMC Genomics. 2018 Nov 3;19:796. doi: 10.1186/s12864-018-5188-6 (PMC6215684; doi:10.1186/s12864-018-5188-6)
Supplement: Supplementary file 12 — Figure S10. Alignment of nucleotide sequences corresponding to two mtco2 paralogues and the probe C060R108 from the Agilent 44 K salmonid microarray (GEO accession number: GPL11299). Conserved nucleotides in all three aligned sequences are highlighted in yellow, those conserved in two are highlighted in blue. Mtco2a and mtco2b sequences share 96% identity over 547 aligned nucleotides, and 83 and 85% identity compared with the 60mer microarray probe, respectively. The alignment and percentage identity calculation were performed using AlignX (Vector NTI Advance 11). Forward qPCR primers are in bold and single underlined, whereas reverse qPCR primers are in bold and double underlined. (DOCX 13 kb) [file 12864_2018_5188_MOESM12_ESM.docx]

**Figure S10. Alignment of nucleotide sequences corresponding to two *mtco2* paralogues and the probe C060R108 from the Agilent 44K salmonid microarray (GEO accession number: GPL11299).**

1 50

mtco2a_BT044012 (1) CACAACTAGGATTCCAAGACGCGGCCTCCCCTGTAATAGAAGAACTCCTT

mtco2b_DW556807 (1) --------------------------------------------------

C060R108_mtco2 (1) --------------------------------------------------

51 100

mtco2a_BT044012 (51) CATTTTCACGACCATGCTCTTATGATTGTTCTTCTTATCAGCACACTAGT

mtco2b_DW556807 (1) --------------------------------------------------

C060R108_mtco2 (1) --------------------------------------------------

101 150

mtco2a_BT044012 (101) GCTTTATATCATTGTAGCAATAGTCTCTACTAAACTCACTAACAAGTATA

mtco2b_DW556807 (1) -------------------------------AAACTCACTAACAAGTATA

C060R108_mtco2 (1) --------------------------------------------------

151 200

mtco2a_BT044012 (151) TCCTTGATTCTCAAGAAATCGAAATCGTTTGGACTGTCCTTCCAGCAGTT

mtco2b_DW556807 (20) TCCTTGATTTTCAAGAAATTGAAATCGTTTGGACTGTCCTTCCAGCAGTT

C060R108_mtco2 (1) --------------------------------------------------

201 250

mtco2a_BT044012 (201) ATCCTCATTCTTATTGCCCTCCCCTCCCTTCGAATTCTTTACCTTATAGA

mtco2b_DW556807 (70) ATCCTCATTTTTATCGCCCTCCCCTCCCTTCGAATTTTTTCCCTTATAGA

C060R108_mtco2 (1) --------------------------------------------------

251 300

mtco2a_BT044012 (251) **CGAAATTAATGACCCACACCTTA**CTATTAAAGCAATGGGTCACCAATGAT

mtco2b_DW556807 (120) GGAAATTAATGACCCACCCCTTATTATTAAAGCAATGGGTCACCAATGAT

C060R108_mtco2 (1) --------------------------------------------------

301 350

mtco2a_BT044012 (301) ACTGAAGCTATGAATACACCGACTACGAAGACTTAGGCTTTGACTCTTAT

mtco2b_DW556807 (170) ACTGAAGCTATGAATACCCCGACT**ACGAAGACTTGGGCTTTGATT**TTTAT

C060R108_mtco2 (1) --------------------------------------------------

351 400

mtco2a_BT044012 (351) ATAGTCCCCACCCAAGACTTAACGCCCGGTCAATTTCGTCTTCTAGAAAC

mtco2b_DW556807 (220) ATAGTCCCCACCCAAGACTTAACGCCCGGTCAATTTTGTTTTTTAGAAAC

C060R108_mtco2 (1) --------------------------------------------------

401 450

mtco2a_BT044012 (401) AGACCATCGAATGGTTGTCCCTG**TAGAATCTCCAATCCGCGTC**CTAGTTT

mtco2b_DW556807 (270) AGACCATCGAATGGTTGTCCCTGT**AGAATTTCCAATCCGCGTC**CTAGTTT

C060R108_mtco2 (1) --------------------------------------------------

451 500

mtco2a_BT044012 (451) CAGCTGAAGACGTCCTTCACTCCTGAGCCGTCCCTTCCTTAGGTGTAAAA

mtco2b_DW556807 (320) CAGCTGAAGACGTCCTTCACTCCTGAGCCGTCCCTTCCTTAGGTGTAAAA

C060R108_mtco2 (1) --------------------------------------------------

501 550

mtco2a_BT044012 (501) ATGGACGCAGTCCCAGGACGATTAAACCAAACAGCCTTTATTGCCTCTCG

mtco2b_DW556807 (370) ATGGACGCAGTCCCAGGAGGATTAAACCAAACAGCCTTTATTGCCTCTCG

C060R108_mtco2 (1) --------------------------------------------------

551 600

mtco2a_BT044012 (551) ACCTGGAGTATTCTACGGACAATGTTCTGAAATCTGCGGGGCCAACCACA

mtco2b_DW556807 (420) ACCTGGAGTATTTTACGGACAATGTTTTGAAATTTGGGGGGCCAACCACA

C060R108_mtco2 (1) --------------------------------------------------

601 650

mtco2a_BT044012 (601) GCTTCATACCCATCGTTGTTGAAGCAGTGCCCCTAGAACACTTCGAGAAA

mtco2b_DW556807 (470) GCTTCATACCCATGGTTGTTGAAGCAGTACCCCTAGAACACTTCGAGAAA

C060R108_mtco2 (1) ------------------TTGAAGGGGTACCCCTAGAACATTTTGGGAAA

651 684

mtco2a_BT044012 (651) TGATCCACTATAATACTTGAAGATGCCTAAAAAA

mtco2b_DW556807 (520) TGATCCACTATAATACTTGAAGATGCCT------

C060R108_mtco2 (33) TGATCCATTTTGATATTTGAAGATGCCT------
