# Supplementary figures and images for: Changes in the liver transcriptome of farmed Atlantic salmon (Salmo salar) fed experimental diets based on terrestrial alternatives to fish meal and fish oil
Source: BMC Genomics. 2018 Nov 3;19:796. doi: 10.1186/s12864-018-5188-6 (PMC6215684; doi:10.1186/s12864-018-5188-6)

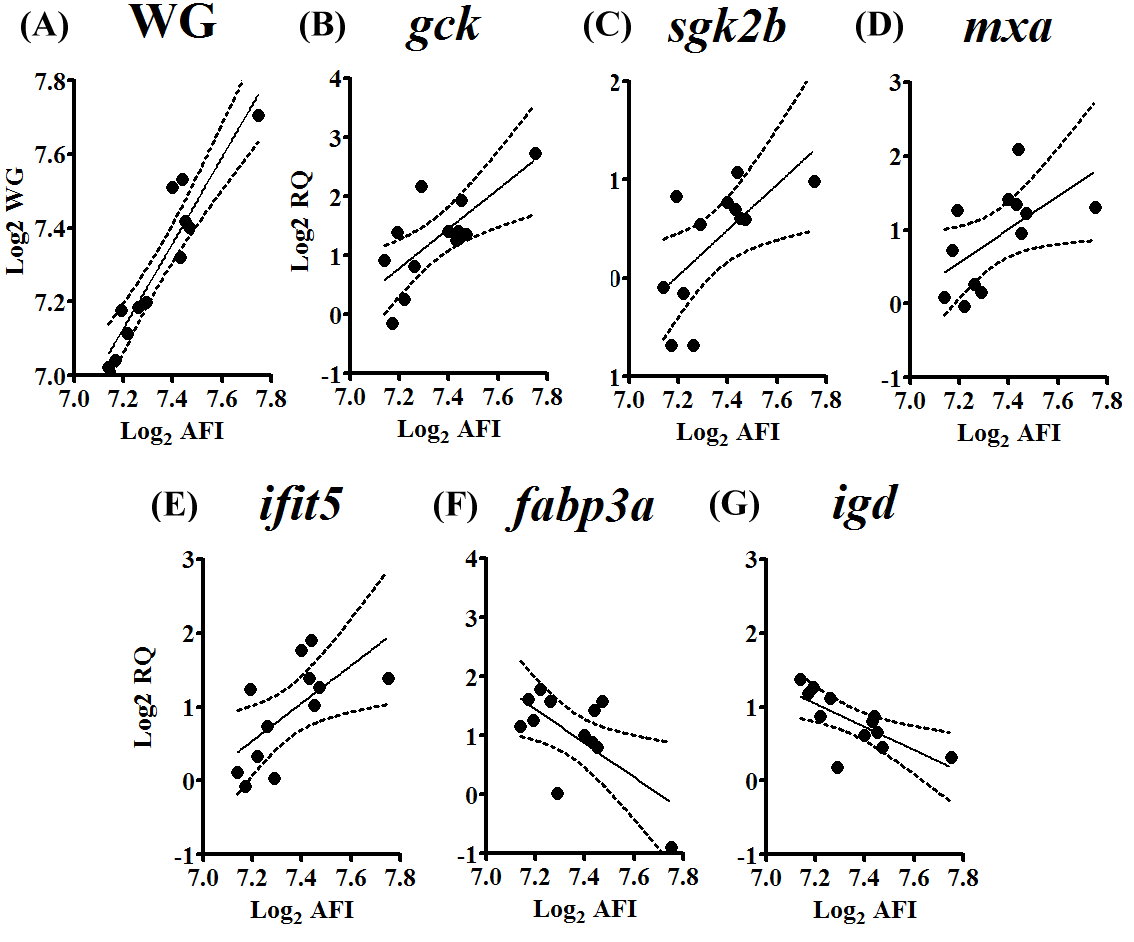

Supplement: Supplementary file 13 — Figure S11. Scatter plots showing linear correlations between log2 apparent feed intake (AFI) and: A) log2 weight gain (WG); B) log2 gck RQs; C) log2 sgk2b RQs; D) log2 mxa RQs; E) log2 ifit5 RQs; F) log2 fabp3a RQs; G) log2 igd RQs. The analyses were performed using the tank mean values of each variable as AFI could only be calculated by tank. Regression lines and 95% confidence intervals are represented by solid and dashed lines, respectively. (TIF 95 kb) [file 12864_2018_5188_MOESM13_ESM.tif]
